# Supplementary material for: Giant worms chez moi! Hammerhead flatworms (Platyhelminthes, Geoplanidae, Bipalium spp., Diversibipalium spp.) in metropolitan France and overseas French territories
Source: PeerJ. 2018 May 22;6:e4672. doi: 10.7717/peerj.4672 (PMC5969052; doi:10.7717/peerj.4672)
Supplement: Supplemental Information 1 — Photographs and details about bipaliines with molecular data, listed in Table 2. [file peerj-06-4672-s001.pdf]

Supplement 1 to article:
Photographs of specimens with molecular data

Giant worms *chez moi!* Hammerhead flatworms (Platyhelminthes, Geoplanidae, *Bipalium* spp., *Diversibipalium* spp.) in metropolitan France and overseas French territories

Jean-Lou Justine [1*], Leigh Winsor [2], Delphine Gey [3], Pierre Gros [4] and Jessica Thévenot [5]

1 Institut Systématique Évolution Biodiversité (ISYEB), Muséum National d'Histoire Naturelle, CNRS, Sorbonne Université, EPHE, 57 rue Cuvier, CP 51, 75005 Paris, France

2 College of Science and Engineering, James Cook University, Townsville, Australia

3 Service de Systématique Moléculaire, Muséum National d'Histoire Naturelle, Paris, France

4 Amateur Naturalist, Cagnes-sur-Mer, France

5 Coordination technique et scientifique de la stratégie nationale relative aux espèces exotiques envahissantes, UMS Patrinat, Muséum National d'Histoire Naturelle, Sorbonne Universités, Paris, France

PeerJ 6 : e4672 (2018)

DOI : 10.7717/peerj.4672

Reports of sighting of land planarians were received from citizens, mainly by email, sometimes by telephone. Photographs and details about locality were solicited, and only reports including this information were considered. Wrong records (slugs, myriapods, earthworms, leeches, caterpillars, nematomorphs, and nemerteans) were eliminated. Information collected from citizen science allowed monitoring of several land planarians (Justine et al. 2014a). Photographs were studied, and species were identified whenever possible. Only information relative to bipaliines is reported in this paper). Sometimes citizens provided records dating from before the survey, such as an amateur movie taken in 1999. Most citizens provided an authorisation to use the photographs at the time of the initial contact by email, and most photographs were posted to the twitter account @Plathelminthe4 https://twitter.com/Plathelminthe4.

When we prepared this paper for publication, we sought authorization to use the photographs and to publish them under a Creative Commons Licence; only one of the citizens refused to provide the authorization, but some of them did not respond, probably simply because they changed their emails or did not check them. In these cases, we provide the scientific information about the presence of species, but we do not include the photograph of the worm or the name of the citizen in the paper; these records are marked “consent not obtained”.

Supplement 1 to article: Photographs of specimens with molecular data

Specimens with molecular data.

BK: *Bipalium kewense*; BV: *Bipalium vagum*; DM: *Diversibipalium multilineatum*; Dblue: *Diversibipalium* sp. 'blue'; Dblack: *Diversibipalium* sp. 'black'.

Species	MNHN	date	Locality	Department / State	Country - Continent	Collector
BK	JL089	12/11/2013	Saint Pée sur Nivelle	Pyrénées-Atlantiques	Met. France - Europe	Consent not obtained
BK	JL160	23/05/2014	Cannes	Alpes-Maritimes	Met. France - Europe	Iachia, Valeria
BK	JL167	24/08/2014	Orthez	Pyrénées-Atlantiques	Met. France - Europe	Rougeux, Christian
BK	JL174	03/09/2014	Bassussary	Pyrénées-Atlantiques	Met. France - Europe	Mercader, Elisabeth
BK	JL176 **	05/09/2014	Auxerre (hothouse)	Yonne	Met. France - Europe	Bellina, Arnaud
BK	JL184	Oct. 2014	Ustaritz	Pyrénées-Atlantiques	Met. France - Europe	Goyheneche, Iker
BK	JL188	08/10/2014	Miramar	Grande Porto	Portugal - Europe	Soarès, Luciana
BK	JL212	19/12/2014	Mimbastes	Landes	Met. France - Europe	Jouveau, Séverin
BK	JL224	23/02/2015	Trois Rivières	Guadeloupe	Guadeloupe - C. America	Van Laere, Guy
BK	JL233	27/09/2014	Monaco	Monaco	Monaco - Europe	Dusoulier, François
BK	JL253	21/03/2015	Trois Rivières	Guadeloupe	Guadeloupe - C. America	Van Laere, Guy
BK	JL254	15/05/2015	Matoury	French Guiana	French Guiana - S. America	Girault, Rémi
BK	JL270	23/04/2015	Ducos	Martinique	Martinique - C. America	Lucas, Pierre-Damien
BK	JL308	08/09/2016	Morne Vert	Guadeloupe	Guadeloupe - C. America	Coulis, Mathieu
BV	JL073	Aug. 2013	Sanibel	Florida	USA - North America	Justine, Jean-Lou

BV	JL163	July 2014	Sanibel	Florida	USA - North America	Justine, Jean-Lou
BV	JL164	July 2014	Sanibel	Florida	USA - North America	Justine, Jean-Lou
BV	JL213	29/11/2014	Anse-Bertrand	Guadeloupe	Guadeloupe - C. America	Charles, Laurent
BV	JL268	Dec. 2014	Montserrat	Montserrat	Montserrat - C. America	Shoobs, Nathaniel F.
BV	JL307	19/11/2015	Morne Vert	Guadeloupe	Guadeloupe - C. America	Coulis, Mathieu
DM	JL177 *	30/09/2014	Léguevin	Haute-Garonne	Met. France - Europe	Chaim, Florence
DM	JL059	15/06/2013	La Bastide de Serou	Ariège	Met. France - Europe	Brugnara, Sébastien
DM	JL142	22/04/2014	Saubrigues	Landes	Met. France - Europe	Robineau, Thiérry
DM	JL161	11/06/2015	Bellocq	Pyrénées-Atlantiques	Met. France - Europe	Audiot, Marie-Claude
DM	JL208	11/06/2014	Bellocq	Pyrénées-Atlantiques	Met. France - Europe	Audiot, Marie-Claude
DM	JL209	12/06/2014	Bellocq	Pyrénées-Atlantiques	Met. France - Europe	Audiot, Marie-Claude
DM	JL210	June 2014	Bellocq	Pyrénées-Atlantiques	Met. France - Europe	Audiot, Marie-Claude
DM	JL298 ***	01/06/2016	Novazzano	Ticino Canton	Switzerland - Europe	Pollini, Lucia
DBlue	JL280	2015	Mtsamboro	Mayotte	Mayotte - Africa	Charles, Laurent
DBlue	JL281	29/04/2015	Mtsamboro	Mayotte	Mayotte - Africa	Charles, Laurent
DBlue	JL282	30/04/2015	Ouangani	Mayotte	Mayotte - Africa	Charles, Laurent
DBlue	JL284	05/05/2015	Mtsamboro	Mayotte	Mayotte - Africa	Charles, Laurent
DBlack	JL090	12/11/2013	Saint Pée sur Nivelle	Pyrénées-Atlantiques	Met. France - Europe	Consent not obtained

* JL177 already published (Mazza et al. 2016); ** specimen from hot house, all others are from the open; *** Specimen MCSN 719.990/77.590 kept in Museo Cantonale di Storia Naturale, Lugano, Switzerland, forwarded by Jean Mariaux (Geneva, Switzerland).

Bipalium kewense MNHN JL089

Species	MNHN	date	Locality	Department / State	Collector
BK	JL089	12/11/2013	Saint Pée sur Nivelle	Pyrénées-Atlantiques	Consent not obtained

Note: length of animal = 21 cm

Bipalium kewense MNHN JL160

Species	MNHN	date	Locality	Department / State	Collector
BK	JL160	23/05/2014	Cannes	Alpes-Maritimes	Iachia, Valeria

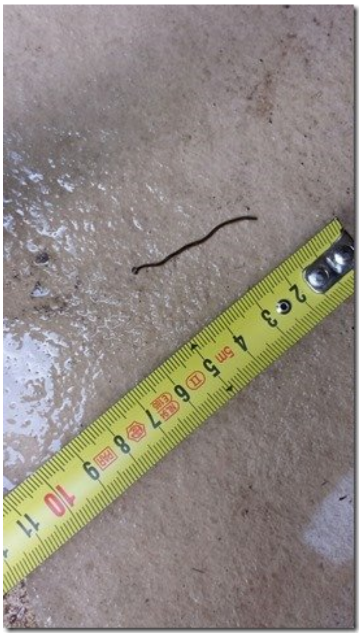

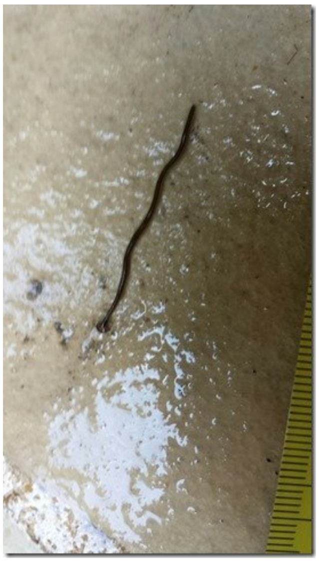

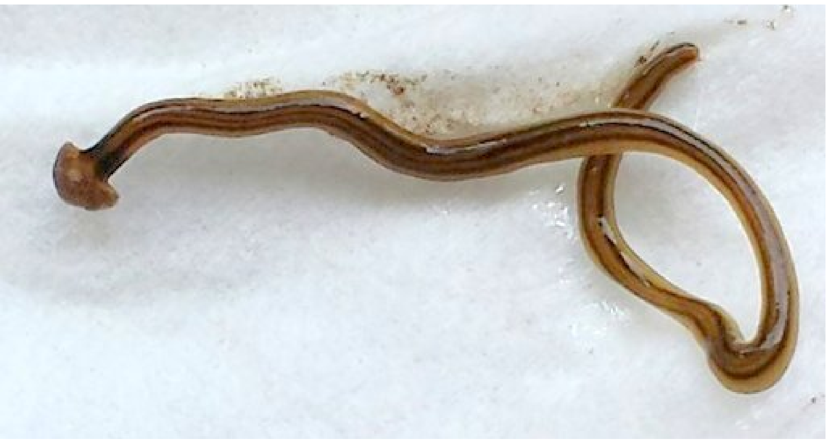

Bipalium kewense MNHN JL167

Species	MNHN	date	Locality	Department / State	Collector
BK	JL167	24/08/2014	Orthez	Pyrénées-Atlantiques	Rougeux, Christian

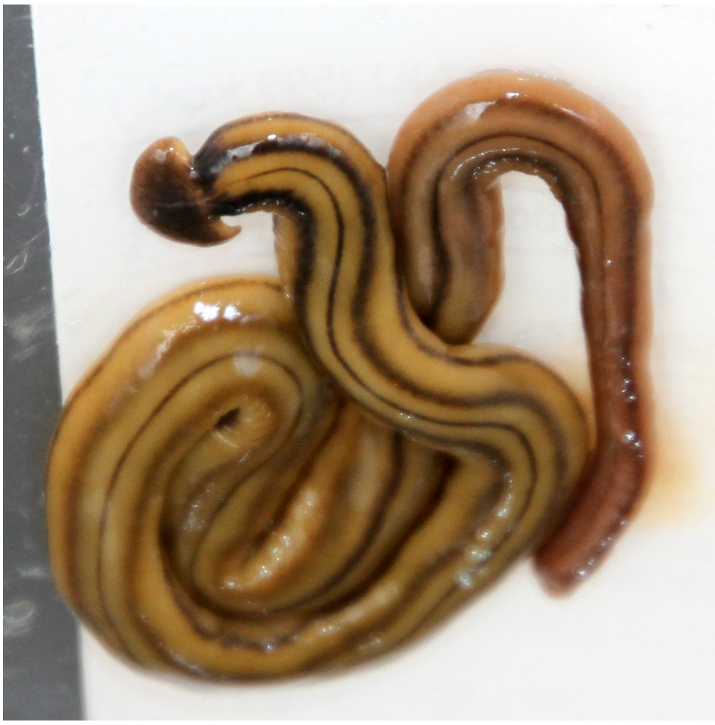

Bipalium kewense MNHN JL174

Species	MNHN	date	Locality	Department / State	Collector
BK	JL174	03/09/2014	Bassussary	Pyrénées-Atlantiques	Mercader, Elisabeth

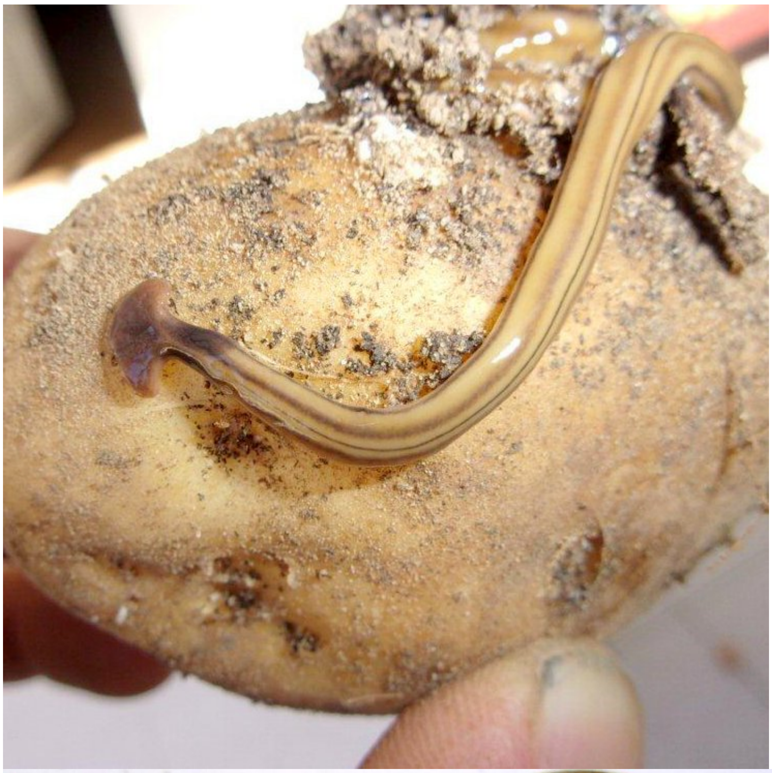

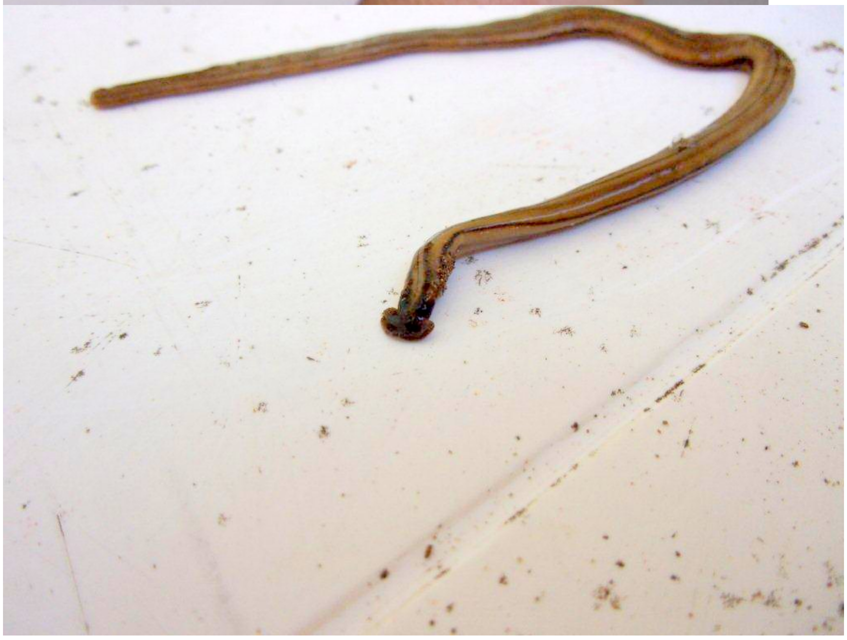

Bipalium kewense MNHN JL176

Species	MNHN	date	Locality	Department / State	Collector
BK	JL176	05/09/2014	Auxerre (hothouse)	Yonne	Bellina, Arnaud

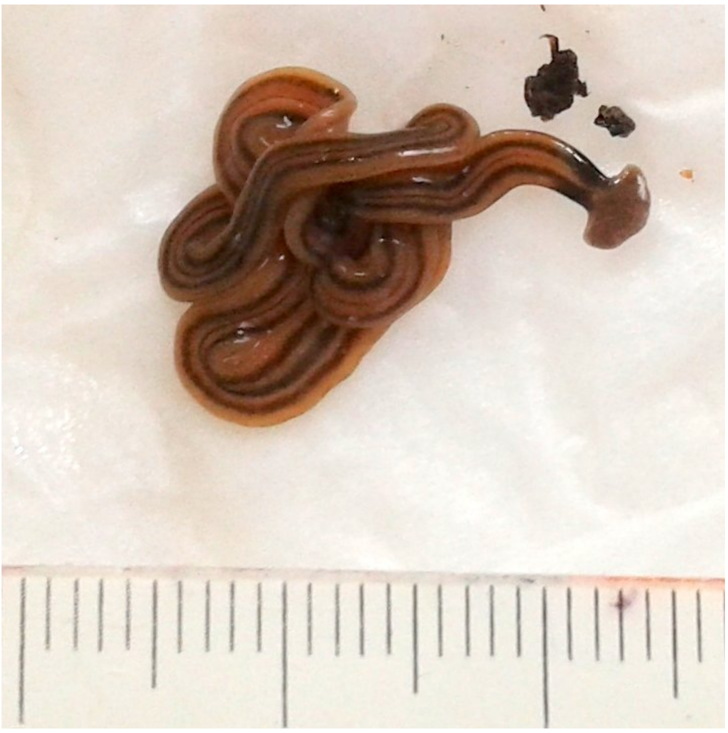

Bipalium kewense MNHN JL184

Species	MNHN	date	Locality	Department / State	Collector
BK	JL184	Oct. 2014	Ustaritz	Pyrénées-Atlantiques	Goyheneche, Iker

Note: length of animal = ca 16 cm

Note: These are specimens of the same population.

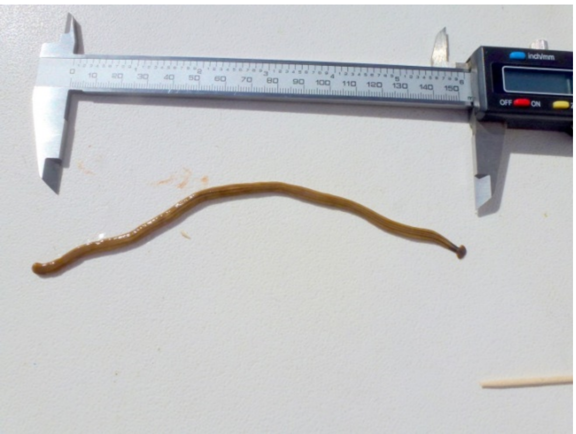

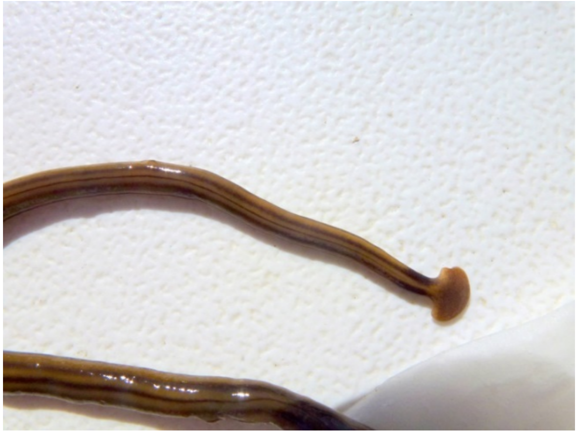

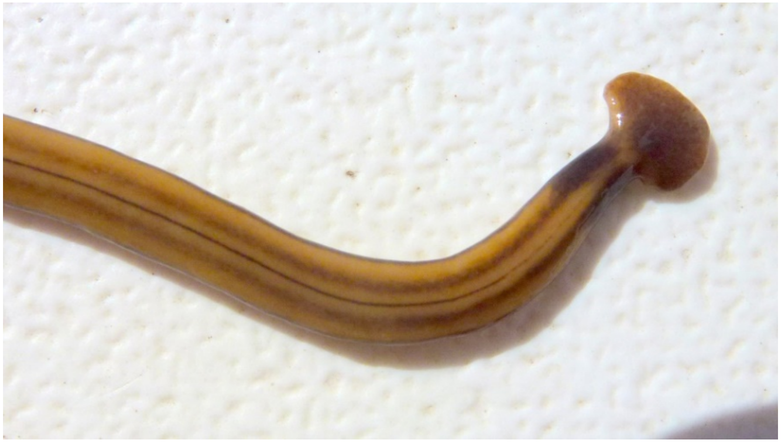

Bipalium kewense MNHN JL188

Species	MNHN	date	Locality	Department / State	Collector
BK	JL188	08/10/2014	Miramar	Grande Porto	Soarès, Luciana

Note: length of animal = 25 cm

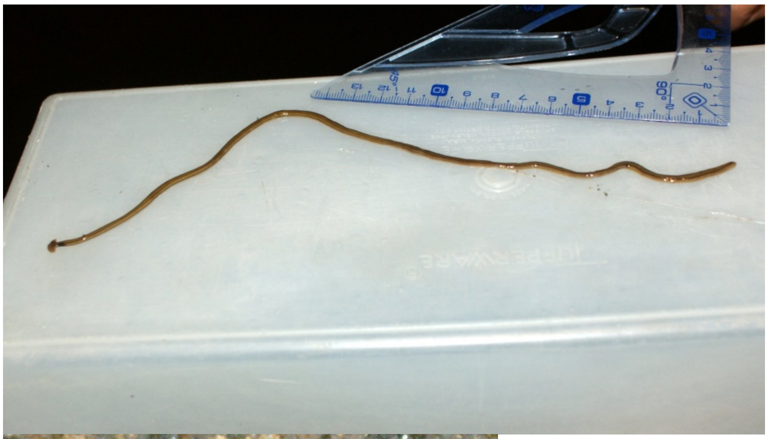

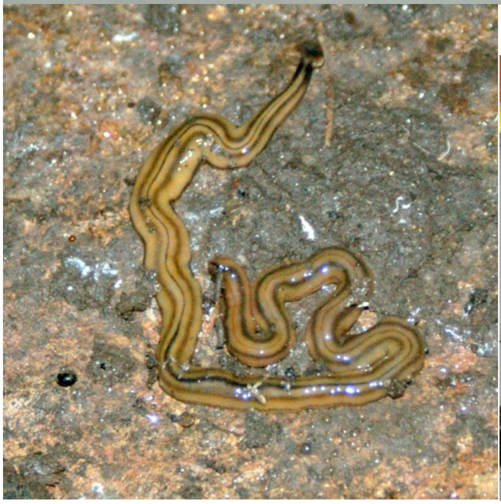

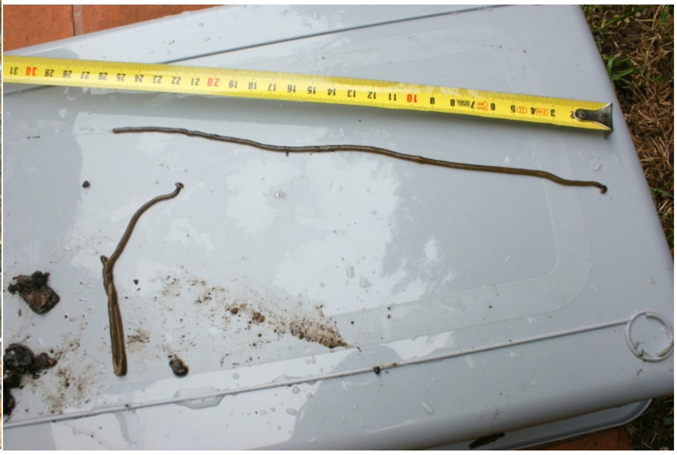

Bipalium kewense MNHN JL212

Species	MNHN	date	Locality	Department / State	Collector
BK	JL212	19/12/2014	Mimbastes	Landes	Jouveau, Séverin

No photograph provided

Bipalium kewense MNHN JL224

Species	MNHN	date	Locality	Department / State	Collector
BK	JL224	23/02/2015	Trois Rivières	Guadeloupe	Van Laere, Guy

Note: length of animal = ca. 21 cm

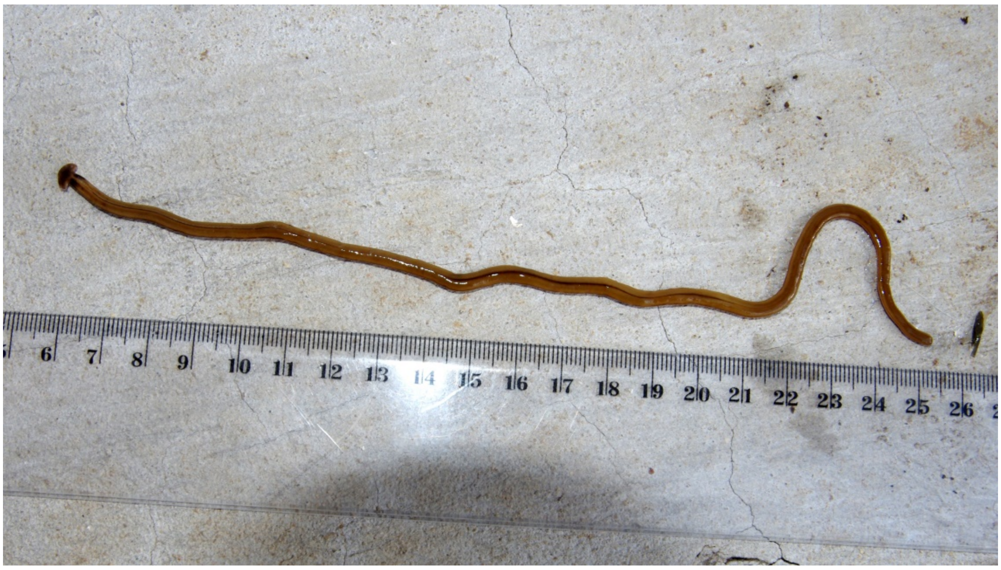

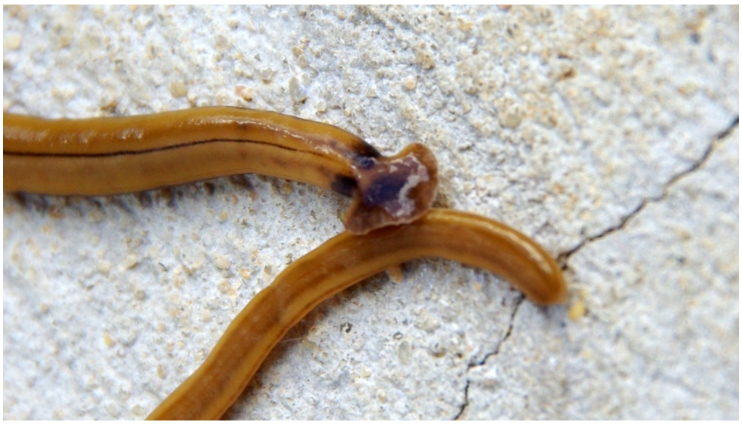

Bipalium kewense MNHN JL233

Species	MNHN	date	Locality	Department / State	Collector
BK	JL233	27/09/2014	Monaco	Monaco	Dusoulier, François

No photograph provided

Bipalium kewense MNHN JL253

Species	MNHN	date	Locality	Department / State	Collector
BK	JL253	21/03/2015	Trois Rivières	Guadeloupe	Van Laere, Guy

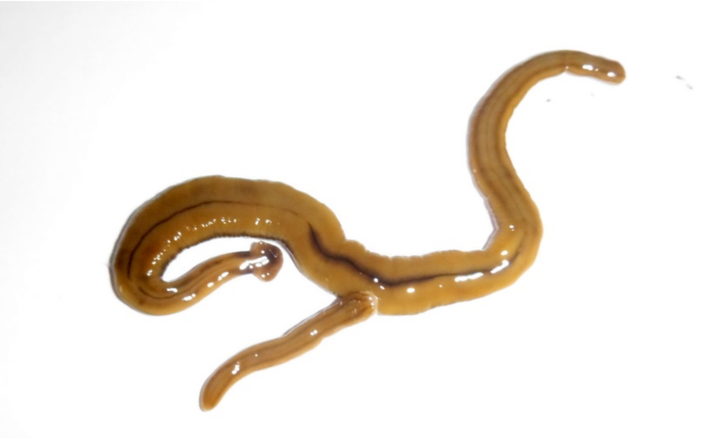

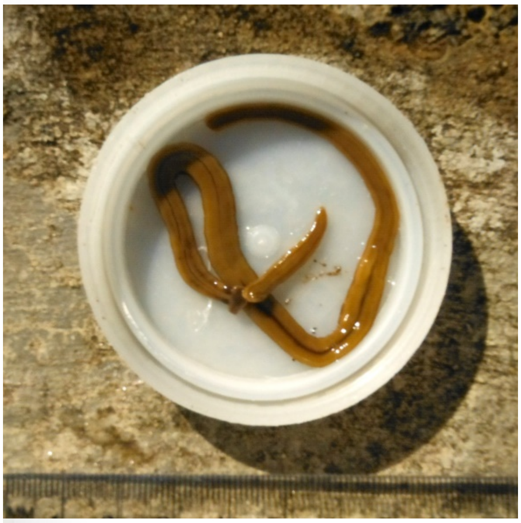

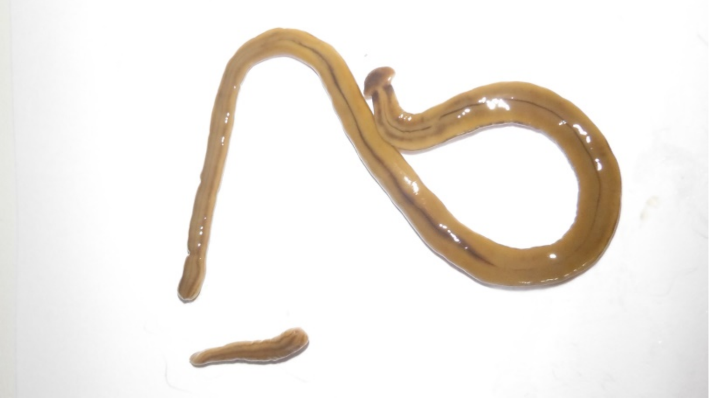

Bipalium kewense MNHN JL254

Species	MNHN	date	Locality	Department / State	Collector
BK	JL254	15/05/2015	Matoury	French Guiana	Girault, Rémi

No photograph provided for this specimen.

Bipalium kewense MNHN JL270

Species	MNHN	date	Locality	Department / State	Collector
BK	JL270	23/04/2015	Ducos	Martinique	Lucas, Pierre-Damien

Note: length of animal = 11 cm

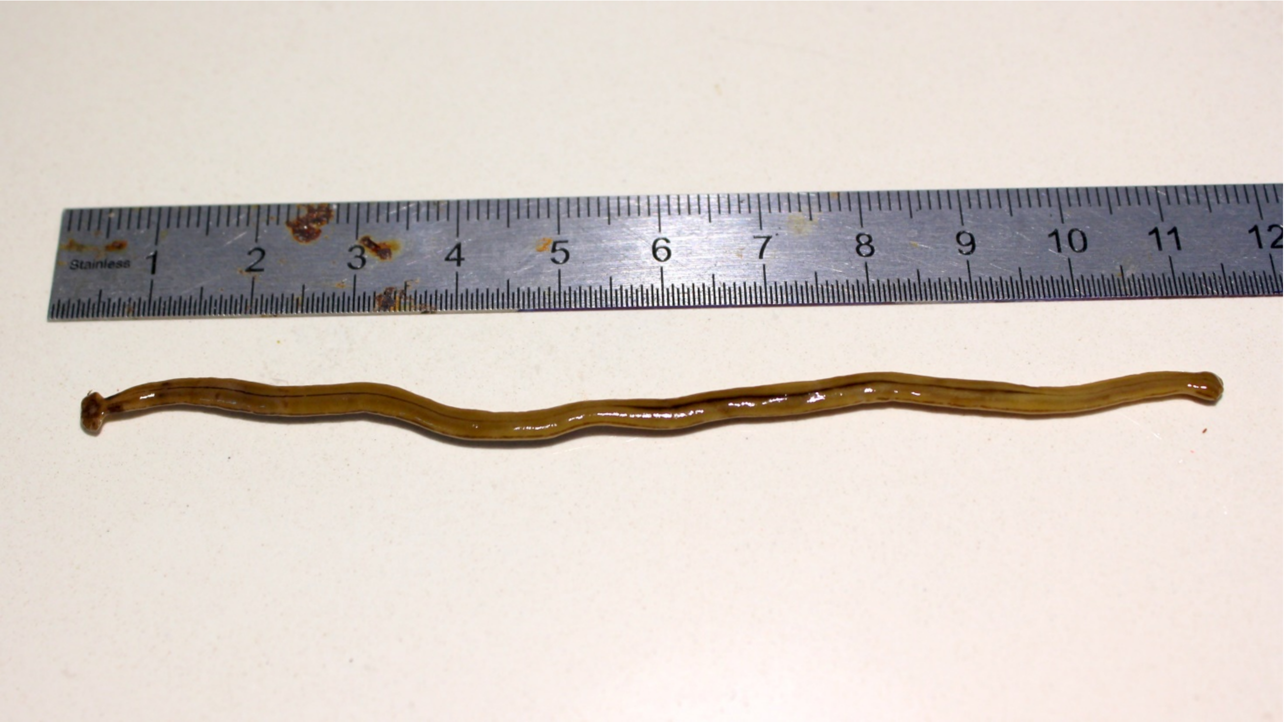

Bipalium kewense MNHN JL308

Species	MNHN	date	Locality	Department / State	Collector
BK	JL308	08/09/2016	Morne Vert	Guadeloupe	Coulis, Mathieu

(Photographs of preserved specimen by Jean-Lou Justine)

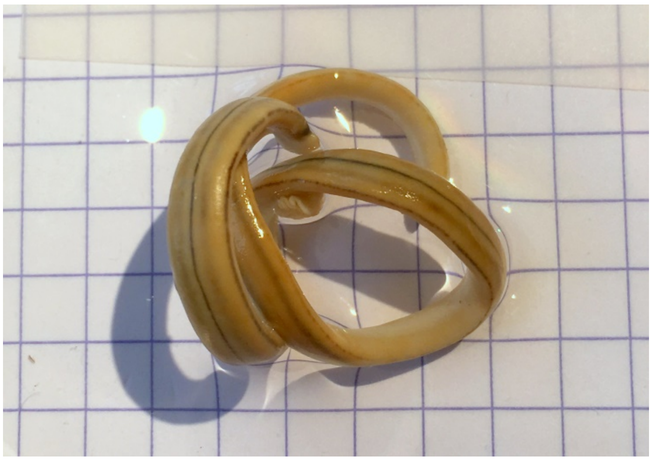

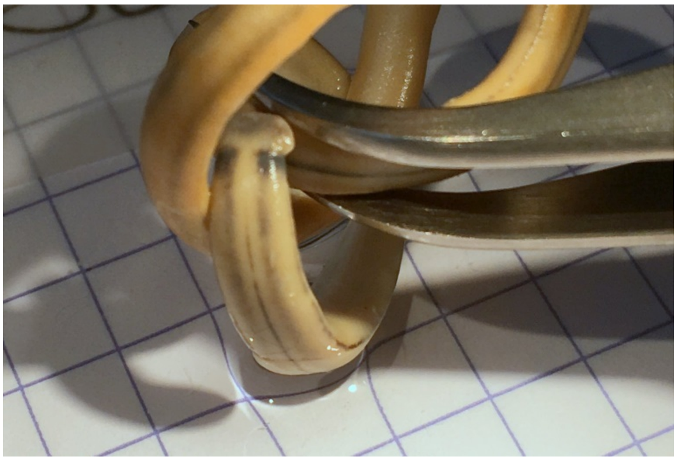

Bipalium vagum MNHN JL073

Species	MNHN	date	Locality	Department / State	Collector
BV	JL073	Aug. 2013	Sanibel	Florida	Justine, Jean-Lou

No photograph of live specimen.

Bipalium vagum MNHN JL163

Species	MNHN	date	Locality	Department / State	Collector
BV	JL163	July 2014	Sanibel	Florida	Justine, Jean-Lou

No photograph of live specimen.

Bipalium vagum MNHN JL164

Species	MNHN	date	Locality	Department / State	Collector
BV	JL164	July 2014	Sanibel	Florida	Justine, Jean-Lou

No photograph of live specimen.

Bipalium vagum MNHN JL213

Species	MNHN	date	Locality	Department / State	Collector
BV	JL213	29/11/2014	Anse-Bertrand	Guadeloupe	Charles, Laurent

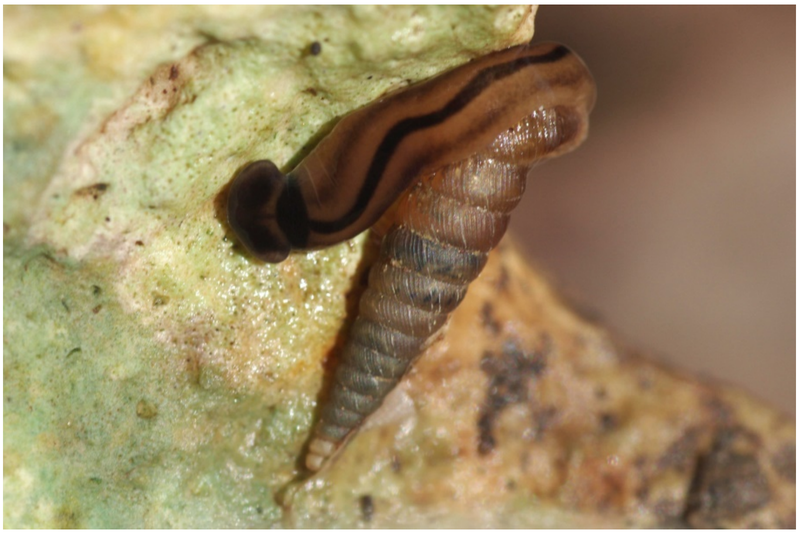

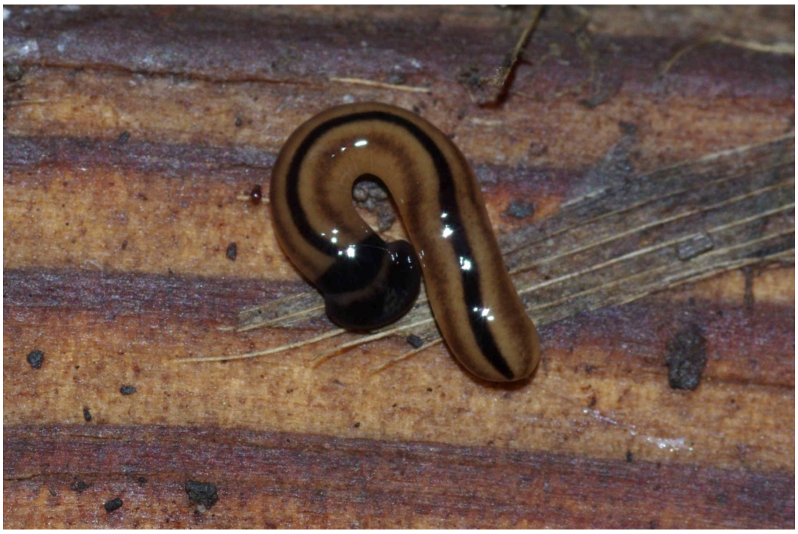

Bipalium vagum MNHN JL268

Species	MNHN	date	Locality	Department / State	Collector
BV	JL268	Dec. 2014	Montserrat	Montserrat	Shoobs, Nathaniel F.

No photograph of live specimen.

Bipalium vagum MNHN JL307

Species	MNHN	date	Locality	Department / State	Collector
BV	JL307	19/11/2015	Morne Vert	Guadeloupe	Coulis, Mathieu

No photograph of live specimen. Photographs of preserved specimen by Jean-Lou Justine. Scale: squares = 5 mm.

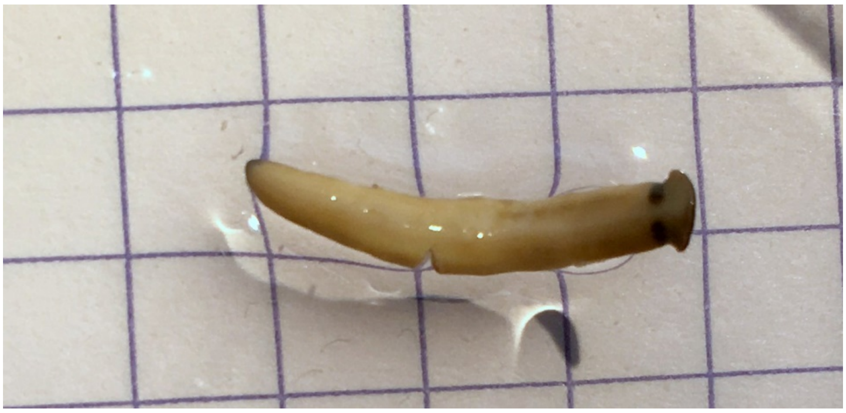

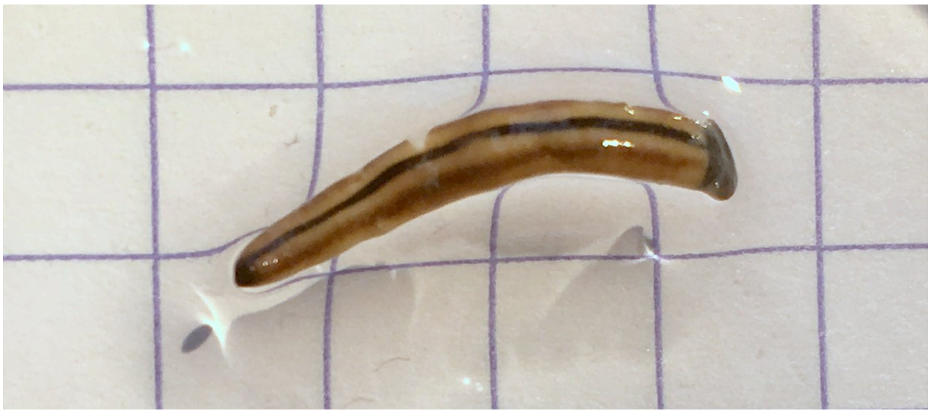

Diversibipalium multilineatum MNHN JL177

Species	MNHN	date	Locality	Department / State	Collector
DM	JL177	30/09/2014	Léguevin	Haute-Garonne	Chaim, Florence

Note: length of animal = ca 15 cm

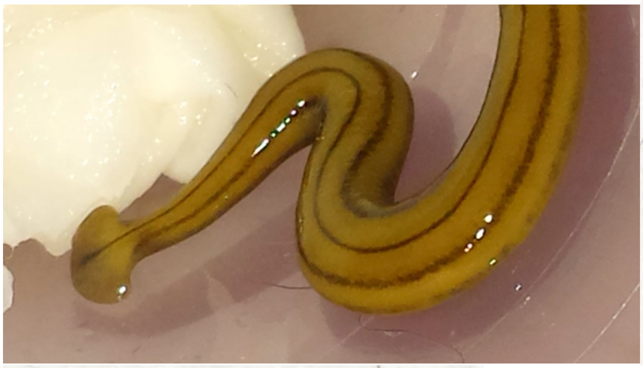
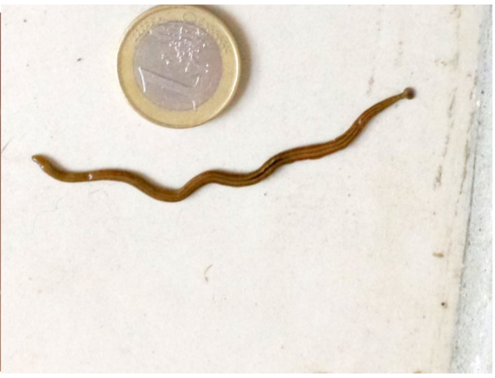
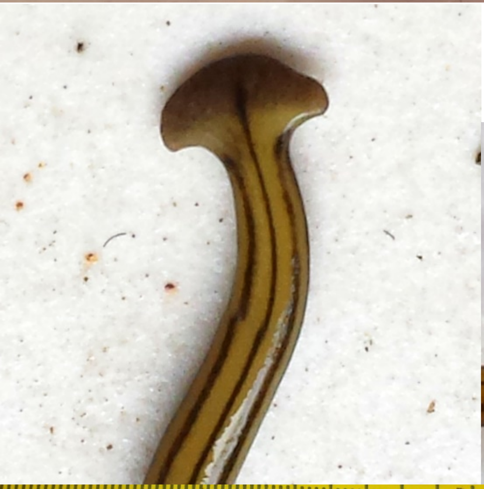
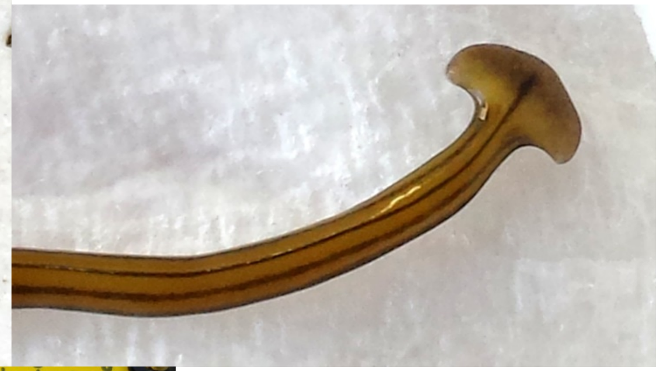
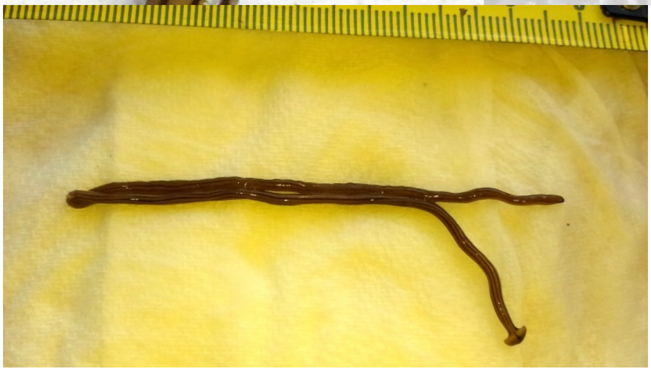

Diversibipalium multilineatum MNHN JL059

Species	MNHN	date	Locality	Department / State	Collector
DM	JL059	15/06/2013	La Bastide de Serou	Ariège	Brugnara, Sébastien

Note: These are specimens of the same population.

Note: length of animal = ca 21 cm

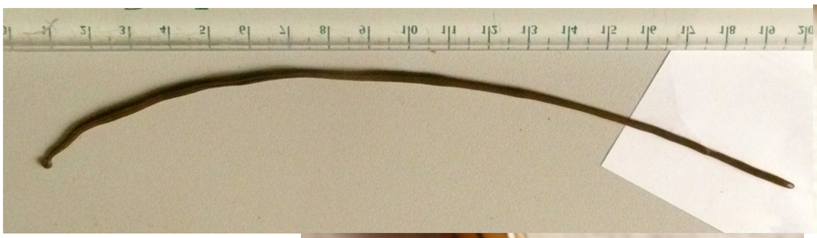
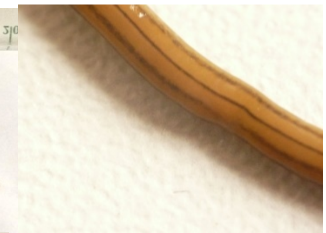
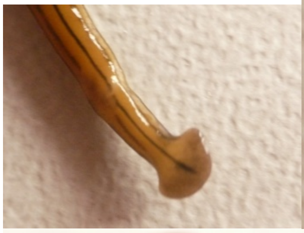
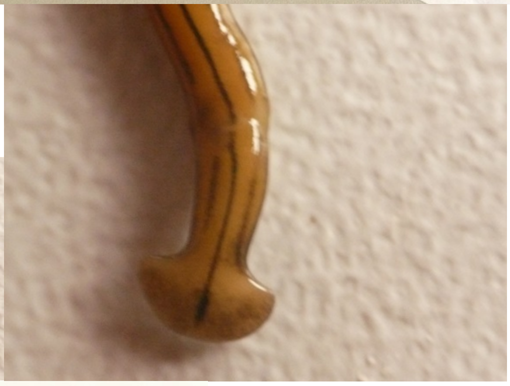
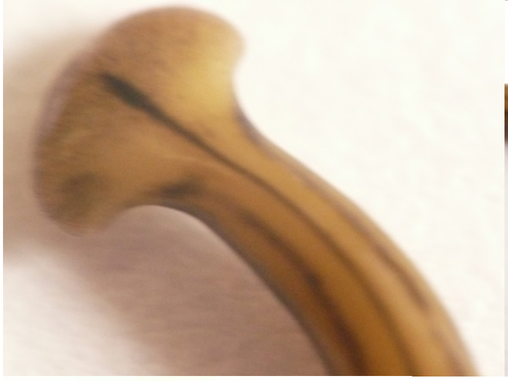
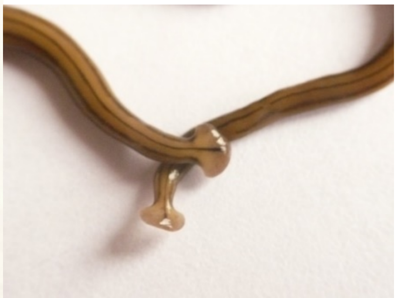
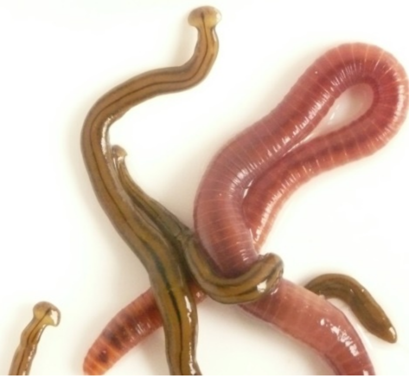

Diversibipalium multilineatum MNHN JL142

Species	MNHN	date	Locality	Department / State	Collector
DM	JL142	22/04/2014	Saubrigues	Landes	Robineau, Thiérry

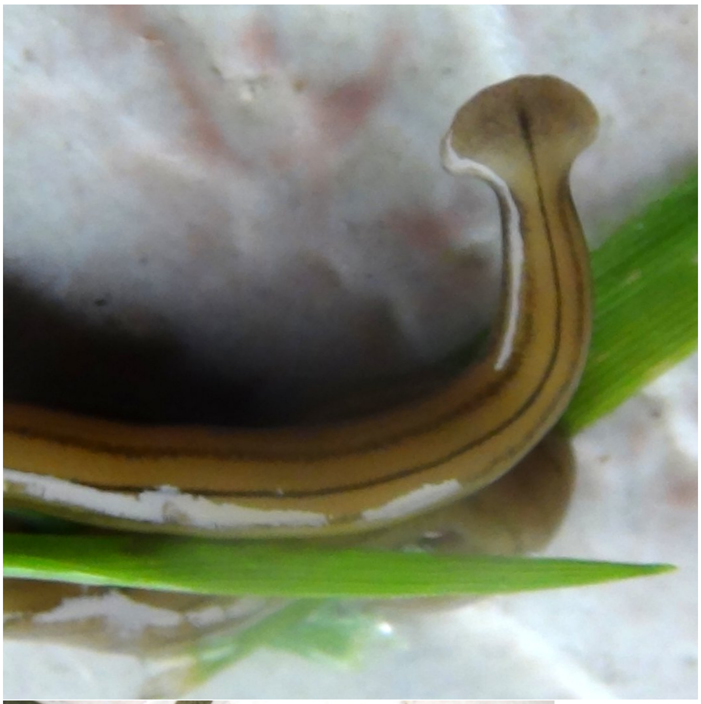

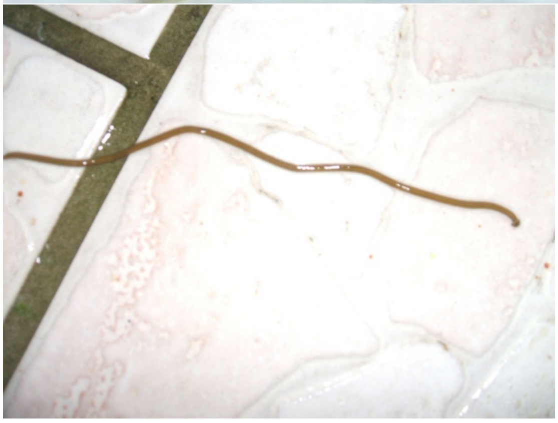

Diversibipalium multilineatum MNHN JL161

Species	MNHN	date	Locality	Department / State	Collector
DM	JL161	11/06/2015	Bellocq	Pyrénées-Atlantiques	Audiot, Marie-Claude

No photograph of live specimen.

Diversibipalium multilineatum MNHN JL208

Species	MNHN	date	Locality	Department / State	Collector
DM	JL208	11/06/2014	Bellocq	Pyrénées-Atlantiques	Audiot, Marie-Claude

No photograph of live specimen.

Diversibipalium multilineatum MNHN JL209

Species	MNHN	date	Locality	Department / State	Collector
DM	JL209	12/06/2014	Bellocq	Pyrénées-Atlantiques	Audiot, Marie-Claude

No photograph of live specimen.

Diversibipalium multilineatum MNHN JL210

Species	MNHN	date	Locality	Department / State	Collector
DM	JL210	June 2014	Bellocq	Pyrénées-Atlantiques	Audiot, Marie-Claude

No photograph of live specimen.

Diversibipalium multilineatum MNHN JL298

Species	MNHN	date	Locality	Department / State	Collector
DM	JL210	June 2014	Bellocq	Pyrénées-Atlantiques	Audiot, Marie-Claude

First row: photograph of living specimen.

Second row: photographs of preserved specimen by Jean Mariaux (Muséum de Genèse, Suisse).

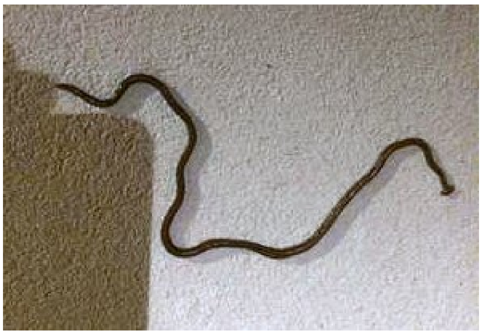

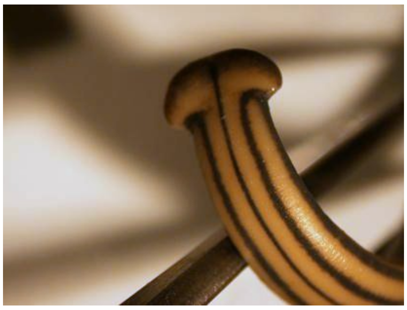

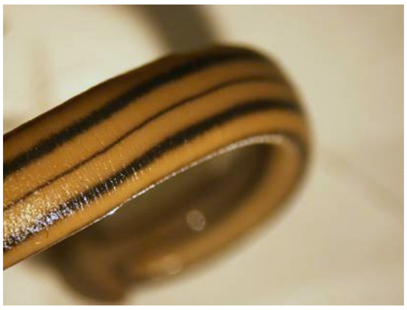

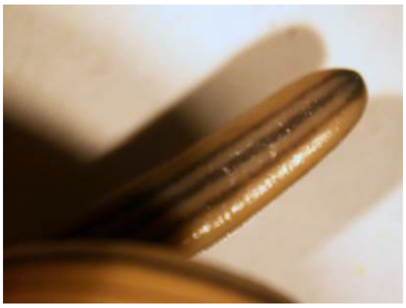

Diversibipalium sp. 'Blue' MNHN JL280

Species	MNHN	date	Locality	Department / State	Collector
DBlue	JL280	2015	Mtsamboro	Mayotte	Charles, Laurent

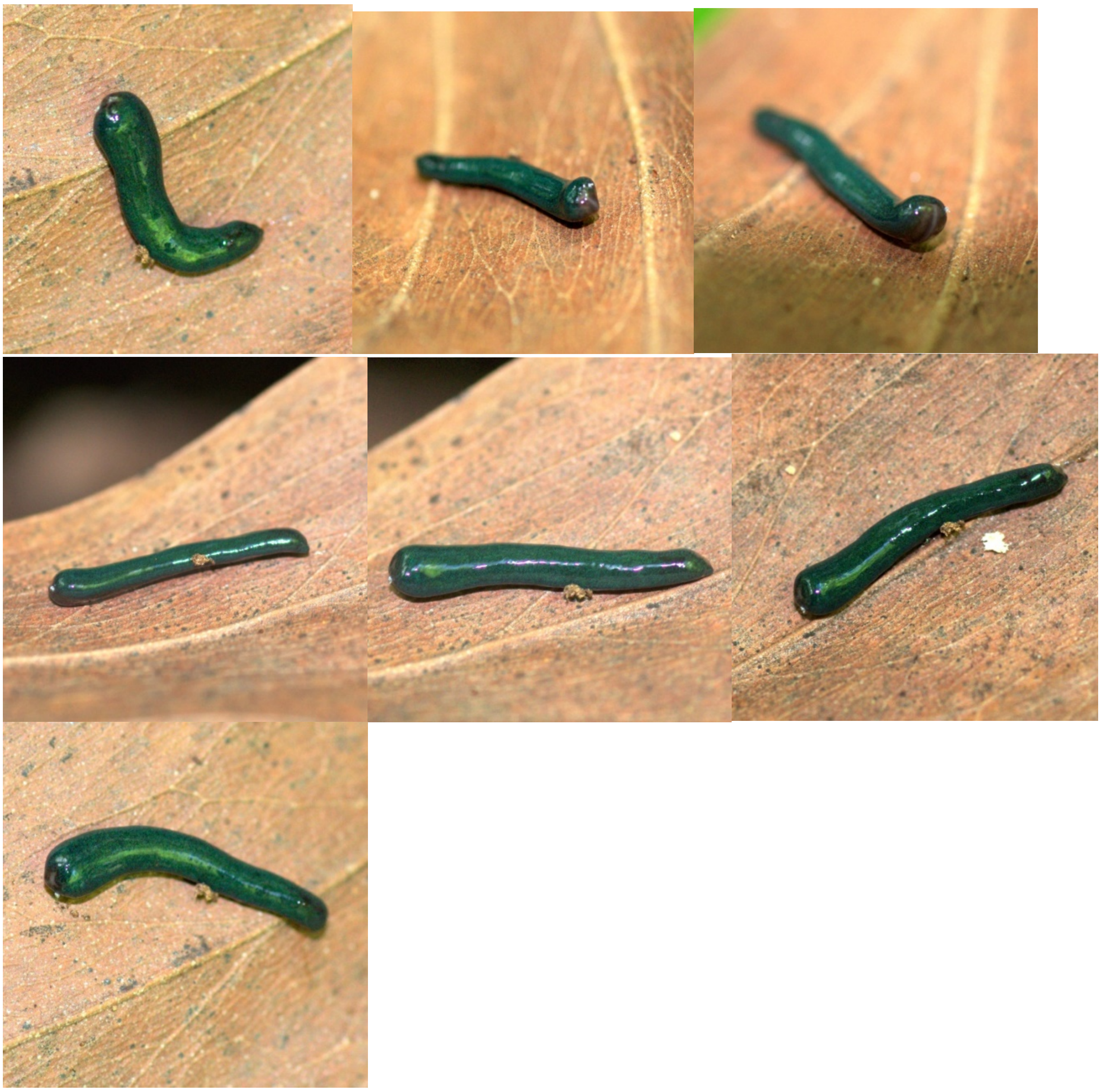

Diversibipalium sp. 'Blue' MNHN JL281

Species	MNHN	date	Locality	Department / State	Collector
DBlue	JL281	29/04/2015	Mtsamboro	Mayotte	Charles, Laurent

No photograph available.

Diversibipalium sp. ‘Blue’ MNHN JL282

Species	MNHN	date	Locality	Department / State	Collector
DBlue	JL282	30/04/2015	Ouangani	Mayotte	Charles, Laurent

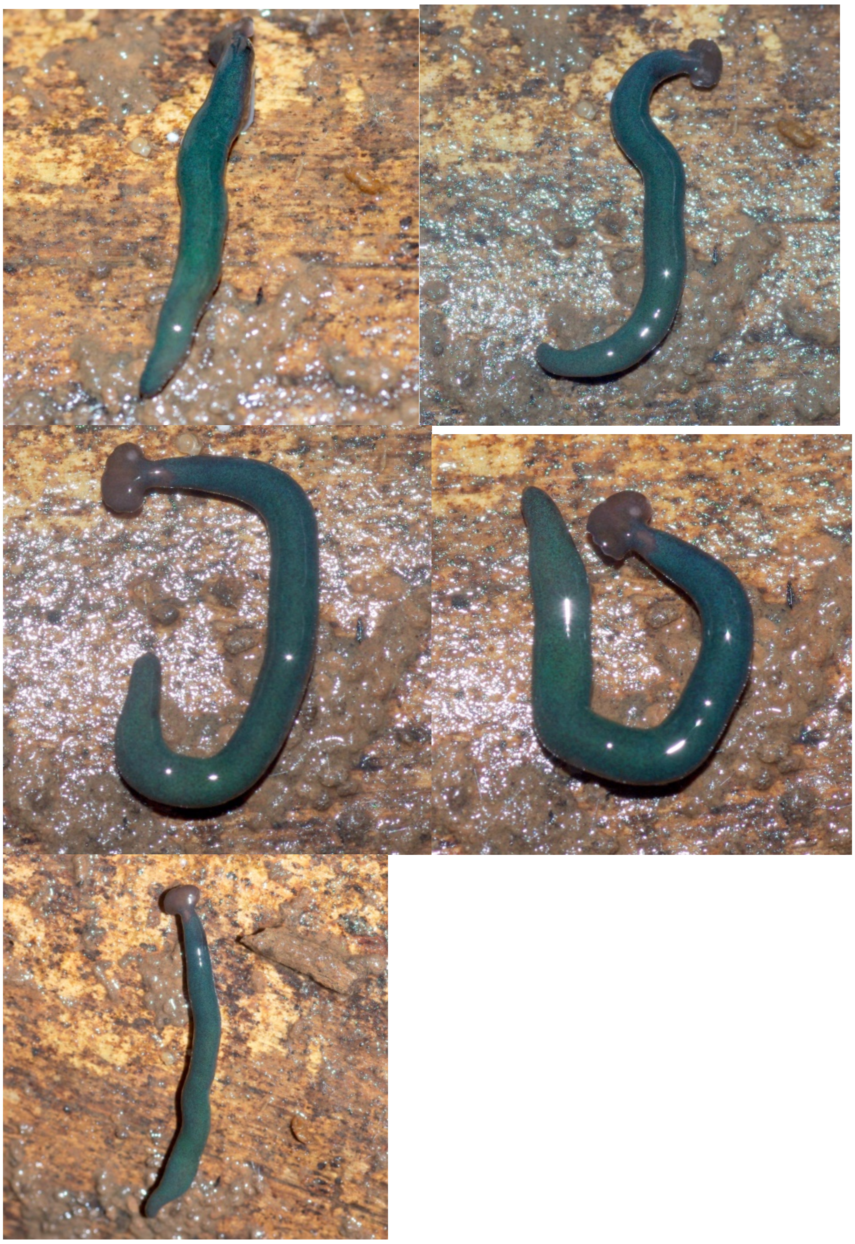

Diversibipalium sp. 'Blue' MNHN JL284

Species	MNHN	date	Locality	Department / State	Collector
DBlue	JL284	05/05/2015	Mtsamboro	Mayotte	Charles, Laurent

No photograph available.

Diversibipalium sp. 'Black MNHN JL090

Species	MNHN	date	Locality	Department / State	Collector
DBlack	JL090	12/11/2013	Saint Pée sur Nivelle	Pyrénées-Atlantiques	Consent not obtained
