# Supplementary material for: Giant worms chez moi! Hammerhead flatworms (Platyhelminthes, Geoplanidae, Bipalium spp., Diversibipalium spp.) in metropolitan France and overseas French territories
Source: PeerJ. 2018 May 22;6:e4672. doi: 10.7717/peerj.4672 (PMC5969052; doi:10.7717/peerj.4672)

Supplement 2 to article: Records from citizen science

Giant worms *chez moi!* Hammerhead flatworms (Platyhelminthes, Geoplanidae, *Bipalium* spp., *Diversibipalium* spp.) in metropolitan France and overseas French territories

Jean-Lou Justine [1*], Leigh Winsor [2], Delphine Gey [3], Pierre Gros [4] and Jessica Thévenot [5]

1 Institut Systématique Évolution Biodiversité (ISYEB), Muséum National d'Histoire Naturelle, CNRS, Sorbonne Université, EPHE, 57 rue Cuvier, CP 51, 75005 Paris, France

2 College of Science and Engineering, James Cook University, Townsville, Australia

3 Service de Systématique Moléculaire, Muséum National d’Histoire Naturelle, Paris, France

4 Amateur Naturalist, Cagnes-sur-Mer, France

5 Coordination technique et scientifique de la stratégie nationale relative aux espèces exotiques envahissantes, UMS Patrinat, Muséum National d’Histoire Naturelle, Sorbonne Universités, Paris, France

PeerJ 6 : e4672 (2018)

DOI : 10.7717/peerj.4672

Supplement 2 to article: Records from citizen science

Reports of sighting of land planarians were received from citizens, mainly by email, sometimes by telephone. Photographs and details about locality were solicited, and only reports including this information were considered. Wrong records (slugs, myriapods, earthworms, leeches, caterpillars, nematomorphs, and nemerteans) were eliminated. Information collected from citizen science allowed monitoring of several land planarians (Justine et al. 2014a). Photographs were studied, and species were identified whenever possible. Only information relative to bipaliines is reported in this paper). Sometimes citizens provided records dating from before the survey, such as an amateur movie taken in 1999. Most citizens provided an authorisation to use the photographs at the time of the initial contact by email, and most photographs were posted to the twitter account @Plathelminthe4 https://twitter.com/Plathelminthe4.

When we prepared this paper for publication, we sought authorization to use the photographs and to publish them under a Creative Commons Licence; only one of the citizens refused to provide the authorization, but some of them did not respond, probably simply because they changed their emails or did not check them. In these cases, we provide the scientific information about the presence of species, but we do not include the photograph of the worm or the name of the citizen in the paper; these records are marked “consent not obtained”.

Bipalium kewense: Records

Records of *Bipalium kewense* identified from photographs.

#	Date	Locality	Department / State	Country - Continent	Origin of data
**K01**	20/08/2017	Bora Bora	French Polynesia	French Polynesia - Oceania	Gerlach, Justin
**K02**	13/10/2010	Basse-Terre	Guadeloupe	Guadeloupe - C. America	Guezennec, Pierre et Claudine
**K03**	22/01/2014	Unknown	Guadeloupe	Guadeloupe - C. America	Consent not obtained
**K04**	14/01/2007	Petit-Bourg	Guadeloupe	Guadeloupe - C. America	Lurel, Félix
**K05**	19/02/2015	La Trinité	Martinique	Martinique - C. America	Delannoye, Régis
**K06**	19/04/2016	Saint Joseph	Martinique	Martinique - C. America	Andrebe, Silvio
**K07**	25/08/2017	Plaine des Cafres	La Réunion	La Réunion - Africa	Pronier, Pascal
**K08**	03/11/2013	Cagnes-sur-Mer	Alpes-Maritimes	Met. France - Europe	Gros, Pierre
**K09**	19/01/2014	Cagnes-sur-Mer	Alpes-Maritimes	Met. France - Europe	Gros, Pierre
**K10**	05/11/2014	Cagnes-sur-Mer	Alpes-Maritimes	Met. France - Europe	Gros, Pierre
**K11**	16/10/2013	Beaulieu-sur-Mer	Alpes-Maritimes	Met. France - Europe	Pelcer, Jean-Paul
**K12**	21/07/2014	Nice	Alpes-Maritimes	Met. France - Europe	Gerriet, Olivier *
**K13**	15/10/2014	Appietto	Corse-Sud (Corsica)	Met. France - Europe	Consent not obtained
**K14**	17/10/2013	Pietrosella	Corse-Sud (Corsica)	Met. France - Europe	Senee, Patrick
**K15**	23/08/2014	Arcachon	Gironde	Met. France - Europe	Consent not obtained
**K16**	21/11/2002	Saint-Jean-de-Vedas	Hérault	Met. France - Europe	Peaucellier, Gérard
**K17**	27/10/2014	Biscarosse	Landes	Met. France - Europe	Consent not obtained
**K18**	27/09/2008	Hagetmau	Landes	Met. France - Europe	Jeannotin, Josette
**K19**	22/09/2016	Nantes	Loire-Atlantique	Met. France - Europe	Consent not obtained
**K20**	16/10/2014	Grimaud	Var	Met. France - Europe	Bernez, Alain
**K21**	01/08/2014	Toulon	Var	Met. France - Europe	Consent not obtained
**K22**	29/07/2014	Sens (Hothouse)	Yonne	Met. France - Europe	Burel, Jonathan **
**K23**	23/10/2017	Peyrouse	Hautes-Pyrénées	Met. France - Europe	Tremosa, Clémence
**K24**	17/12/2014	Arthez de Béarn	Pyrénées-Atlantiques	Met. France - Europe	Sillard, Dominique
**K25**	17/09/2017	Billère	Pyrénées-Atlantiques	Met. France - Europe	Rolland, Geneviève
**K26**	28/01/2018	Billère	Pyrénées-Atlantiques	Met. France - Europe	Rolland, Geneviève
**K27**	20/09/2014	Bayonne	Pyrénées-Atlantiques	Met. France - Europe	Bonnefous, François
**K28**	18/08/2014	Hasparren	Pyrénées-Atlantiques	Met. France - Europe	Voise, Mireille
**K29**	22/04/2016	Jurançon (near)	Pyrénées-Atlantiques	Met. France - Europe	Pauchet, Marjolaine
**K30**	29/04/2016	Nay	Pyrénées-Atlantiques	Met. France - Europe	Lamaille, Corinne
**K31**	28/09/2014	Orthez	Pyrénées-Atlantiques	Met. France - Europe	Rougeux, Christian
**K32**	22/08/2016	Saint Jean de Luz	Pyrénées-Atlantiques	Met. France - Europe	Centelles, Ruben
**K33**	01/01/1999	Urcuit	Pyrénées-Atlantiques	Met. France - Europe	Esposito, Mario
**K34**	14/09/2014	Urt	Pyrénées-Atlantiques	Met. France - Europe	Chanderot, Vincent
**K35**	12/08/2017	Ustaritz	Pyrénées-Atlantiques	Met. France - Europe	Lescourret, Monique & Bernard
**K36**	14/09/2014	Villefranque	Pyrénées-Atlantiques	Met. France - Europe	Consent not obtained

* Muséum d'Histoire Naturelle, Nice, France; **, FREDON.

Bipalium kewense K01

#	Date	Locality	Department / State	Country - Continent	Origin of data
**K01**	20/08/2017	Bora Bora	French Polynesia	French Polynesia - Oceania	Gerlach, Justin

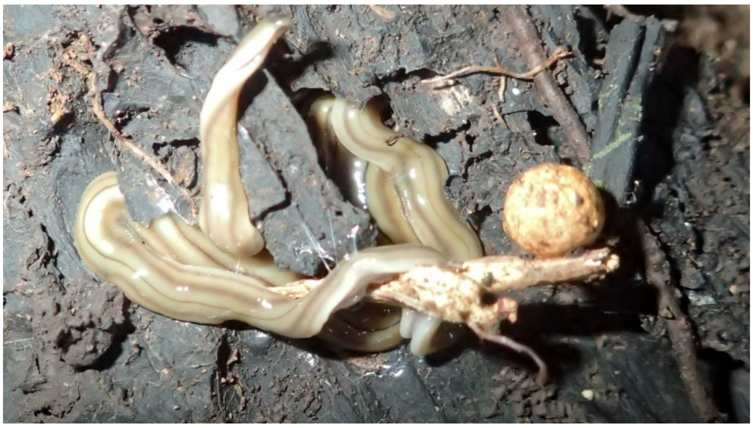

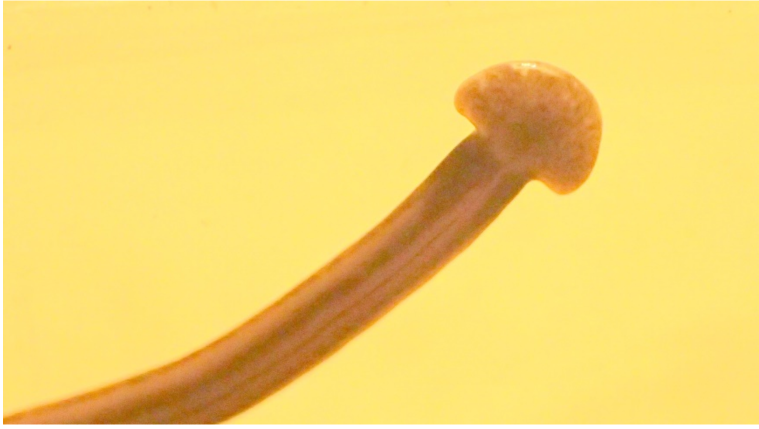

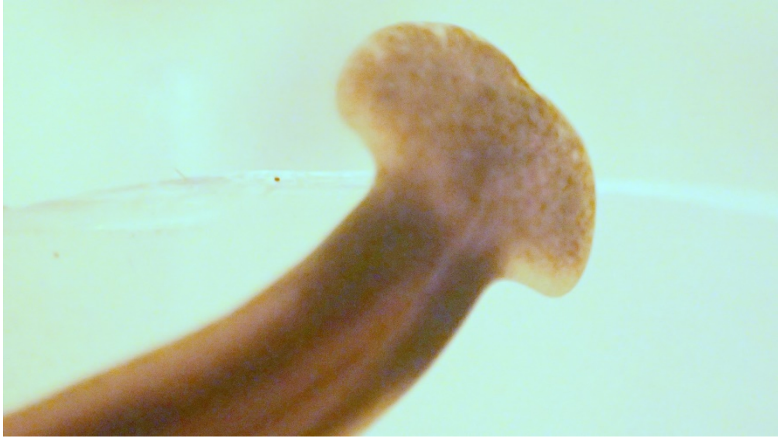

Bipalium kewense K02

#	Date	Locality	Department / State	Country - Continent	Origin of data
**K02**	13/10/2010	Basse-Terre	Guadeloupe	Guadeloupe - C. America	Guezennec, Pierre et Claudine

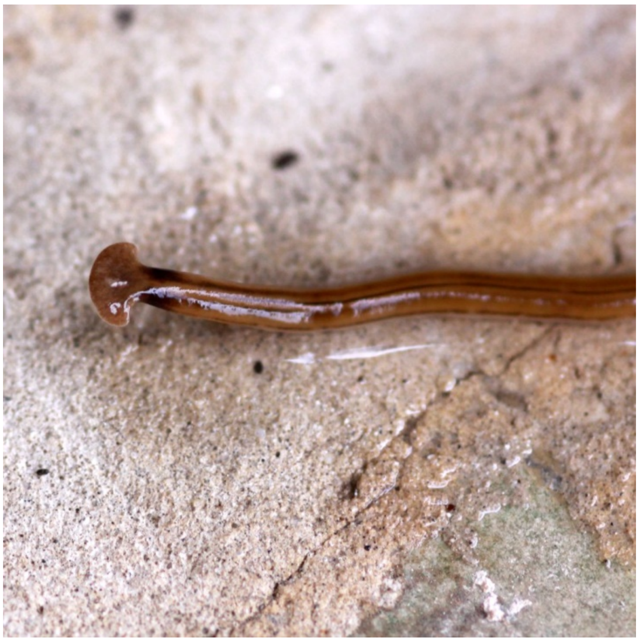

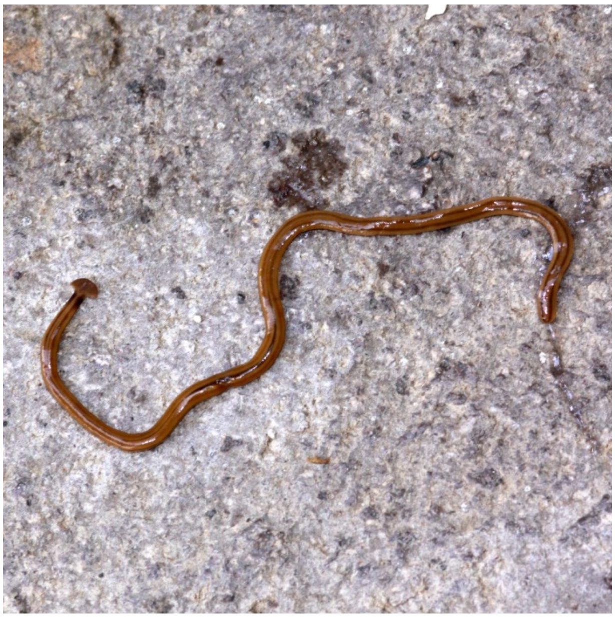

Bipalium kewense K03

#	Date	Locality	Department / State	Country - Continent	Origin of data
**K03**	22/01/2014	Unknown	Guadeloupe	Guadeloupe - C. America	Consent not obtained

Bipalium kewense K04

#	Date	Locality	Department / State	Country - Continent	Origin of data
**K04**	14/01/2007	Petit-Bourg	Guadeloupe	Guadeloupe - C. America	Lurel, Félix

Note: length of animal = ca. 13 cm

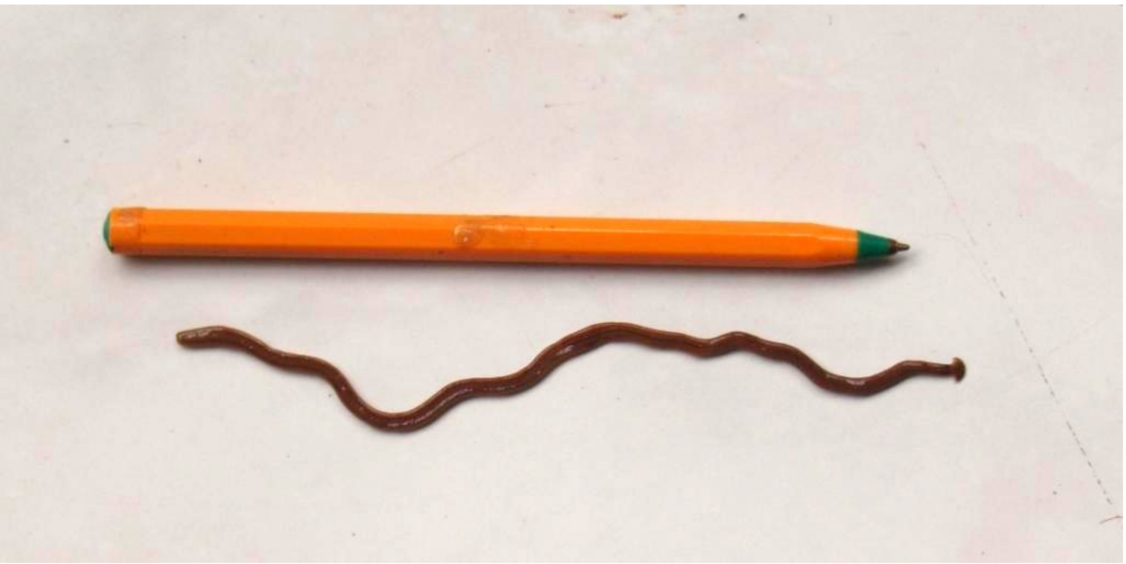

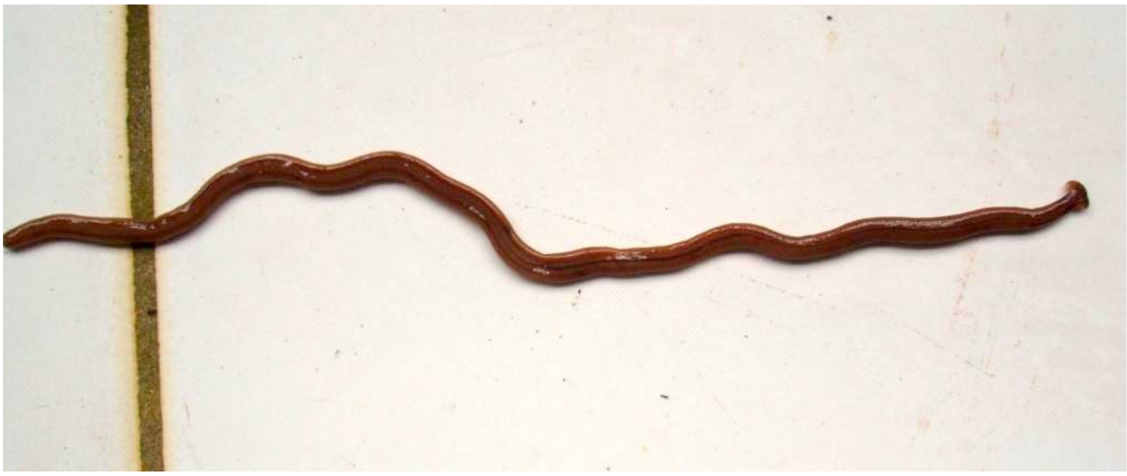

Bipalium kewense K05

#	Date	Locality	Department / State	Country - Continent	Origin of data
**K05**	19/02/2015	La Trinité	Martinique	Martinique - C. America	Delannoye, Régis

Note: length of animal = 20 cm

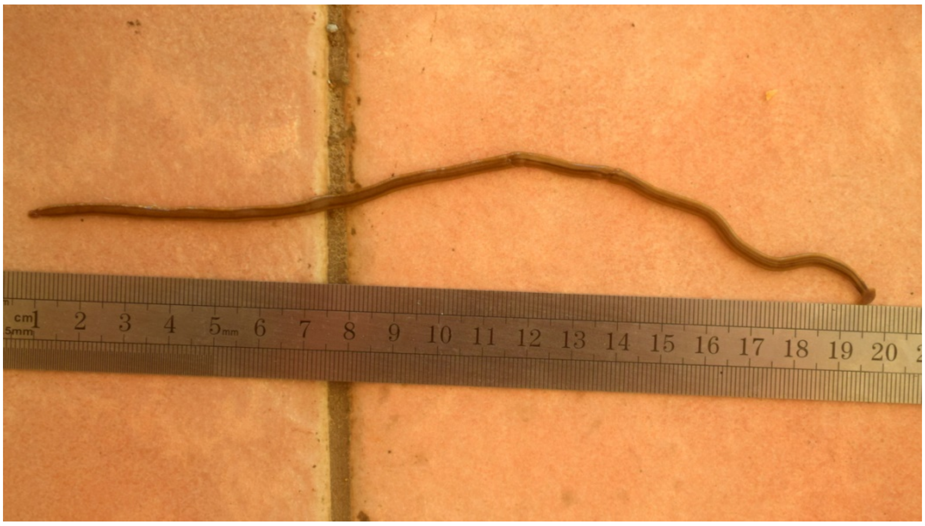

Bipalium kewense K06

#	Date	Locality	Department / State	Country - Continent	Origin of data
**K06**	19/04/2016	Saint Joseph	Martinique	Martinique - C. America	Andrebe, Silvio

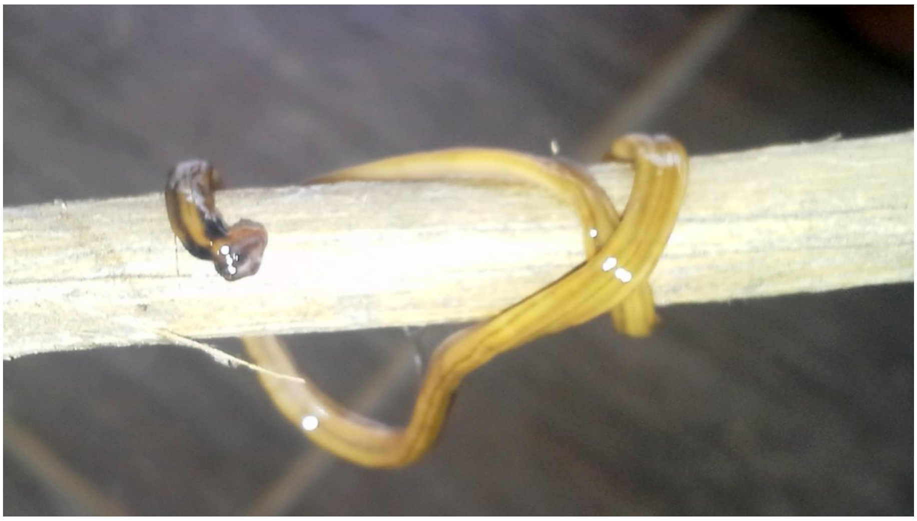

Bipalium kewense K07

#	Date	Locality	Department / State	Country - Continent	Origin of data
**K07**	25/08/2017	Plaine des Cafres	La Réunion	La Réunion - Africa	Pronier, Pascal

Note: length of animal = 10 cm

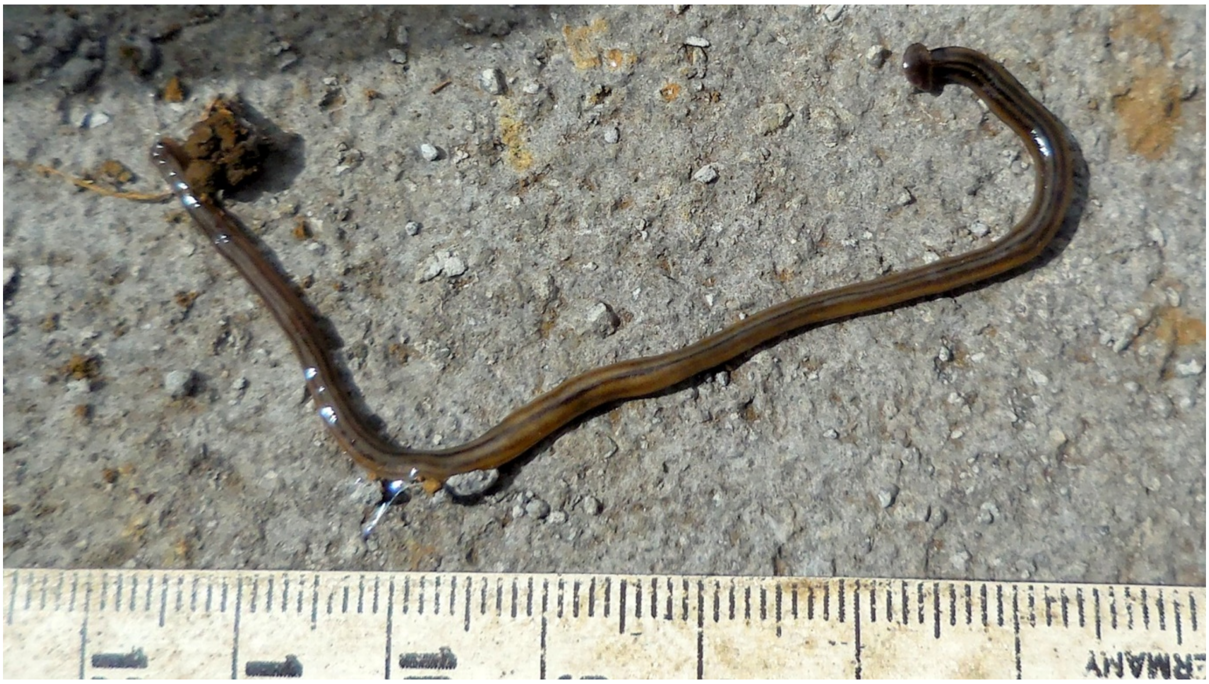

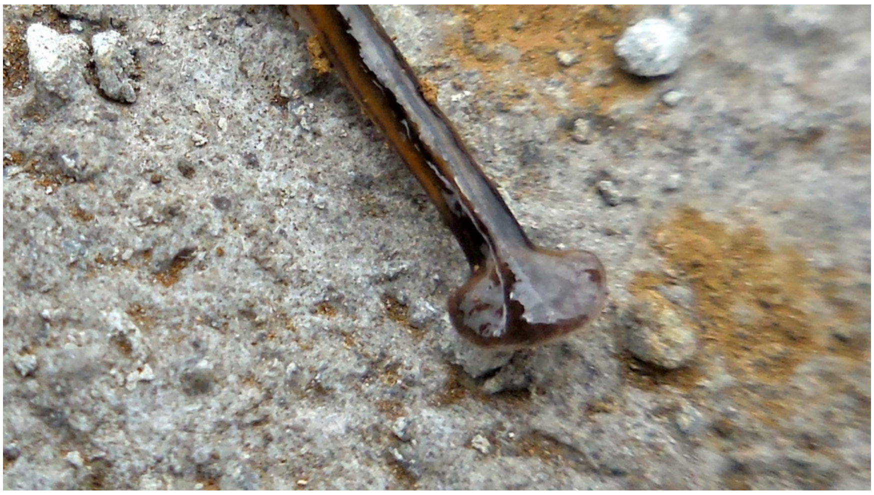

Bipalium kewense K08-K10

#	Date	Locality	Department / State	Country - Continent	Origin of data
**K08**	03/11/2013	Cagnes-sur-Mer	Alpes-Maritimes	Met. France - Europe	Gros, Pierre
**K09**	19/01/2014	Cagnes-sur-Mer	Alpes-Maritimes	Met. France - Europe	Gros, Pierre
**K10**	05/11/2014	Cagnes-sur-Mer	Alpes-Maritimes	Met. France - Europe	Gros, Pierre

The records are illustrated with figures in the text.

Bipalium kewense K11

#	Date	Locality	Department / State	Country - Continent	Origin of data
**K11**	16/10/2013	Beaulieu-sur-Mer	Alpes-Maritimes	Met. France - Europe	Pelcer, Jean-Paul

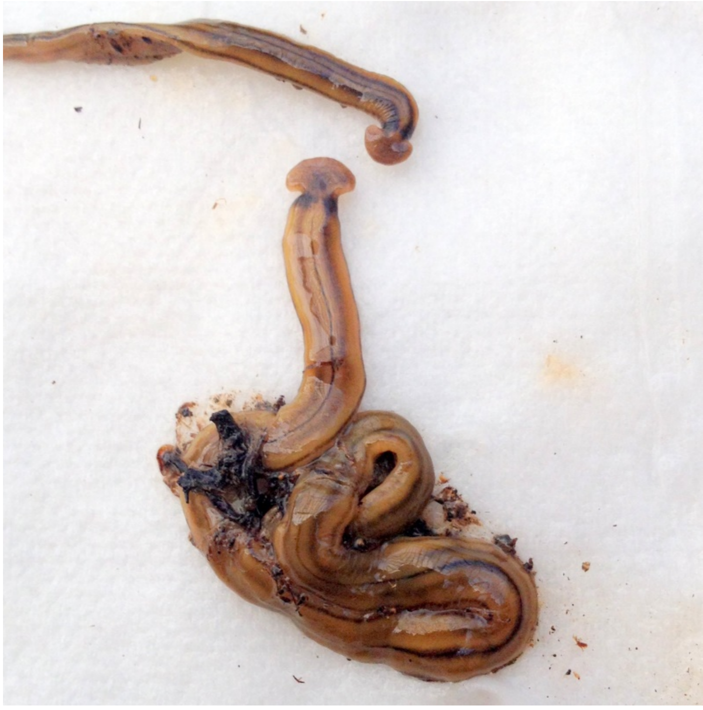

Bipalium kewense K12

#	Date	Locality	Department / State	Country - Continent	Origin of data
K12	21/07/2014	Nice	Alpes-Maritimes	Met. France - Europe	Gerriet, Olivier *

Note : several individuals in public gardens in Nice.

Specimens kept in Muséum d'Histoire Naturelle, Nice, France.

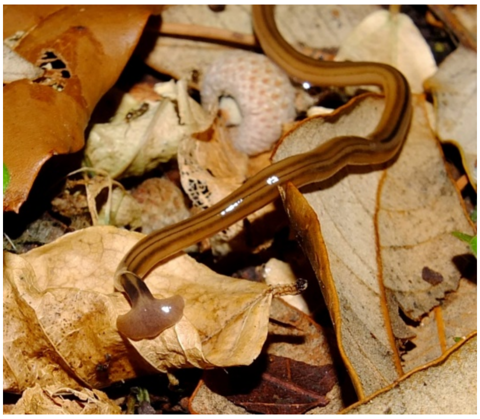

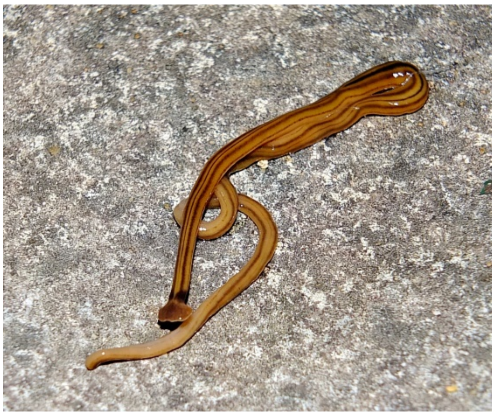

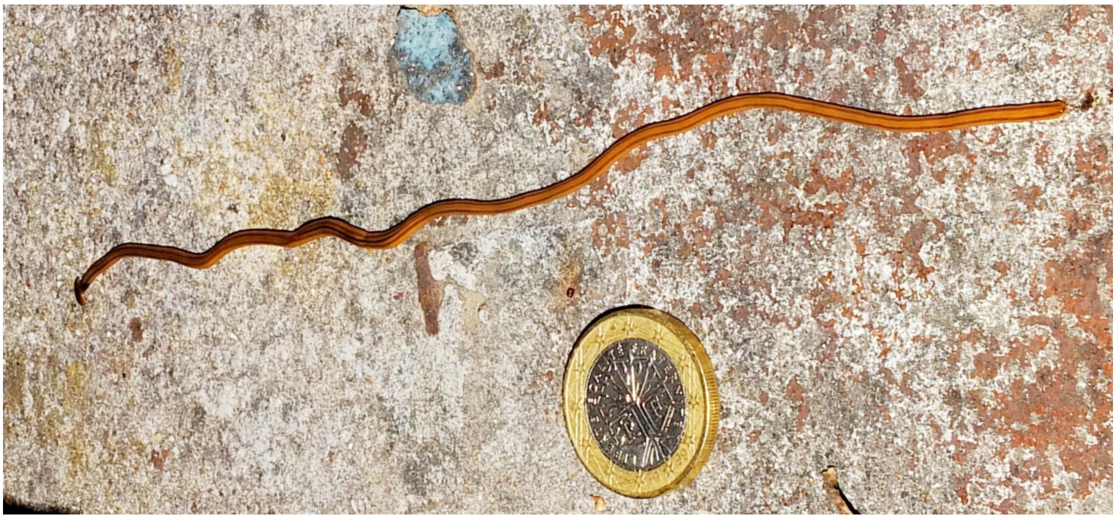

Note: the diameter of the 1€ coin is 23.25 mm

Bipalium kewense K13

#	Date	Locality	Department / State	Country - Continent	Origin of data
**K13**	15/10/2014	Appietto	Corse-Sud (Corsica)	Met. France - Europe	Consent not obtained

Bipalium kewense K14

#	Date	Locality	Department / State	Country - Continent	Origin of data
**K14**	17/10/2013	Pietrosella	Corse-Sud (Corsica)	Met. France - Europe	Senee, Patrick

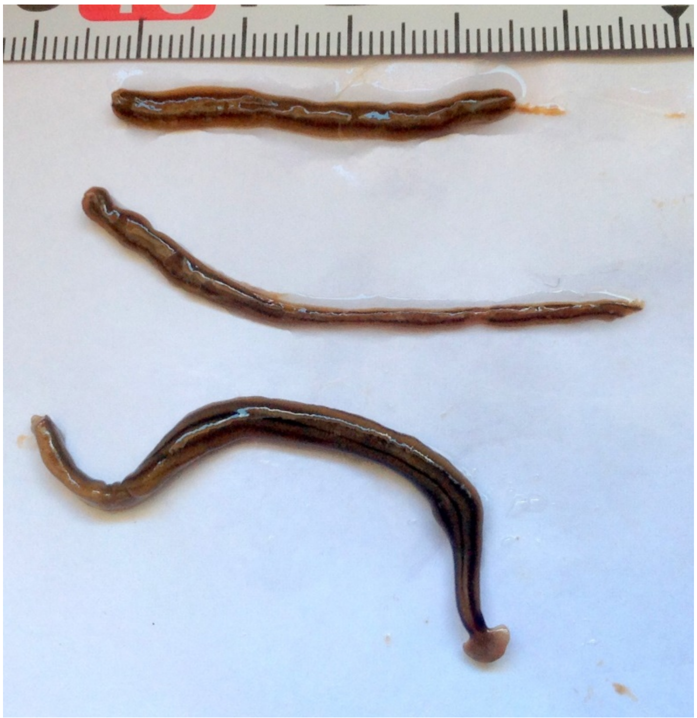

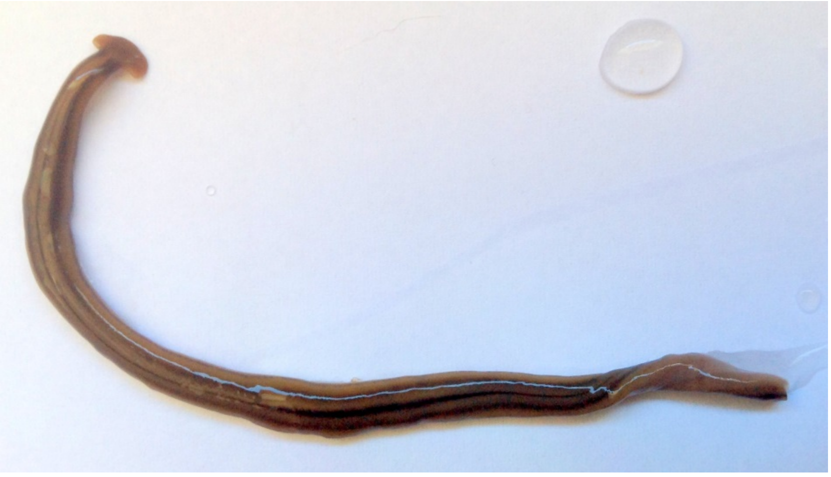

Bipalium kewense K15

#	Date	Locality	Department / State	Country - Continent	Origin of data
**K15**	23/08/2014	Arcachon	Gironde	Met. France - Europe	Consent not obtained

Bipalium kewense K16

#	Date	Locality	Department / State	Country - Continent	Origin of data
**K16**	21/11/2002	Saint-Jean-de-Vedas	Hérault	Met. France - Europe	Peaucellier, Gérard

Note: early record (2002).

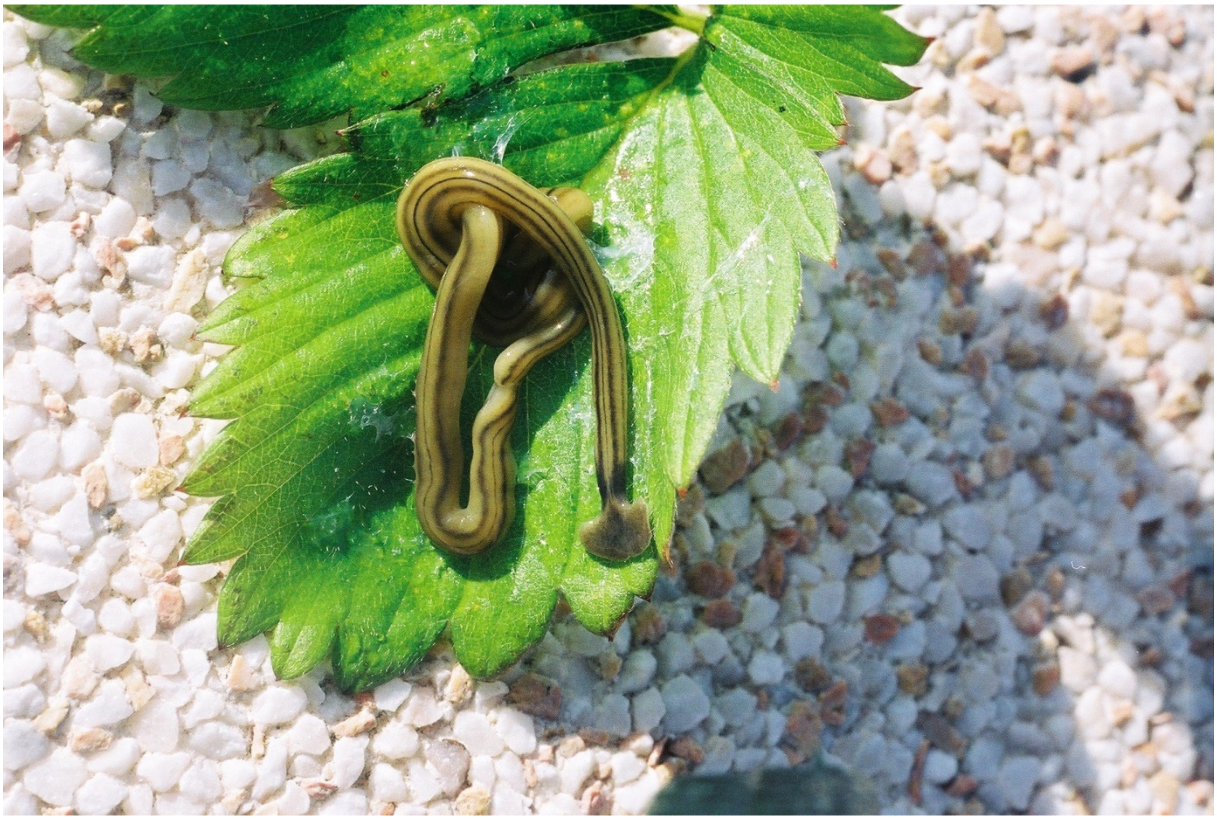

Bipalium kewense K17

#	Date	Locality	Department / State	Country - Continent	Origin of data
**K17**	27/10/2014	Biscarosse	Landes	Met. France - Europe	Consent not obtained

Bipalium kewense K18

#	Date	Locality	Department / State	Country - Continent	Origin of data
**K18**	27/09/2008	Hagetmau	Landes	Met. France - Europe	Jeannotin, Josette

Note: early record (2008).

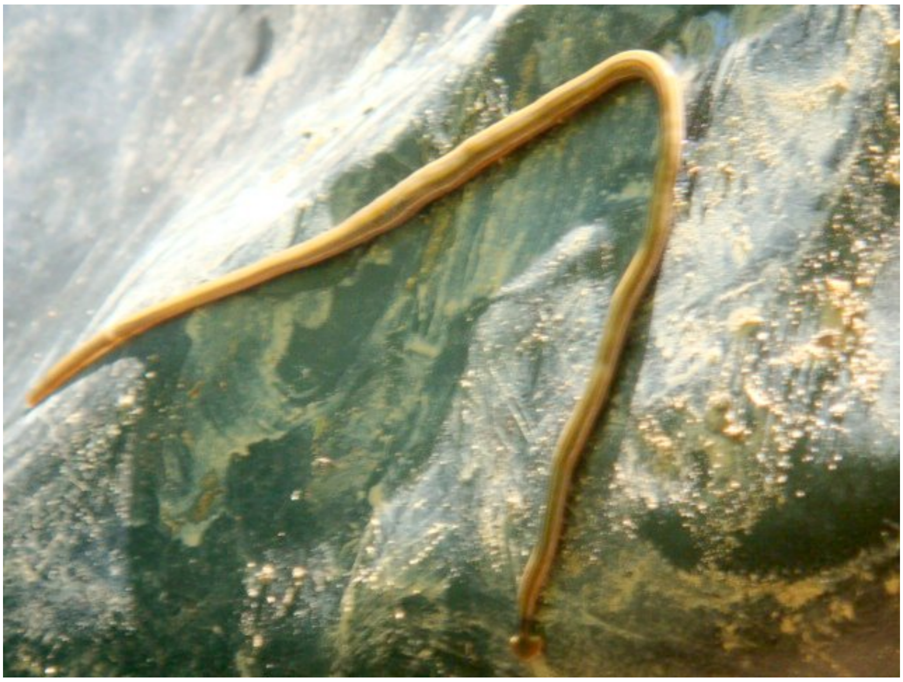

Bipalium kewense K19

#	Date	Locality	Department / State	Country - Continent	Origin of data
**K19**	22/09/2016	Nantes	Loire-Atlantique	Met. France - Europe	Consent not obtained

Bipalium kewense K20

#	Date	Locality	Department / State	Country - Continent	Origin of data
**K20**	16/10/2014	Grimaud	Var	Met. France - Europe	Bernez, Alain

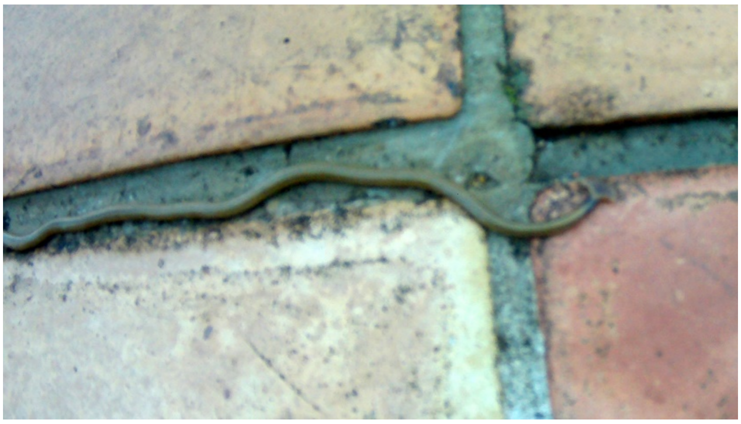

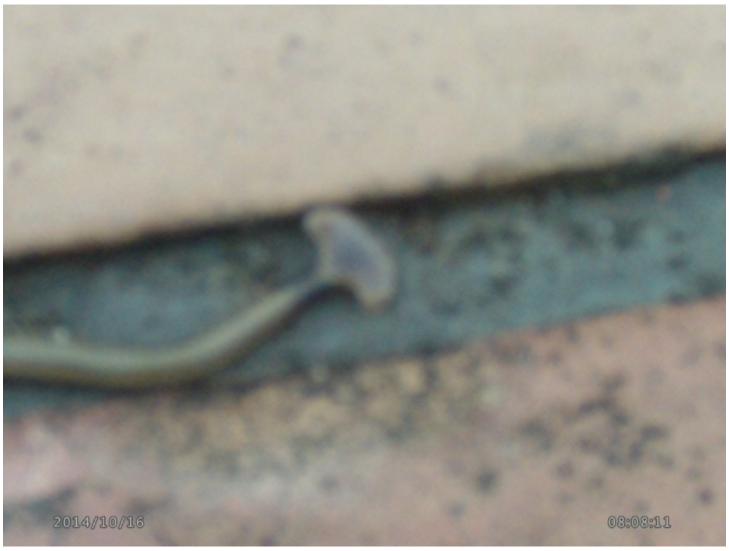

Bipalium kewense K21

#	Date	Locality	Department / State	Country - Continent	Origin of data
**K21**	01/08/2014	Toulon	Var	Met. France - Europe	Consent not obtained

Bipalium kewense K22

#	Date	Locality	Department / State	Country - Continent	Origin of data
**K22**	29/07/2014	Sens (Hothouse)	Yonne	Met. France - Europe	Burel, Jonathan **

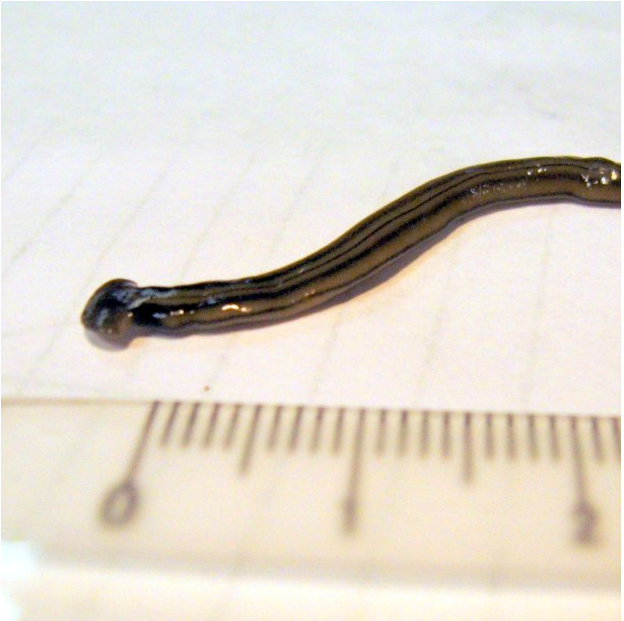

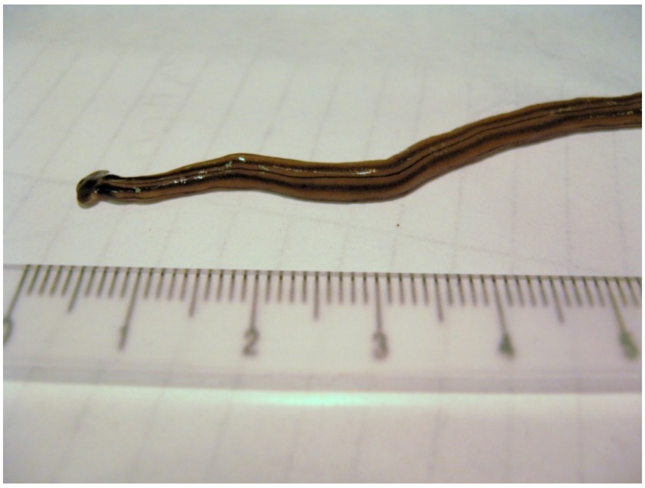

Bipalium kewense K23

#	Date	Locality	Department / State	Country - Continent	Origin of data
K23	23/10/2017	Peyrouse	Haute-Pyrénées	Met. France - Europe	Tremosa, Clémence

Note: numerous specimens, collected the same day.

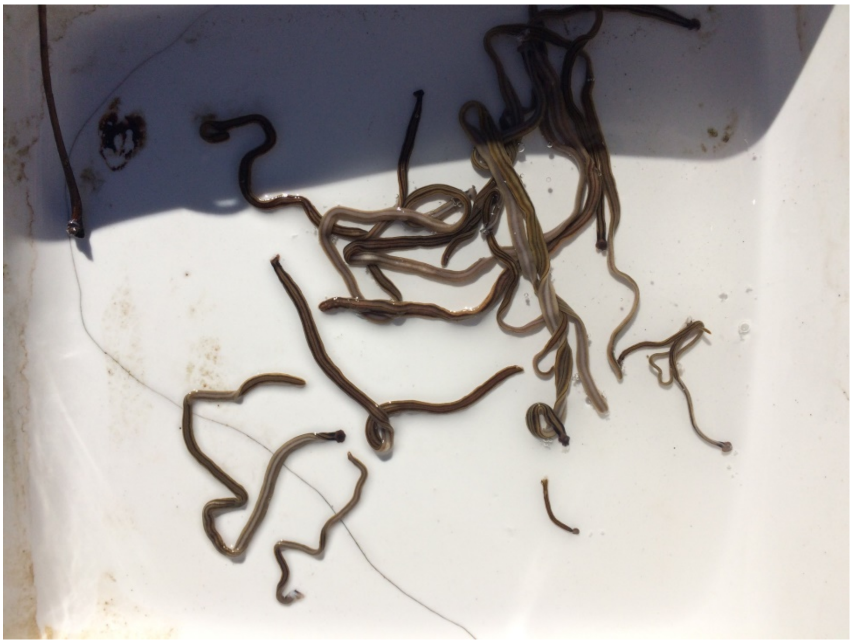

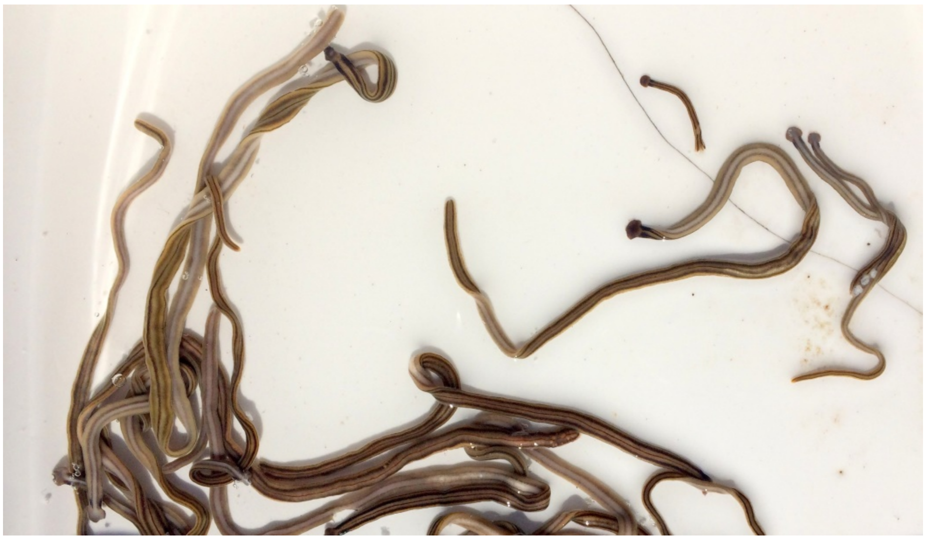

Bipalium kewense K24

#	Date	Locality	Department / State	Country - Continent	Origin of data
**K24**	17/12/2014	Arthez de Béarn	Pyrénées-Atlantiques	Met. France - Europe	Sillard, Dominique

Note: length of animal = 20 cm

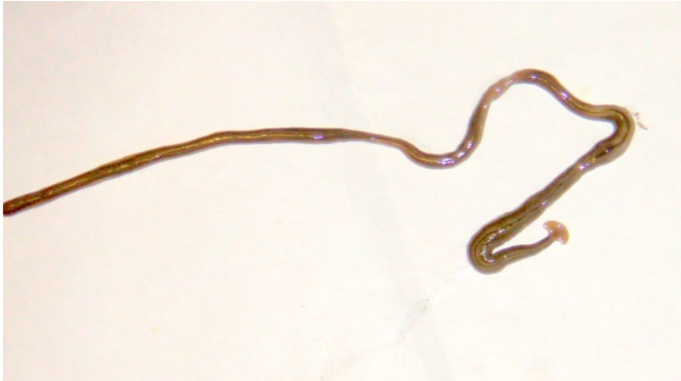

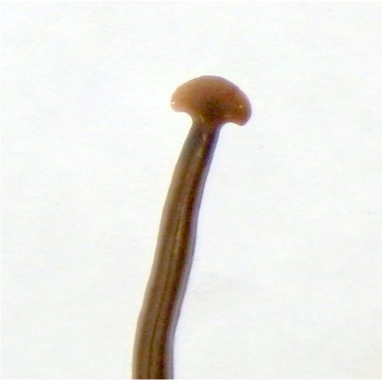

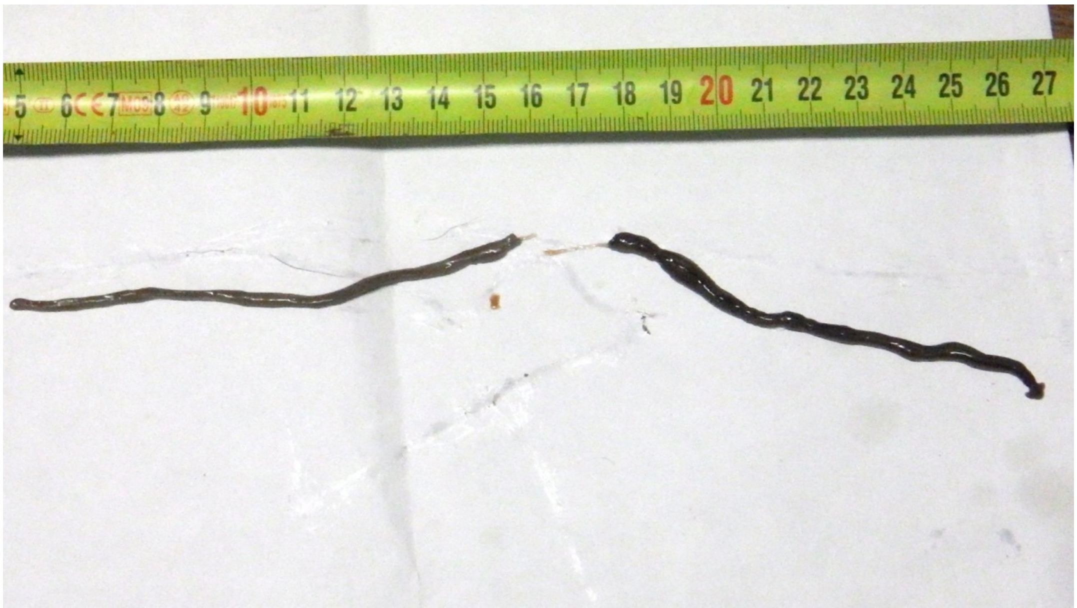

Bipalium kewense K25-K26

#	Date	Locality	Department / State	Country - Continent	Origin of data
**K25**	17/09/2017	Billère	Pyrénées-Atlantiques	Met. France - Europe	Rolland-Martinez, Geneviève

Note: length of animal = 27 cm

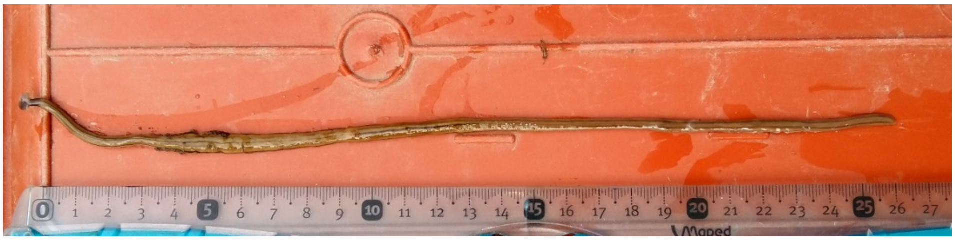

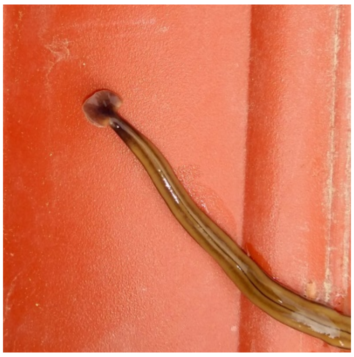

#	Date	Locality	Department / State	Country - Continent	Origin of data
**K26**	28/01/2018	Billère	Pyrénées-Atlantiques	Met. France - Europe	Rolland-Martinez, Geneviève

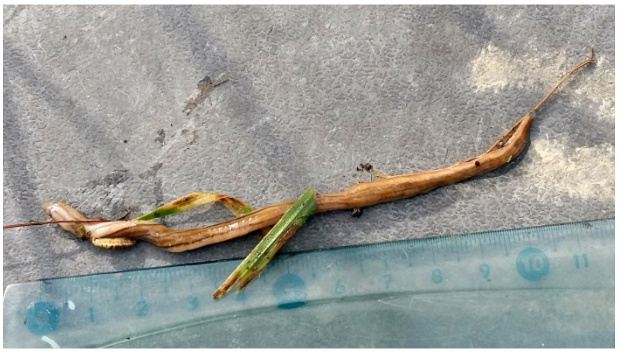

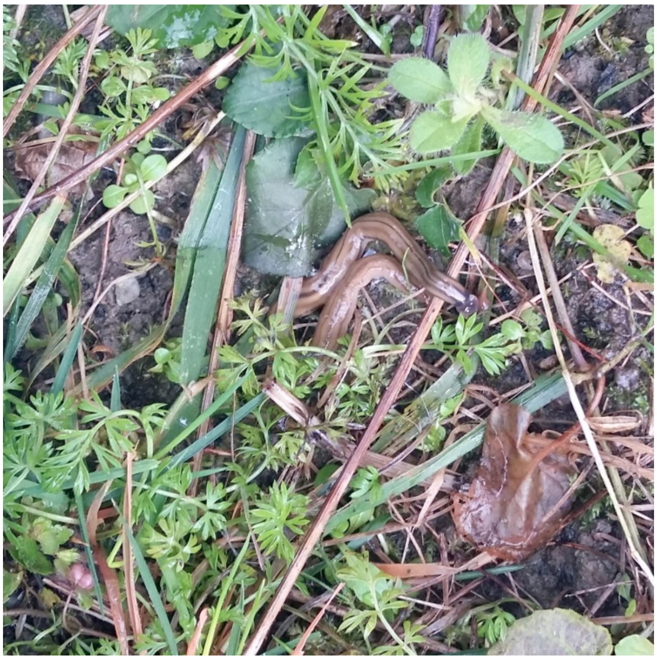

Bipalium kewense K27

#	Date	Locality	Department / State	Country - Continent	Origin of data
**K27**	20/09/2014	Bayonne	Pyrénées-Atlantiques	Met. France - Europe	Bonnefous, François

Note: Also *Parakontikia ventrolineata*.

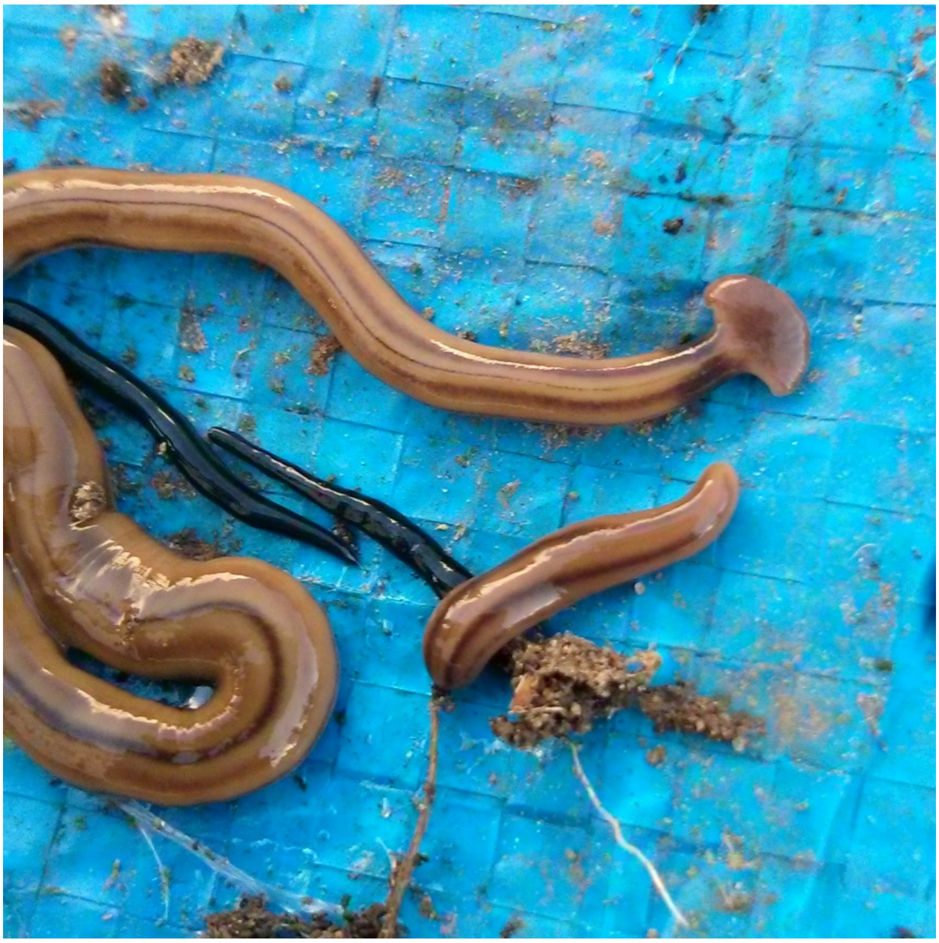

Bipalium kewense K28

#	Date	Locality	Department / State	Country - Continent	Origin of data
**K28**	18/08/2014	Hasparren	Pyrénées-Atlantiques	Met. France - Europe	Voise, Mireille

Note: length of animal = 15 cm

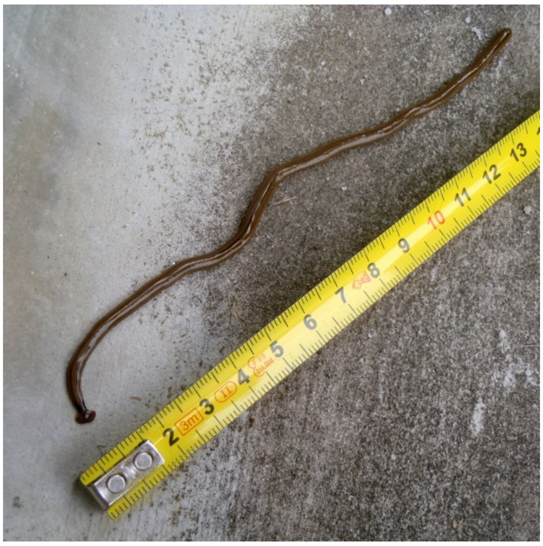

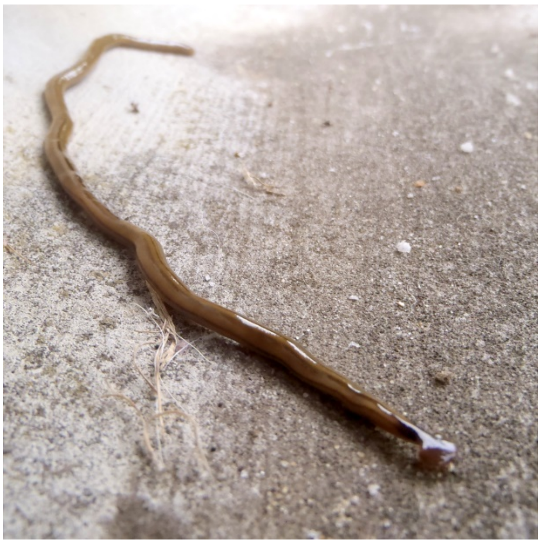

Bipalium kewense K29

#	Date	Locality	Department / State	Country - Continent	Origin of data
**K29**	22/04/2016	Jurançon (near)	Pyrénées-Atlantiques	Met. France - Europe	Pauchet, Marjolaine

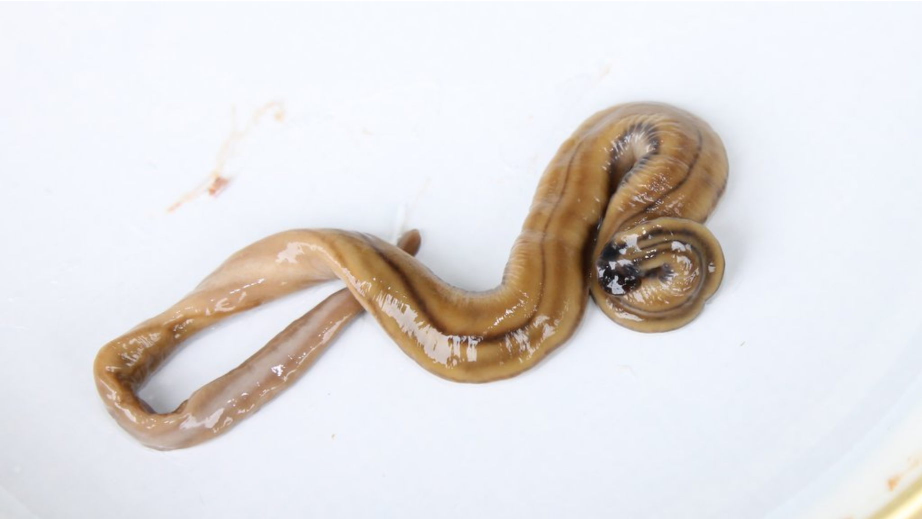

Bipalium kewense K30

#	Date	Locality	Department / State	Country - Continent	Origin of data
K30	29/04/2016	Nay	Pyrénées-Atlantiques	Met. France - Europe	Lamaille, Corinne

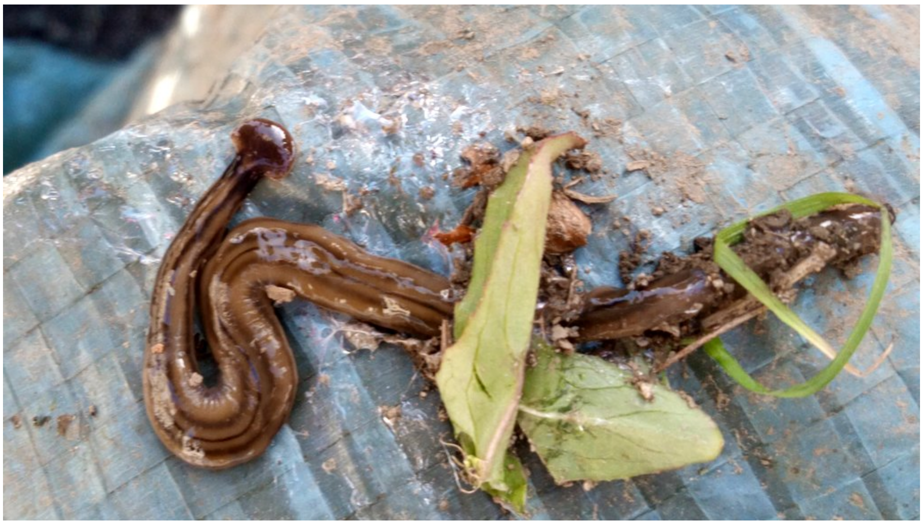

Bipalium kewense K31

#	Date	Locality	Department / State	Country - Continent	Origin of data
K31	28/09/2014	Orthez	Pyrénées-Atlantiques	Met. France - Europe	Rougeux, Christian

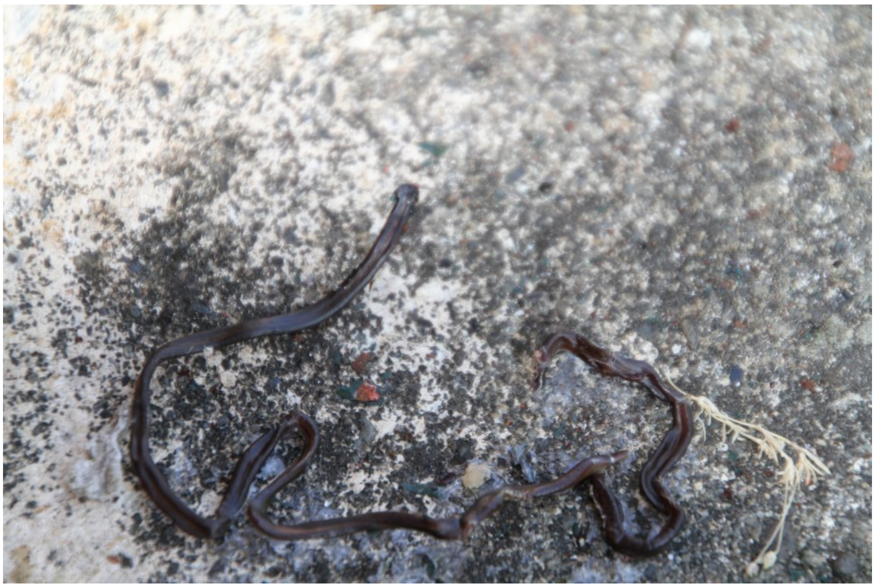

Bipalium kewense K32

#	Date	Locality	Department / State	Country - Continent	Origin of data
**K32**	22/08/2016	Saint Jean de Luz	Pyrénées-Atlantiques	Met. France - Europe	Centelles, Ruben

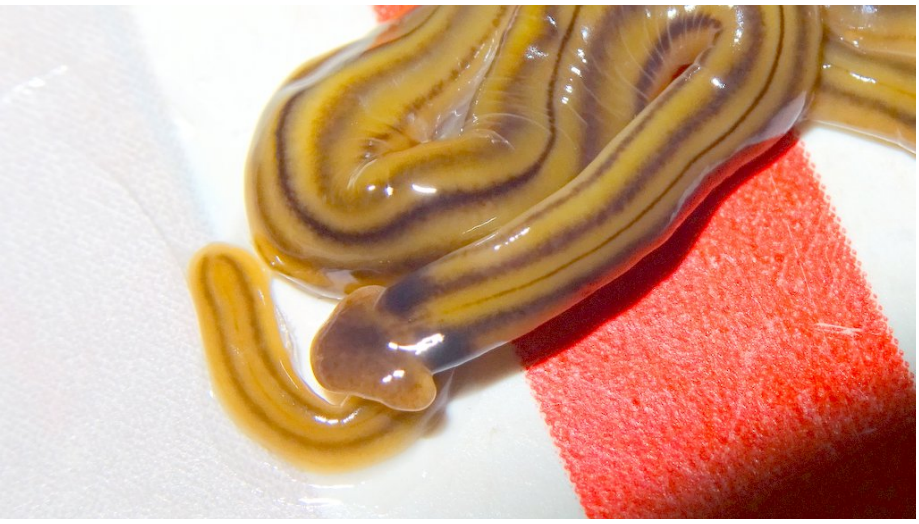

Bipalium kewense K33

#	Date	Locality	Department / State	Country - Continent	Origin of data
K33	01/01/1999	Urcuit	Pyrénées-Atlantiques	Met. France - Europe	Esposito, Mario

Note: images extracted from movie. Early record (1999).

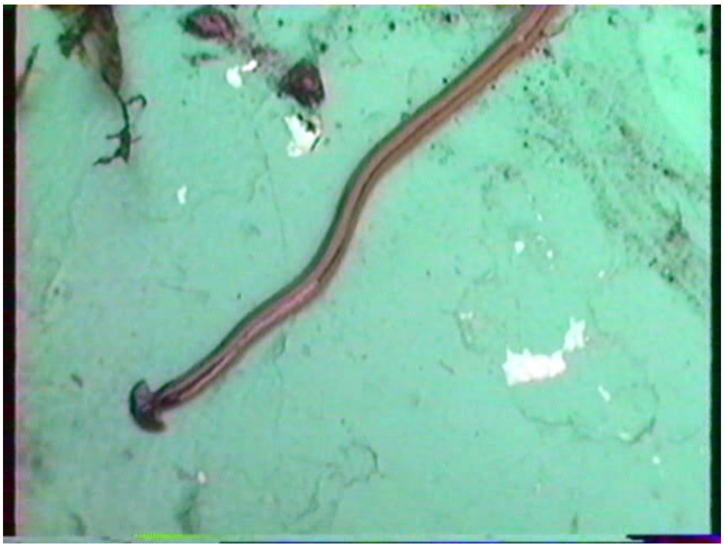

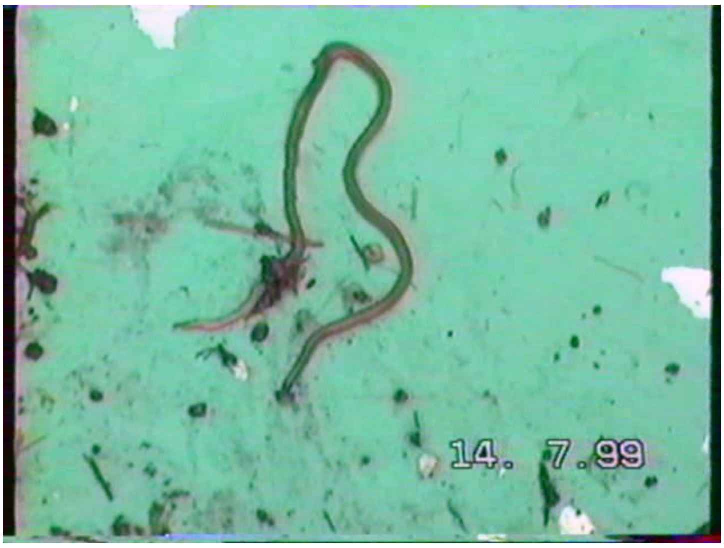

Bipalium kewense K34

#	Date	Locality	Department / State	Country - Continent	Origin of data
**K34**	14/09/2014	Urt	Pyrénées-Atlantiques	Met. France - Europe	Chanderot, Vincent

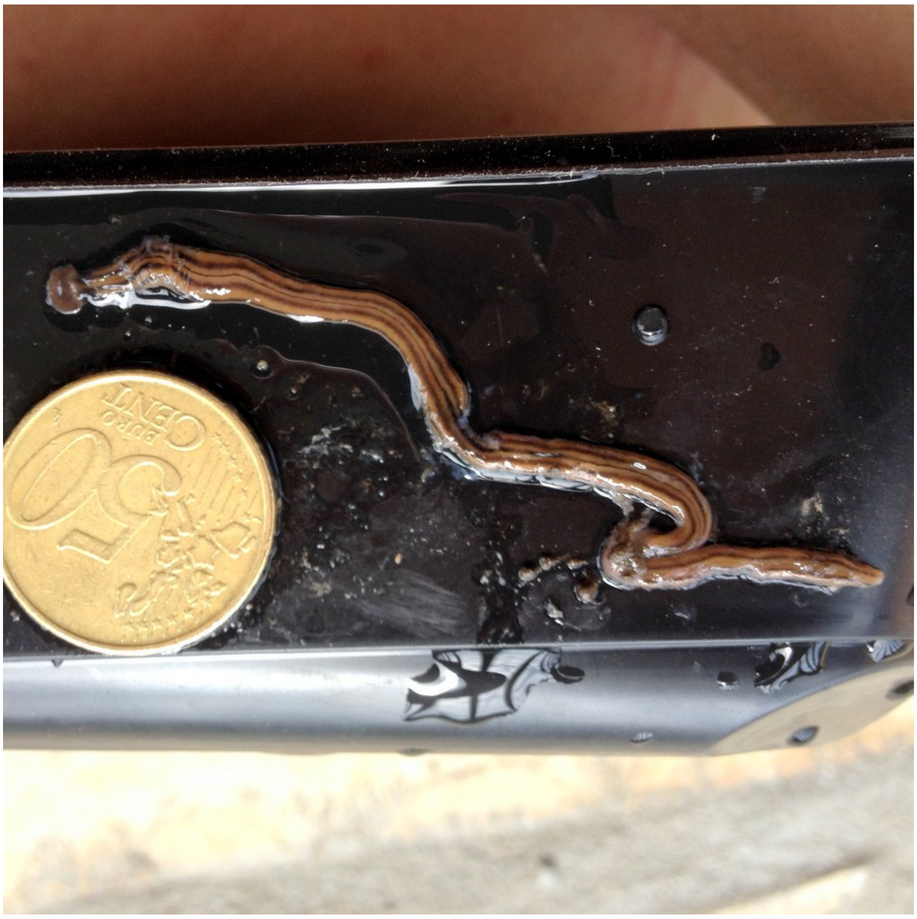

Bipalium kewense K35

#	Date	Locality	Department / State	Country - Continent	Origin of data
**K35**	12/08/2017	Ustaritz	Pyrénées-Atlantiques	Met. France - Europe	Lescourret, Monique & Bernard

Note: length of animal = 17 cm

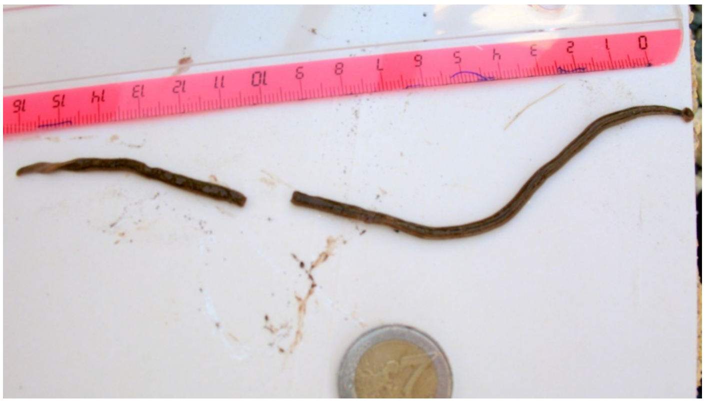

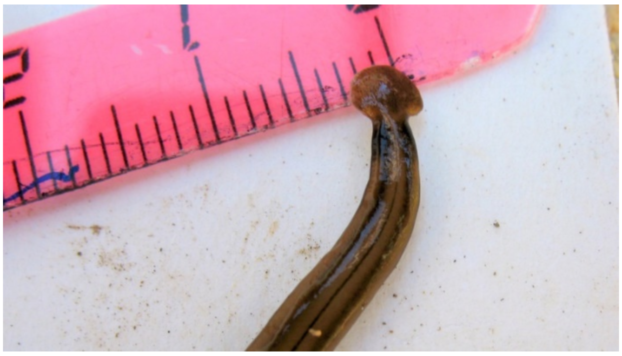

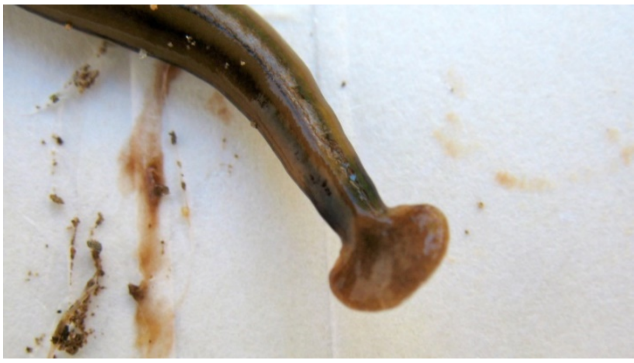

Bipalium kewense K36

#	Date	Locality	Department / State	Country - Continent	Origin of data
**K36**	14/09/2014	Villefranque	Pyrénées-Atlantiques	Met. France - Europe	Consent not obtained

Diversibipalium multilineatum: Records

Records of *Diversibipalium multilineatum* identified from photographs.

#	Date	Locality	Department / State	Country - Continent	Origin
**M01**	27/06/2010	Longages	Haute-Garonne	Met. France - Europe	Lombard, Yoann
**M02**	22/03/2011	Longages	Haute-Garonne	Met. France - Europe	Lombard, Yoann
**M03**	06/07/2016	Saint-Egrève	Isère	Met. France - Europe	Tuaillon, Jean-Louis
**M04**	17/05/2017	Saint-Egrève	Isère	Met. France - Europe	Tuaillon, Jean-Louis
**M05**	27/06/2016	Benquet	Landes	Met. France - Europe	Broustaut, François
**M06**	28/03/2014	Cahors (Hothouse)	Lot	Met. France - Europe	Consent not obtained
**M07**	04/07/2014	Andilly (Hothouse)	Val d'Oise	Met. France - Europe	Burel, Jonathan *
**M08**	27/04/2015	Magny-en-Vexin	Val d'Oise	Met. France - Europe	Mellac, Céline
**M09**	29/05/2016	Magny-en-Vexin	Val d'Oise	Met. France - Europe	Mellac, Céline
**M10**	19/04/2010	Sames	Pyrénées-Atlantiques	Met. France - Europe	Grenier-Falaise, Nadine
**M11**	07/04/2017	Billère	Pyrénées-Atlantiques	Met. France - Europe	Vincent, Jean-François

* FREDON.

Diversibipalium multilineatum M01-M02

Note: two observations, 2010 and 2011, same locality, same observer.

#	Date	Locality	Department / State	Country - Continent	Origin
**M01**	27/06/2010	Longages	Haute-Garonne	Met. France - Europe	Lombard, Yoann

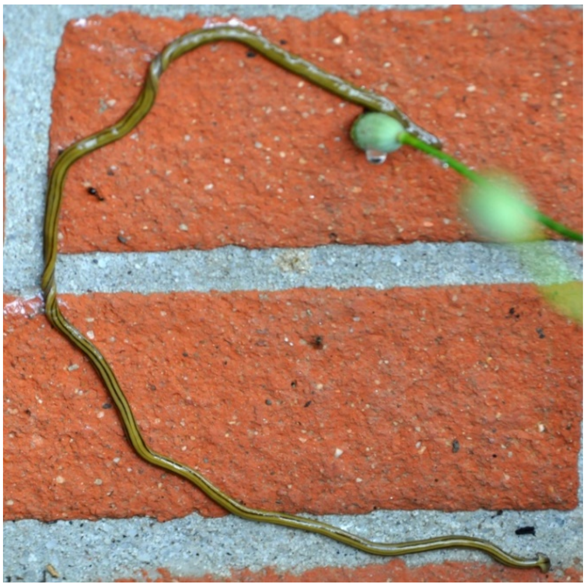

#	Date	Locality	Department / State	Country - Continent	Origin
**M02**	22/03/2011	Longages	Haute-Garonne	Met. France - Europe	Lombard, Yoann

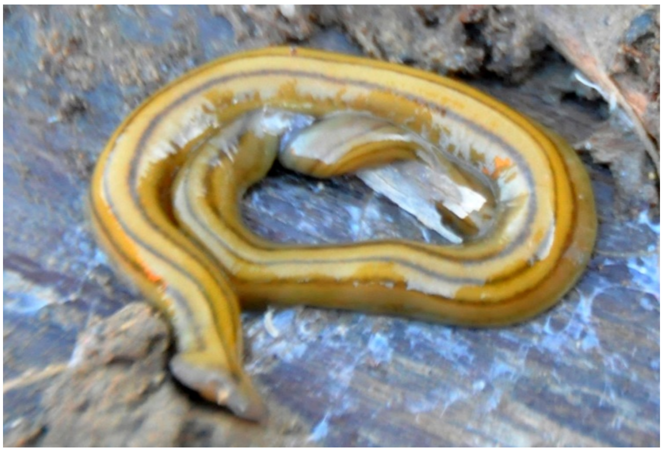

Diversibipalium multilineatum M03-M04

Two observations in the same garden, 2016 and 2017.

#	Date	Locality	Department / State	Country - Continent	Origin
**M03**	06/07/2016	Saint-Egrève	Isère	Met. France - Europe	Tuaillon, Jean-Louis

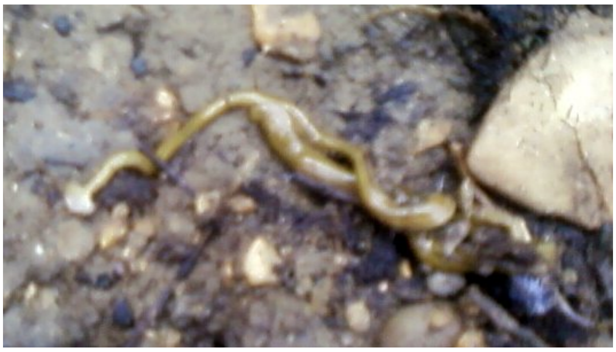

#	Date	Locality	Department / State	Country - Continent	Origin
**M04**	17/05/2017	Saint-Egrève	Isère	Met. France - Europe	Tuaillon, Jean-Louis

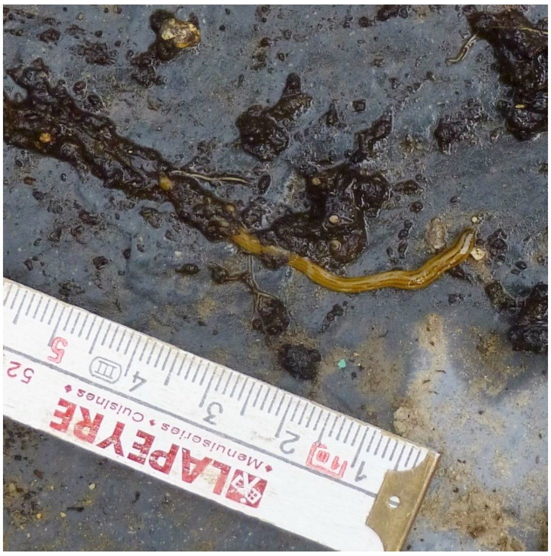

Diversibipalium multilineatum M05

#	Date	Locality	Department / State	Country - Continent	Origin
**M05**	27/06/2016	Benquet	Landes	Met. France - Europe	Broustaut, François

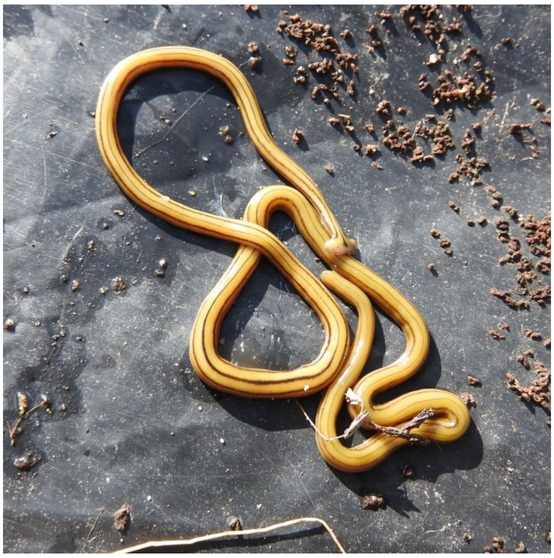
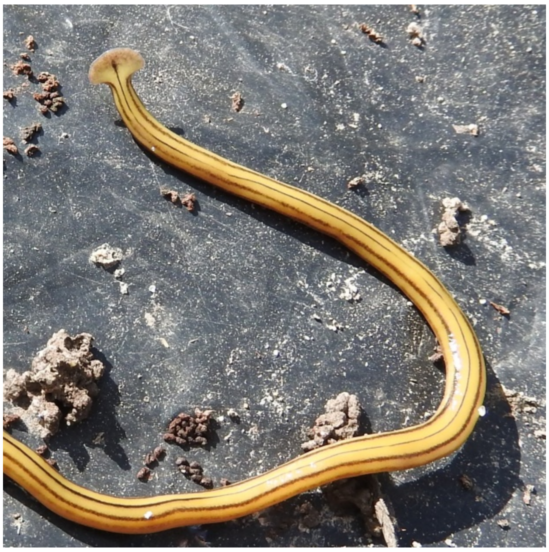

Diversibipalium multilineatum M06

#	Date	Locality	Department / State	Country - Continent	Origin
**M06**	28/03/2014	Cahors (Hothouse)	Lot	Met. France - Europe	Consent not obtained

Diversibipalium multilineatum M07

#	Date	Locality	Department / State	Country - Continent	Origin
**M07**	04/07/2014	Andilly (Hothouse)	Val d'Oise	Met. France - Europe	Burel, Jonathan *

Note: with *Obama nungara*

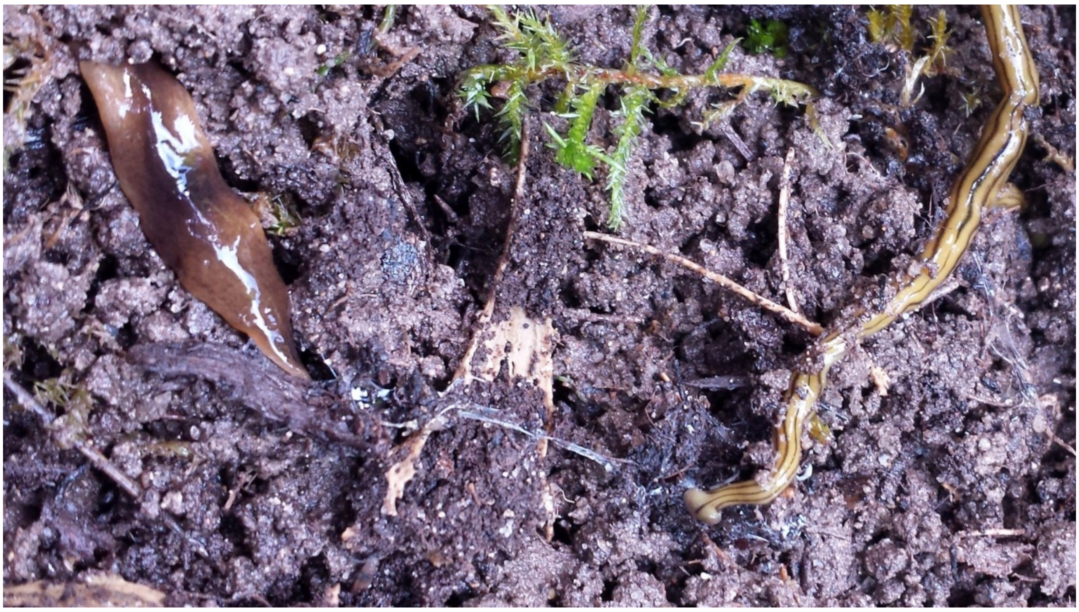

Diversibipalium multilineatum M08-M09

Note: two observations, same garden, 2015 and 2016.

#	Date	Locality	Department / State	Country - Continent	Origin
**M08**	27/04/2015	Magny-en-Vexin	Val d'Oise	Met. France - Europe	Mellac, Céline

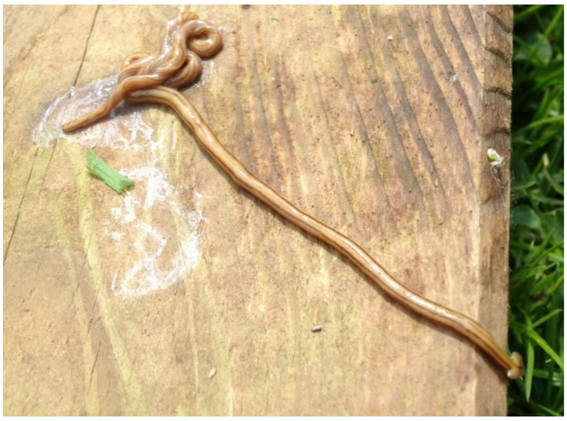

#	Date	Locality	Department / State	Country - Continent	Origin
**M09**	29/05/2016	Magny-en-Vexin	Val d'Oise	Met. France - Europe	Mellac, Céline

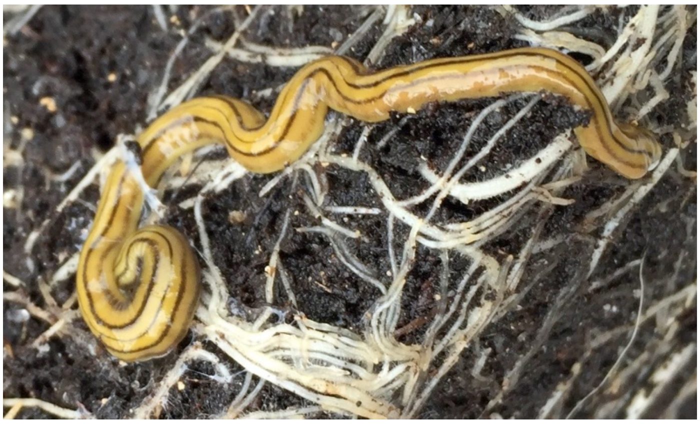

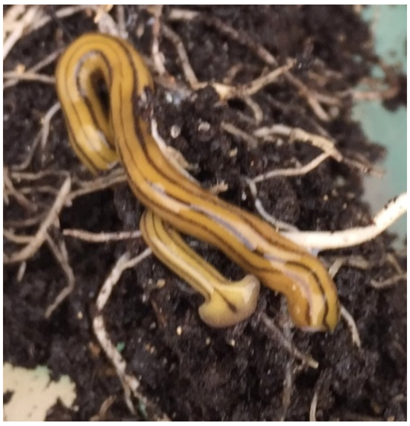

Diversibipalium multilineatum M10

#	Date	Locality	Department / State	Country - Continent	Origin
**M10**	19/04/2010	Sames	Pyrénées-Atlantiques	Met. France - Europe	Grenier-Falaise, Nadine

Note: early record, 2010

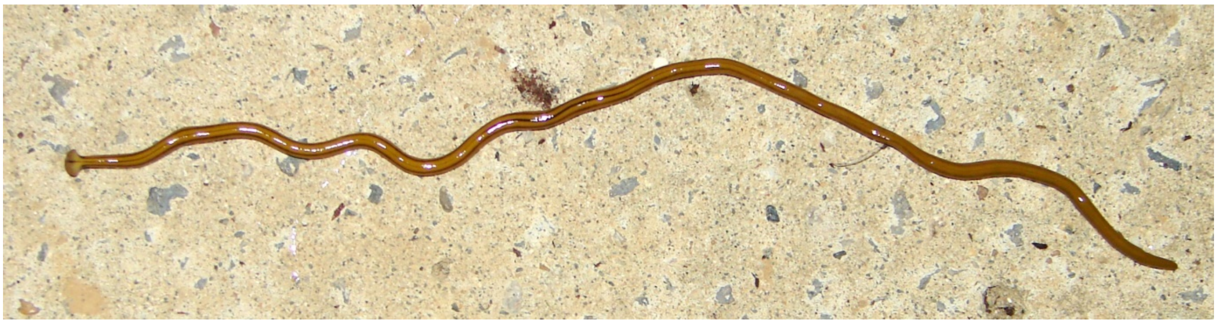

Diversibipalium multilineatum M11

#	Date	Locality	Department / State	Country - Continent	Origin
**M11**	07/04/2017	Billère	Pyrénées-Atlantiques	Met. France - Europe	Vincent, Jean-François

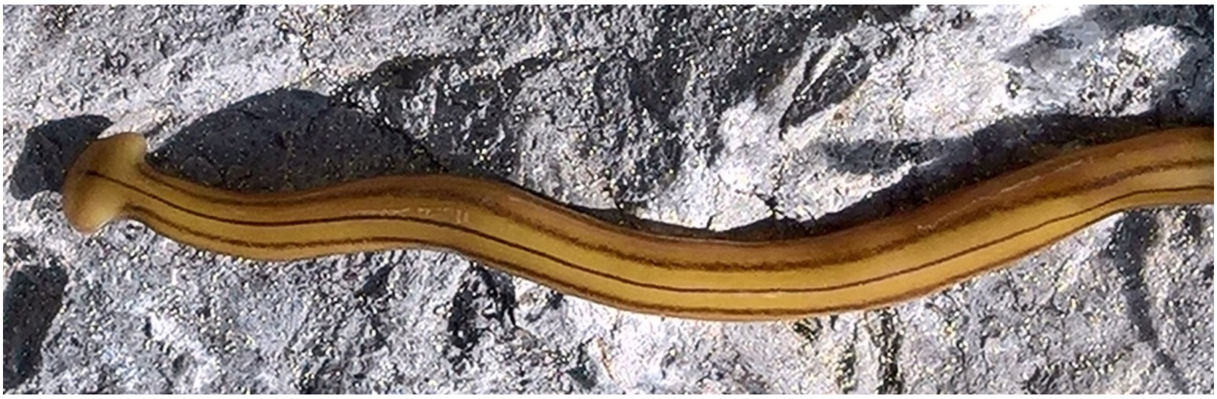

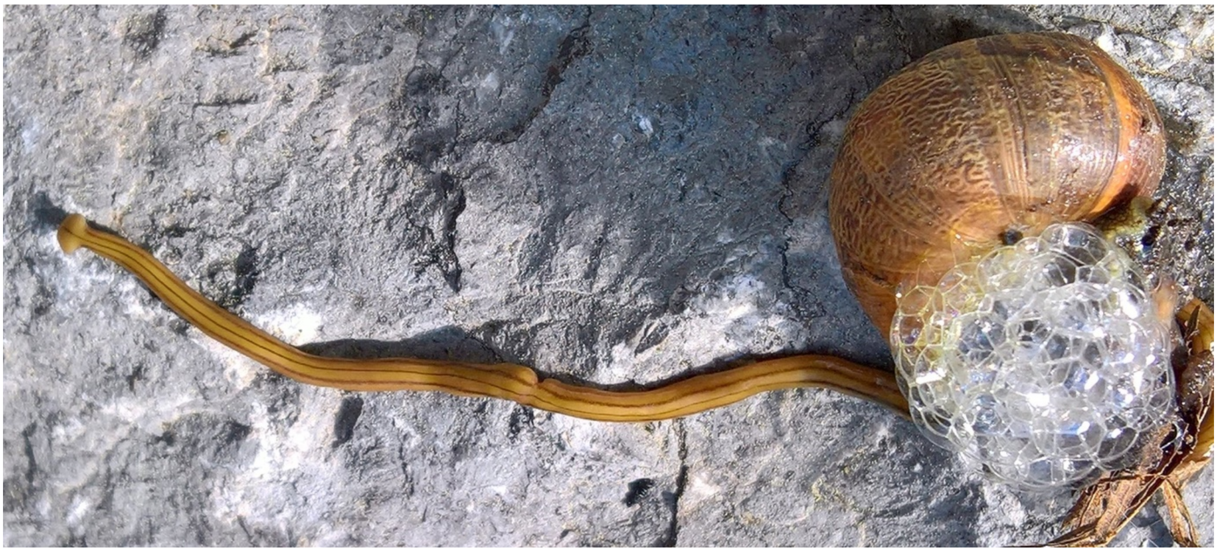

Bipalium vagum: Records

Records of *Bipalium vagum* identified from photographs

#	Date	Locality	Department / State	Country - Continent	Origin
**V01**	21/06/2005	Cayenne	French Guiana	French Guiana – S. America	Girault, Rémi
**V02**	15/05/2017	Macouria	French Guiana	French Guiana – S. America	Boutin, Élodie
**V03**	12/05/2017	Saint-Laurent-du-Maroni	French Guiana	French Guiana – S. America	Muraine, François Xavier
**V04**	26/07/2017	Saül	French Guiana	French Guiana – S. America	Sant, Sébastien
**V05**	21/08/2017	Petit-Bourg	Guadeloupe	French Guiana – S. America	De Tienda, Marine
**V06**	24/11/2013	Gosier	Guadeloupe	Guadeloupe - C. America	Consent not obtained
**V07**	30/10/2016	Gosier	Guadeloupe	Guadeloupe - C. America	Brisson, Bernard
**V08**	22/11/2013	Petit Bourg	Guadeloupe	Guadeloupe - C. America	Oettly, Olivier
**V09**	22/11/2014	Petit Bourg	Guadeloupe	Guadeloupe - C. America	Marques, Maryvonne
**V10**	29/04/2011	Petit-Bourg	Guadeloupe	Guadeloupe - C. America	Guezennec, Pierre et Claudine
**V11**	21/10/2017	Petit-Canal	Guadeloupe	Guadeloupe - C. America	Charles, Laurent
**V12**	29/11/2016	Le Moule	Guadeloupe	Guadeloupe - C. America	Consent non obtained
**V13**	25/07/2010	La Trinité	Martinique	Martinique - C. America	Delannoye, Régis
**V14**	18/11/2015	Morne Vert	Martinique	Martinique - C. America	Coulis, Mathieu
**V15**	05/01/2018	Trois Ilets	Martinique	Martinique - C. America	Consent non obtained
**V16**	01/04/2014	Saint Barthélemy	Saint Barthélemy	Saint Barthélemy - C. America	Moulard, Grégory
**V17**	01/05/2014	Saint Barthélemy	Saint Barthélemy	Saint Barthélemy - C. America	Consent not obtained
**V18**	11/05/2014	Saint Martin	Saint Martin	Saint Martin – C. America	Yokoyama, Mark
**V19**	21/11/2015	Avirons	La Réunion	La Réunion - Africa	Consent not obtained
**V20**	23/03/2017	Bras Panon	La Réunion	La Réunion - Africa	Saman-Latchimy, Teddy
**V21**	29/03/2017	Le Tampon	La Réunion	La Réunion - Africa	Consent not obtained
**V22**	26/10/2014	Petite Ile	La Réunion	La Réunion - Africa	Abonnenc, José
**V23**	12/03/2016	Petite Ile	La Réunion	La Réunion - Africa	Le Gars, René
**V24**	16/05/2014	Saint Louis	La Réunion	La Réunion - Africa	Faujour, Anne
**V25**	08/04/2014	Saint Paul	La Réunion	La Réunion - Africa	Consent not obtained
**V26**	16/03/2016	Saint Pierre	La Réunion	La Réunion - Africa	Collet, Jean
**V27**	10/03/2013	Sainte Marie	La Réunion	La Réunion - Africa	Fontaine, Romuald
**V28**	06/03/2016	Sainte Marie	La Réunion	La Réunion - Africa	Fontaine, Romuald
**V29**	12/02/2009	unknown	La Réunion	La Réunion - Africa	Gilson, Michel
**V30**	03/03/2010	unknown	La Réunion	La Réunion - Africa	Gilson, Michel
**V31**	01/05/2011	unknown	La Réunion	La Réunion - Africa	Martiré, Dominique
**V32**	28/10/2013	unknown	La Réunion	La Réunion - Africa	Martiré, Dominique
**V33**	17/08/2015	unknown	La Réunion	La Réunion - Africa	Lacoste, Marie

Bipalium vagum V01

#	Date	Locality	Department / State	Country - Continent	Origin
**V01**	21/06/2005	Cayenne	French Guiana	French Guiana – S. America	Girault, Rémi

Note: early record (2005).

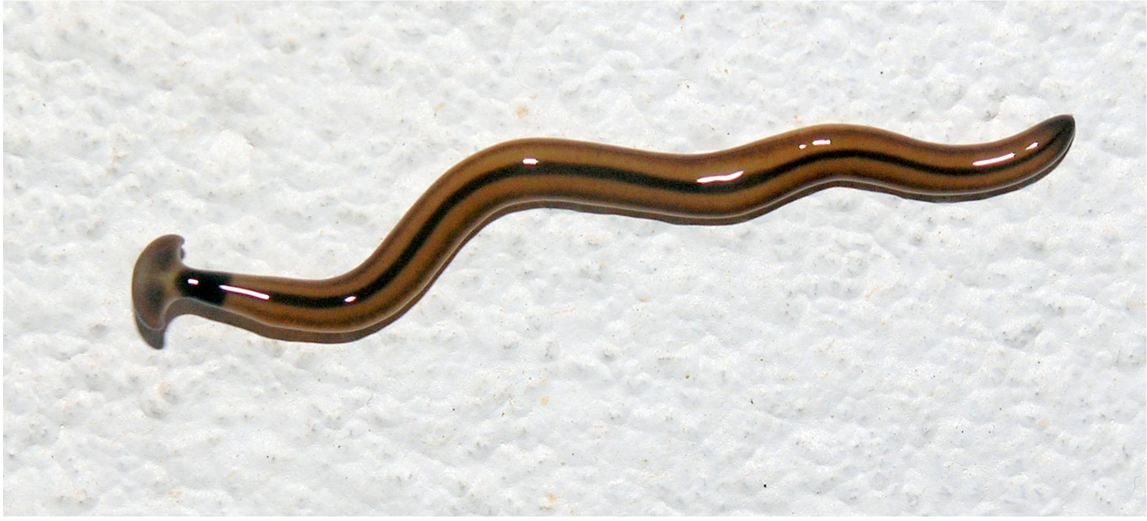

Bipalium vagum V02

#	Date	Locality	Department / State	Country - Continent	Origin
**V02**	15/05/2017	Macouria	French Guiana	French Guiana – S. America	Boutin, Élodie

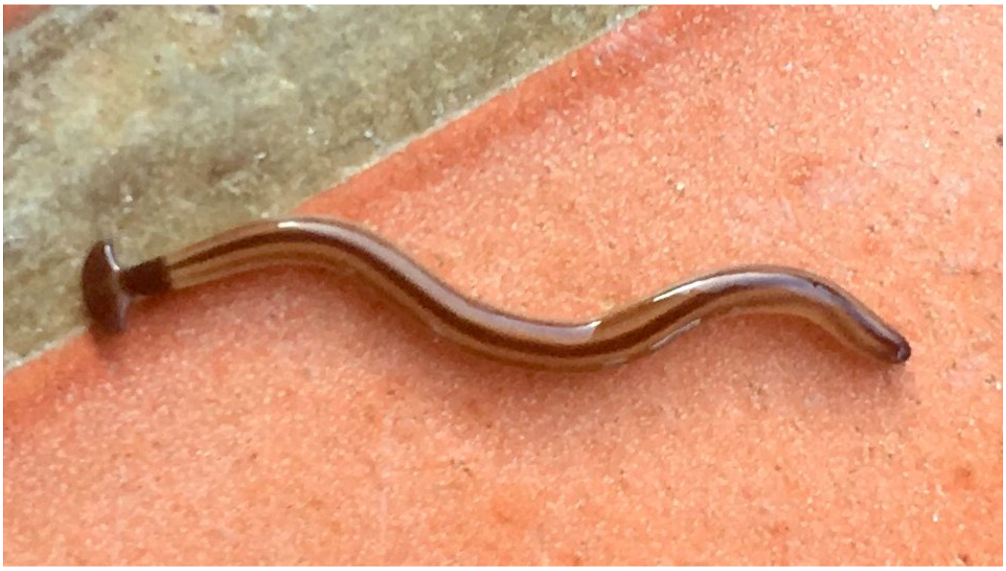

Bipalium vagum V03

#	Date	Locality	Department / State	Country - Continent	Origin
**V03**	12/05/2017	Saint-Laurent-du-Maroni	French Guiana	French Guiana – S. America	Muraine, François Xavier

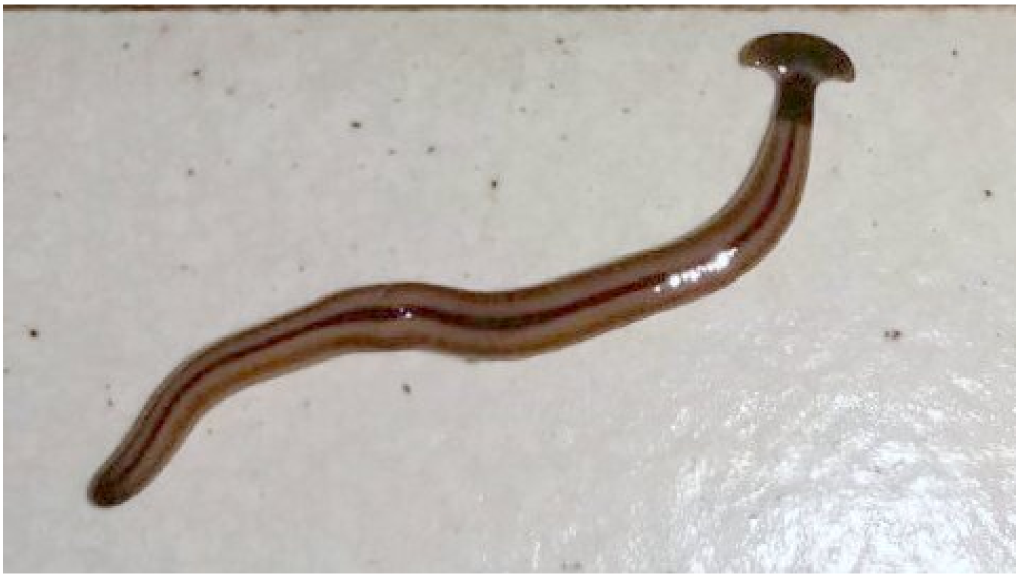

Bipalium vagum V04

#	Date	Locality	Department / State	Country - Continent	Origin
**V04**	26/07/2017	Saül	French Guiana	French Guiana – S. America	Sant, Sébastien

Note: the diameter of the 10 euro cent coin is 19.75 mm; the length of the worm is 36 mm at least.

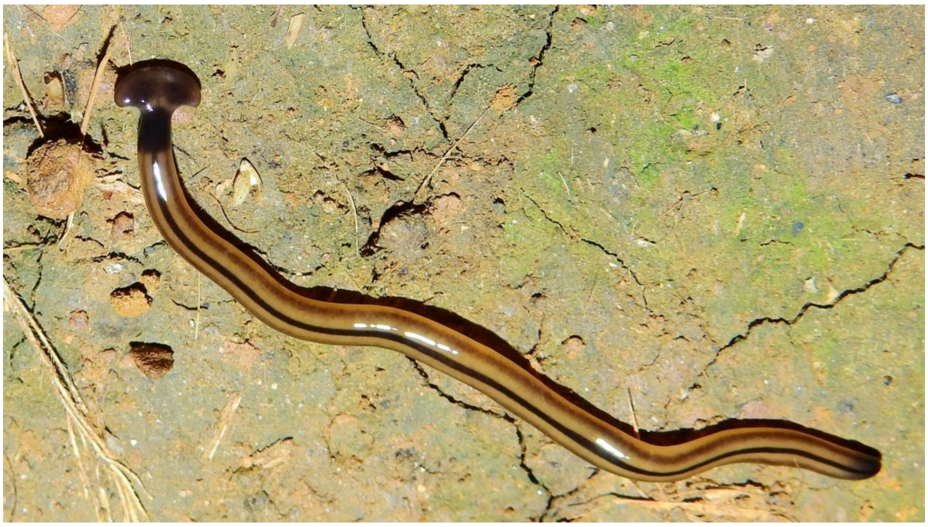

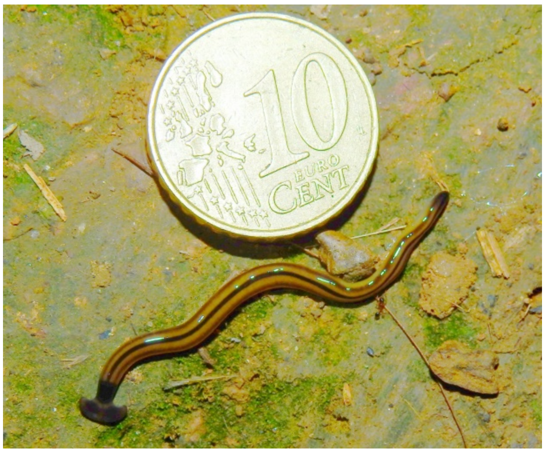

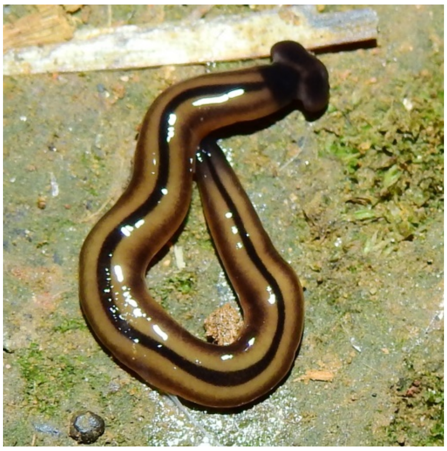

Bipalium vagum V05

#	Date	Locality	Department / State	Country - Continent	Origin
**V05**	21/08/2017	Petit-Bourg	Guadeloupe	French Guiana – S. America	De Tienda, Marine

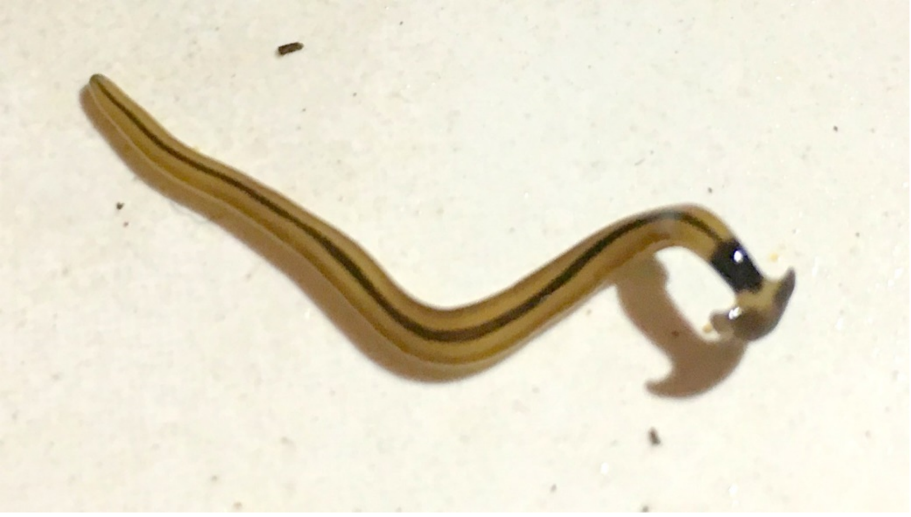

Bipalium vagum V06

#	Date	Locality	Department / State	Country - Continent	Origin
**V06**	24/11/2013	Gosier	Guadeloupe	Guadeloupe - C. America	Consent not obtained

Bipalium vagum V07

#	Date	Locality	Department / State	Country - Continent	Origin
**V07**	30/10/2016	Gosier	Guadeloupe	Guadeloupe - C. America	Brisson, Bernard

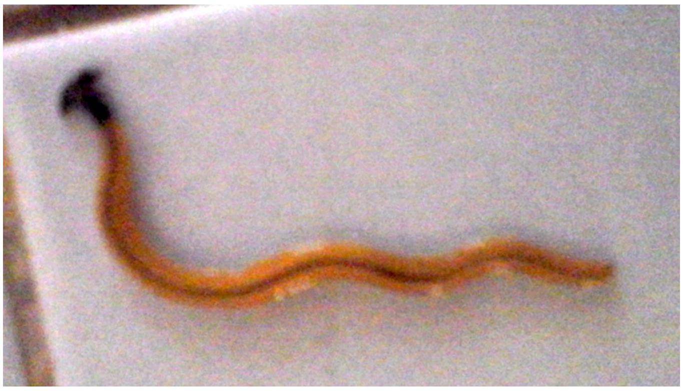

Bipalium vagum V08

#	Date	Locality	Department / State	Country - Continent	Origin
**V08**	22/11/2013	Petit Bourg	Guadeloupe	Guadeloupe - C. America	Oettly, Olivier

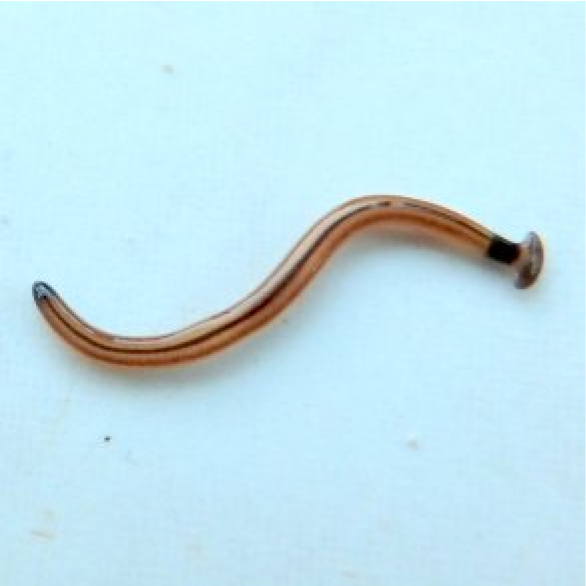

Bipalium vagum V09

#	Date	Locality	Department / State	Country - Continent	Origin
**V09**	22/11/2014	Petit Bourg	Guadeloupe	Guadeloupe - C. America	Marques, Maryvonne

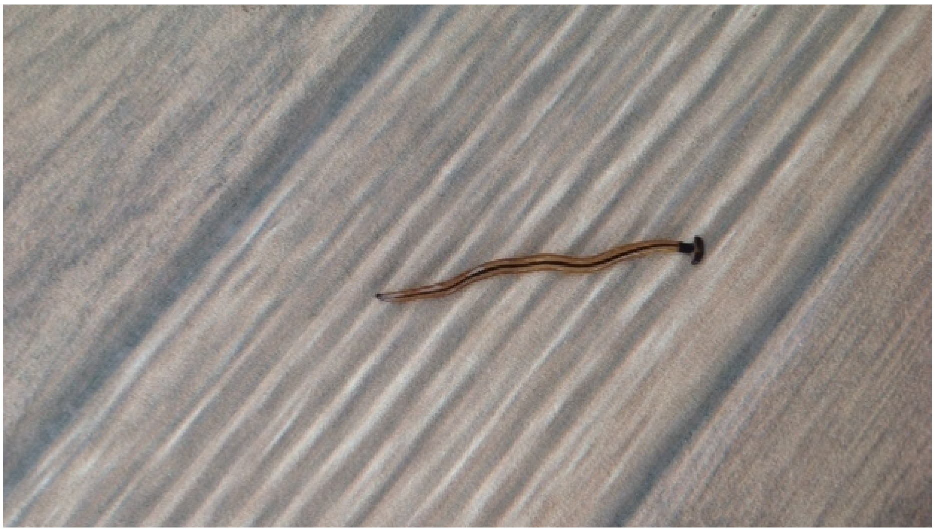

Bipalium vagum V10

#	Date	Locality	Department / State	Country - Continent	Origin
**V10**	29/04/2011	Petit-Bourg	Guadeloupe	Guadeloupe - C. America	Guezennec, Pierre et Claudine

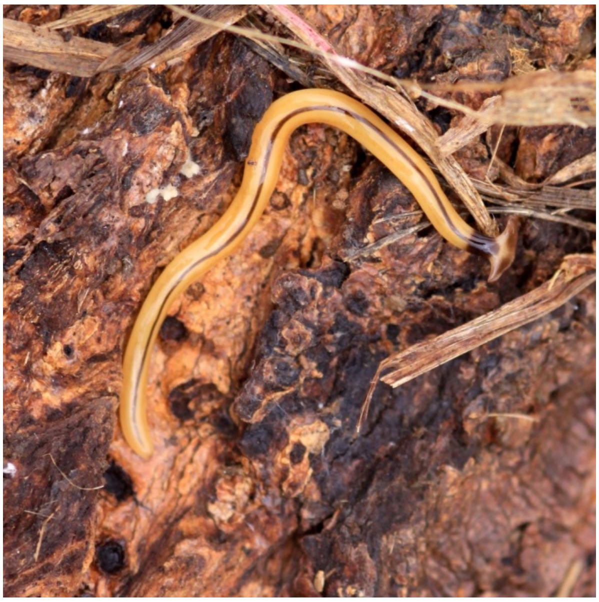

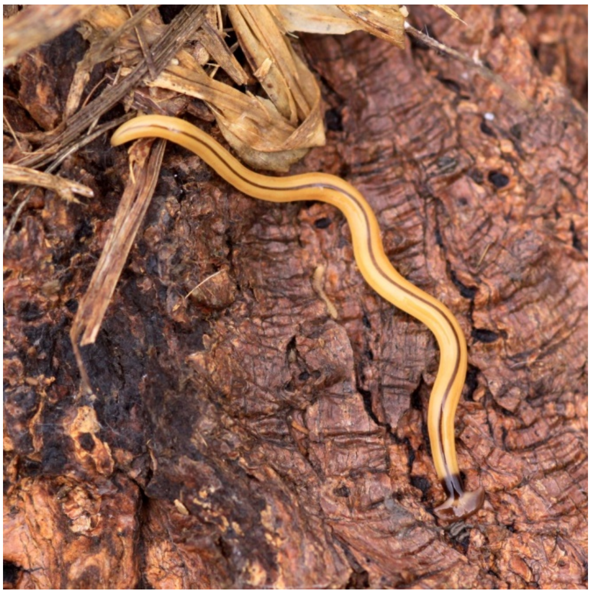

Bipalium vagum V11

#	Date	Locality	Department / State	Country - Continent	Origin
**V11**	21/10/2017	Petit-Canal	Guadeloupe	Guadeloupe - C. America	Charles, Laurent

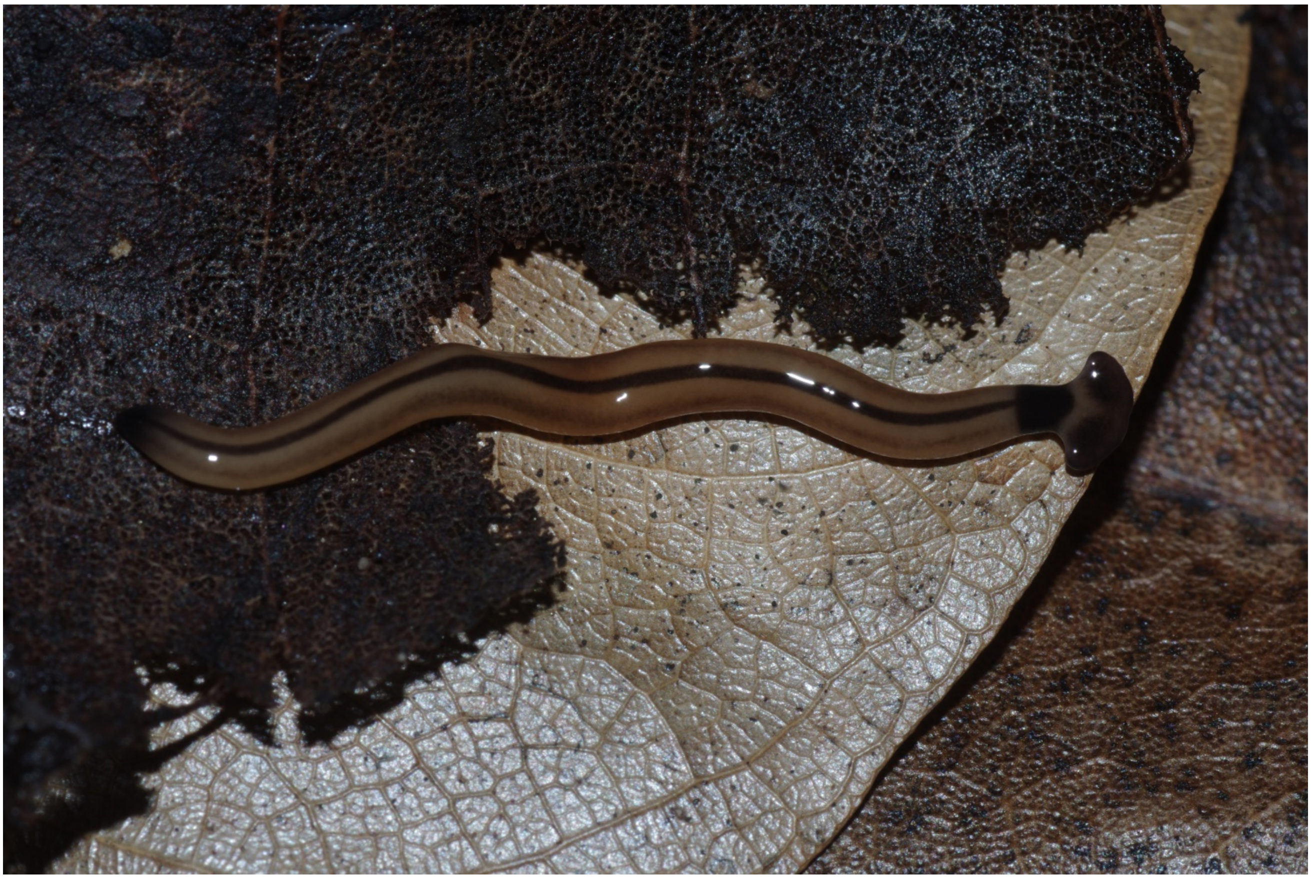

Bipalium vagum V12

#	Date	Locality	Department / State	Country - Continent	Origin
**V12**	29/11/2016	Le Moule	Guadeloupe	Guadeloupe - C. America	Consent not obtained

Bipalium vagum V13

#	Date	Locality	Department / State	Country - Continent	Origin
**V13**	25/07/2010	La Trinité	Martinique	Martinique - C. America	Delannoye, Régis

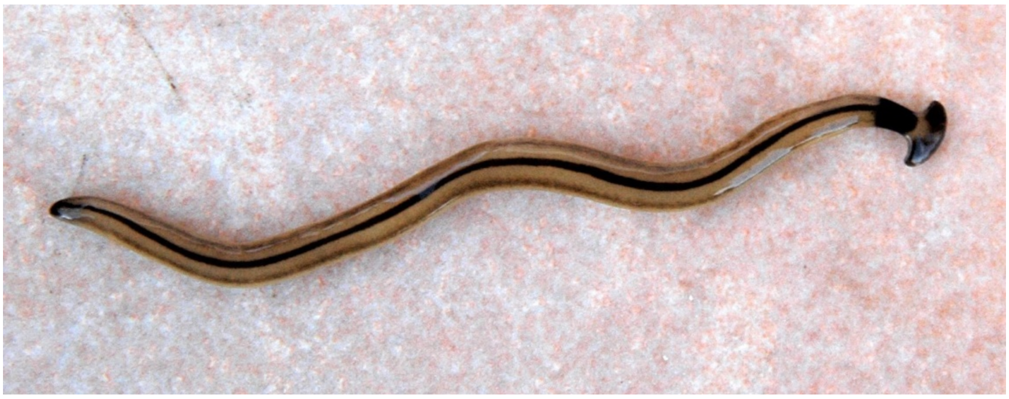

Bipalium vagum V14

#	Date	Locality	Department / State	Country - Continent	Origin
V14	18/11/2015	Morne Vert	Martinique	Martinique - C. America	Coulis, Mathieu

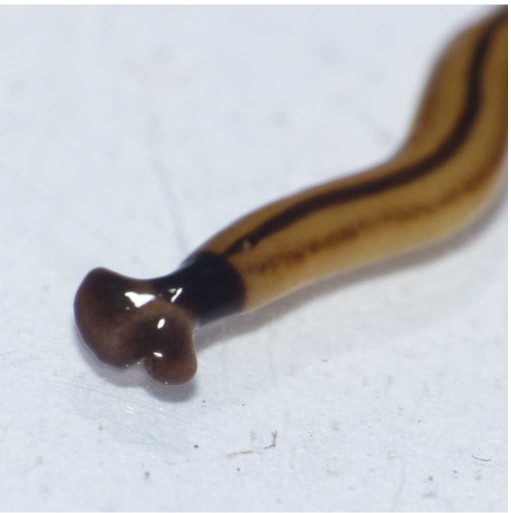

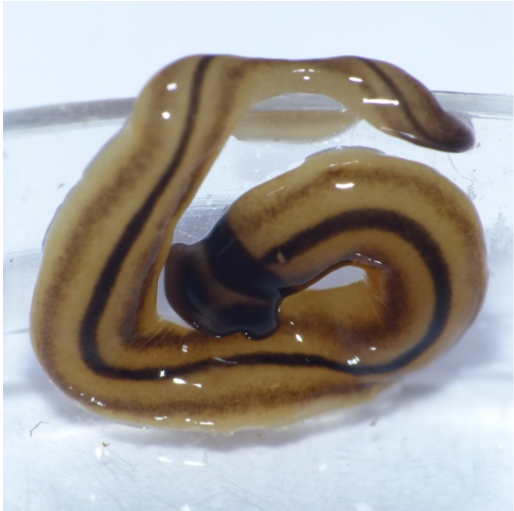

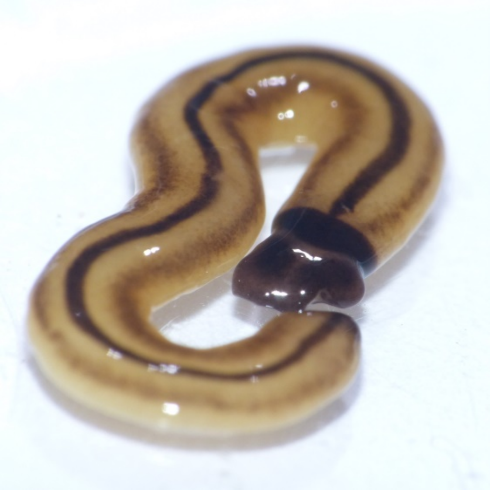

Bipalium vagum V15

#	Date	Locality	Department / State	Country - Continent	Origin
V15	05/01/2018	Trois Ilets	Martinique	Martinique - C. America	Consent not obtained

Bipalium vagum V16

#	Date	Locality	Department / State	Country - Continent	Origin
**V16**	01/04/2014	Saint Barthélemy	Saint Barthélemy	Saint Barthélemy - C. America	Moulard, Grégory

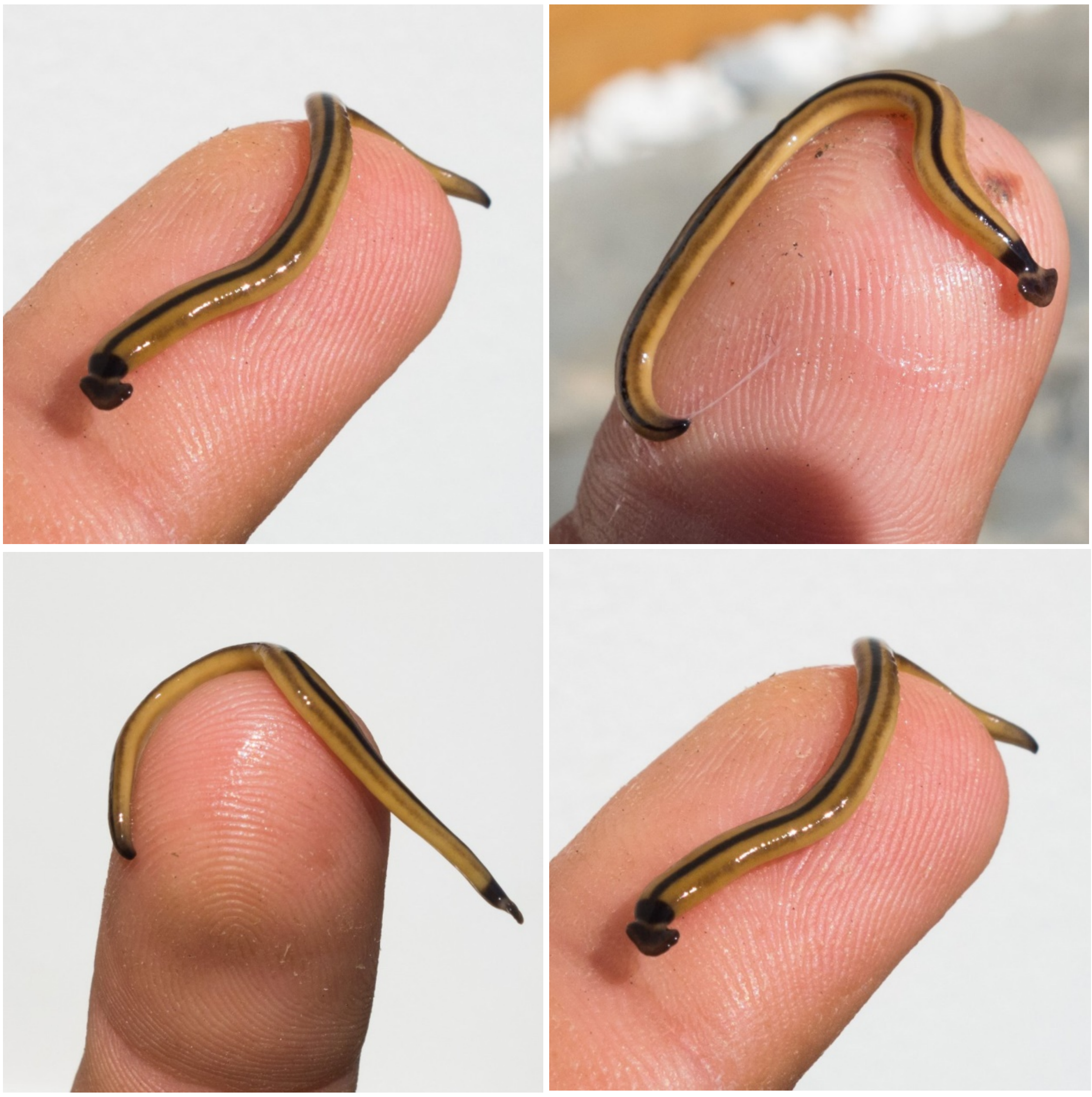

Bipalium vagum V17

#	Date	Locality	Department / State	Country - Continent	Origin
**V17**	01/05/2014	Saint Barthélemy	Saint Barthélemy	Saint Barthélemy - C. America	Consent not obtained

Bipalium vagum V18

#	Date	Locality	Department / State	Country - Continent	Origin
**V18**	11/05/2014	Saint Martin	Saint Martin	Saint Martin – C. America	Yokoyama, Mark

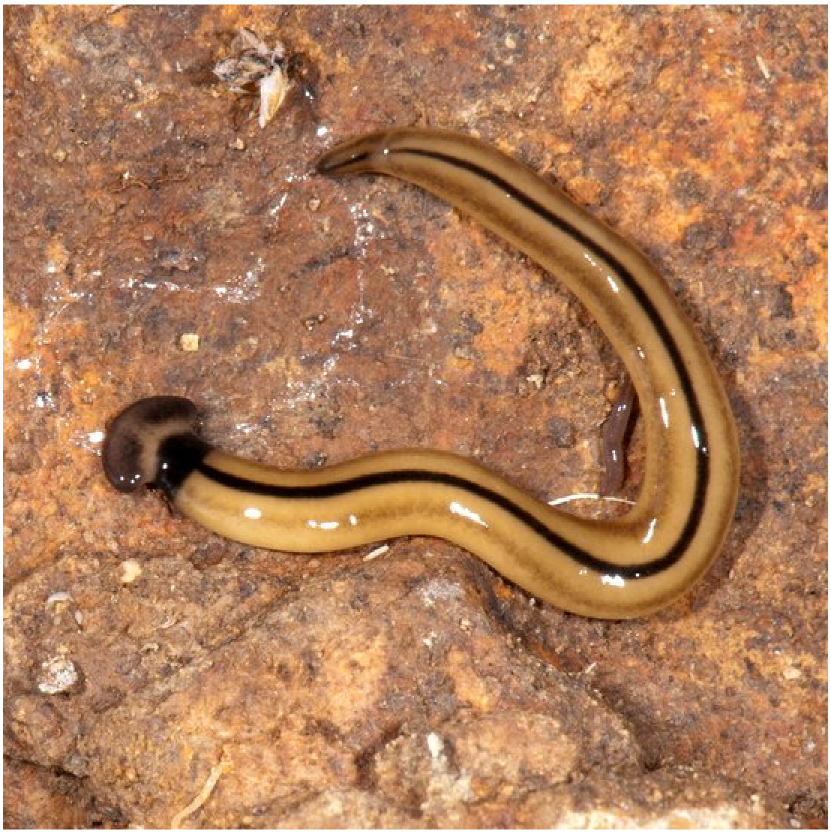

Bipalium vagum V19

#	Date	Locality	Department / State	Country - Continent	Origin
**V19**	21/11/2015	Avirons	La Réunion	La Réunion - Africa	Consent not obtained

Bipalium vagum V20

#	Date	Locality	Department / State	Country - Continent	Origin
**V20**	23/03/2017	Bras Panon	La Réunion	La Réunion - Africa	Saman-Latchimy, Teddy

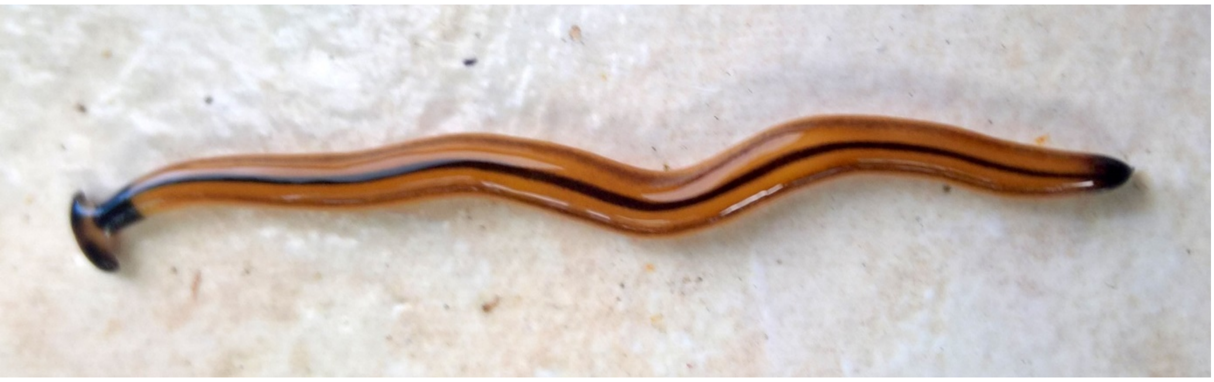

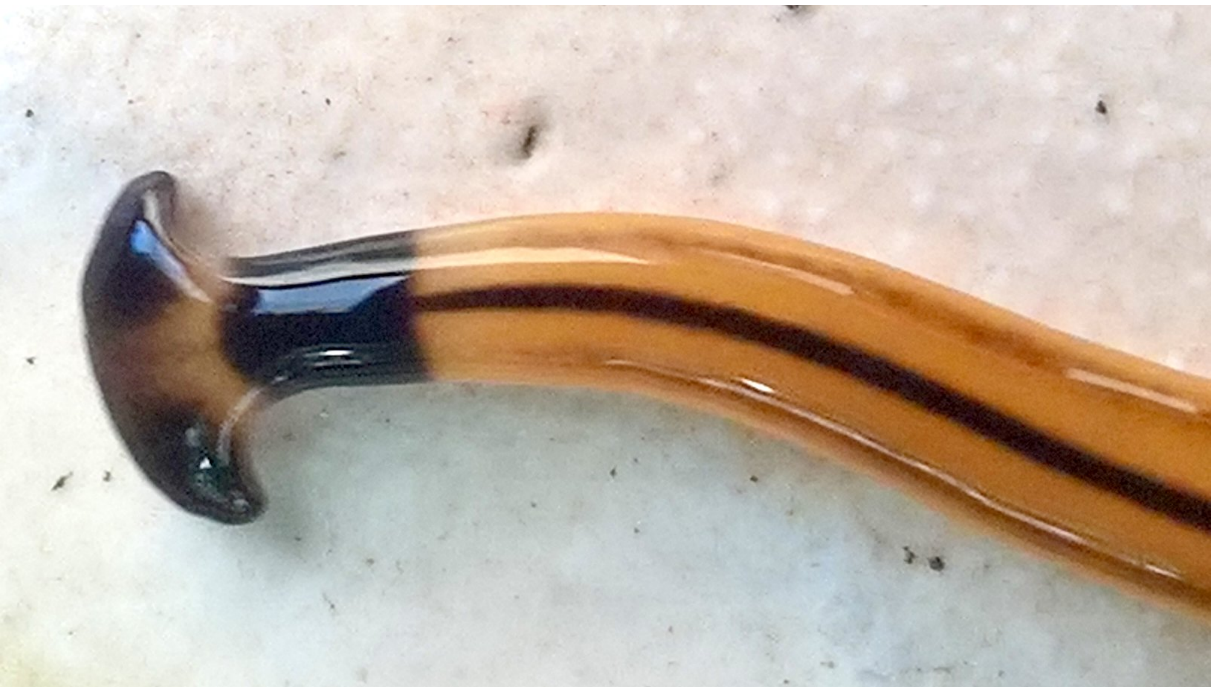

Bipalium vagum V21

#	Date	Locality	Department / State	Country - Continent	Origin
**V21**	29/03/2017	Le Tampon	La Réunion	La Réunion - Africa	Consent not obtained

Bipalium vagum V22

#	Date	Locality	Department / State	Country - Continent	Origin
**V22**	26/10/2014	Petite Ile	La Réunion	La Réunion - Africa	Abonnenc, José

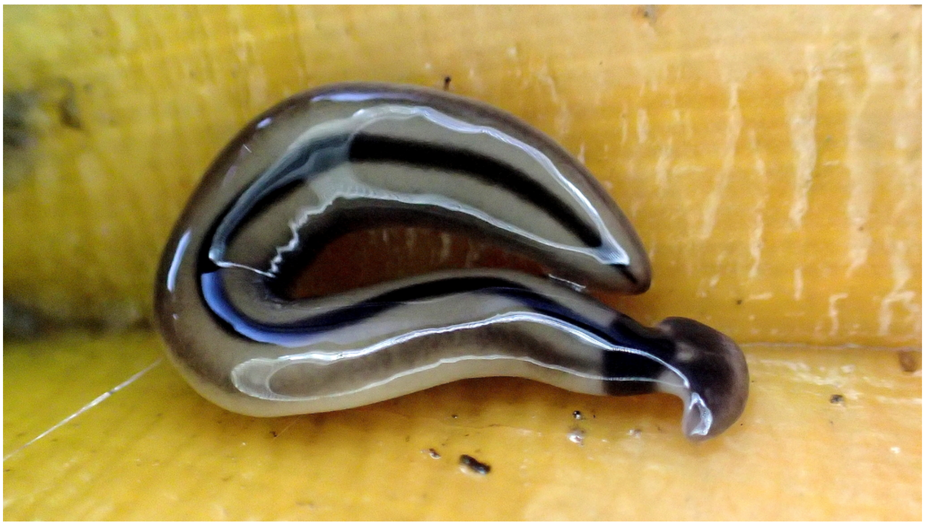

Bipalium vagum V23

#	Date	Locality	Department / State	Country - Continent	Origin
**V23**	12/03/2016	Petite Ile	La Réunion	La Réunion - Africa	Le Gars, René

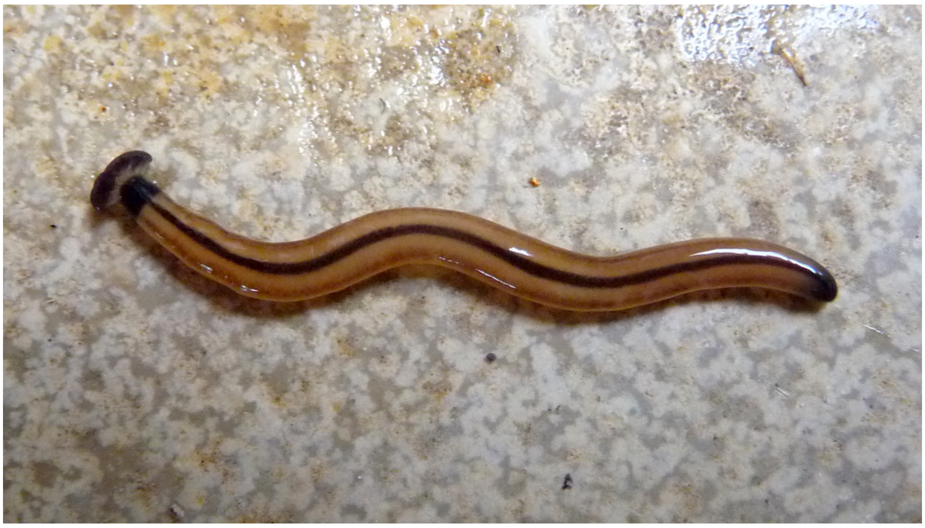

Bipalium vagum V24

#	Date	Locality	Department / State	Country - Continent	Origin
**V24**	16/05/2014	Saint Louis	La Réunion	La Réunion - Africa	Faujour, Anne

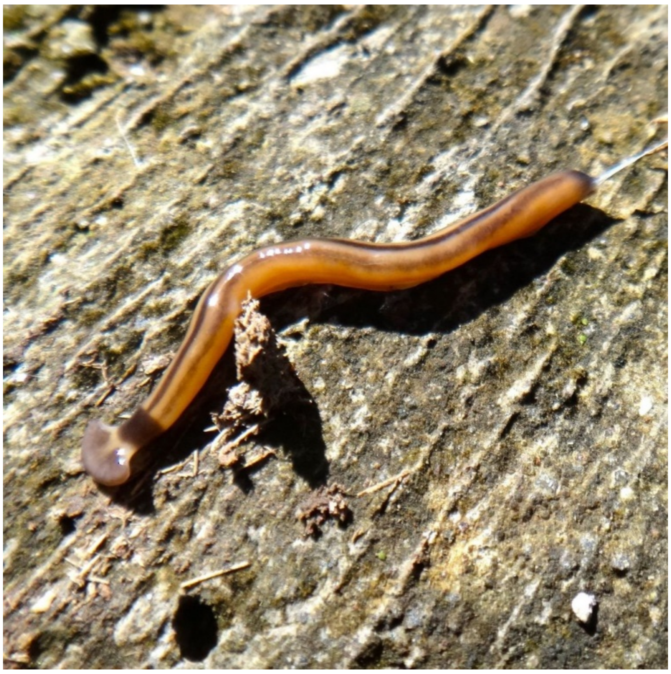

Bipalium vagum V25

#	Date	Locality	Department / State	Country - Continent	Origin
**V25**	08/04/2014	Saint Paul	La Réunion	La Réunion - Africa	Consent not obtained

Bipalium vagum V26

#	Date	Locality	Department / State	Country - Continent	Origin
**V26**	16/03/2016	Saint Pierre	La Réunion	La Réunion - Africa	Collet, Jean

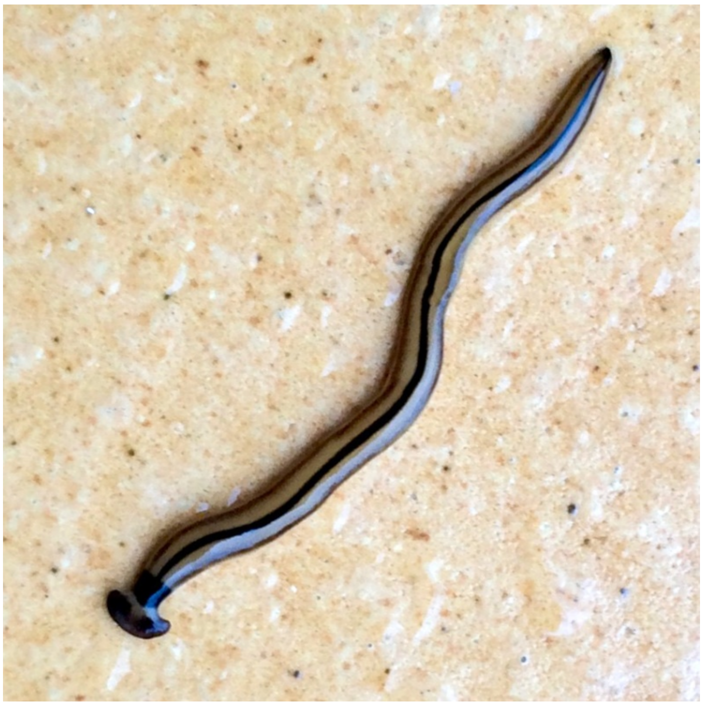

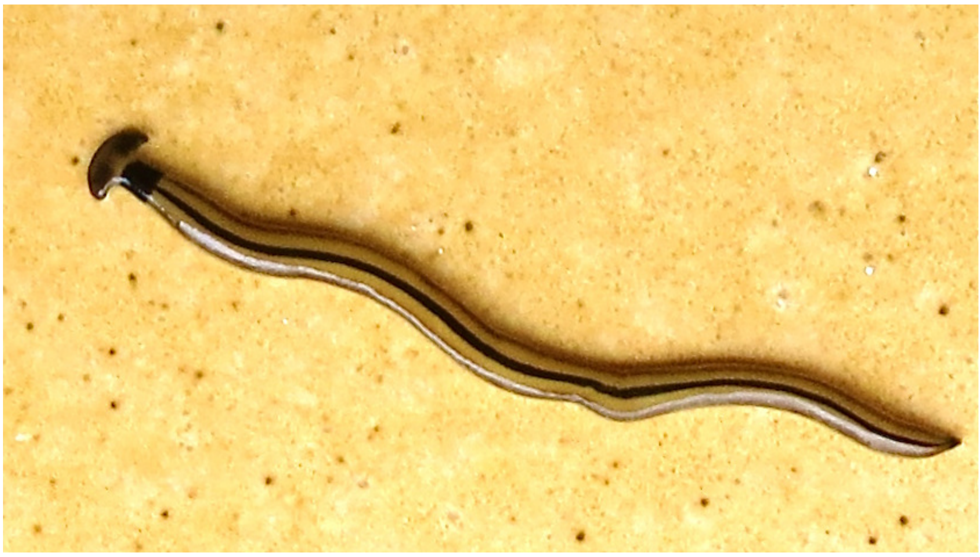

Bipalium vagum V27-V28

Note: two observations, 2013 and 2016

#	Date	Locality	Department / State	Country - Continent	Origin
**V27**	10/03/2013	Sainte Marie	La Réunion	La Réunion - Africa	Fontaine, Romuald

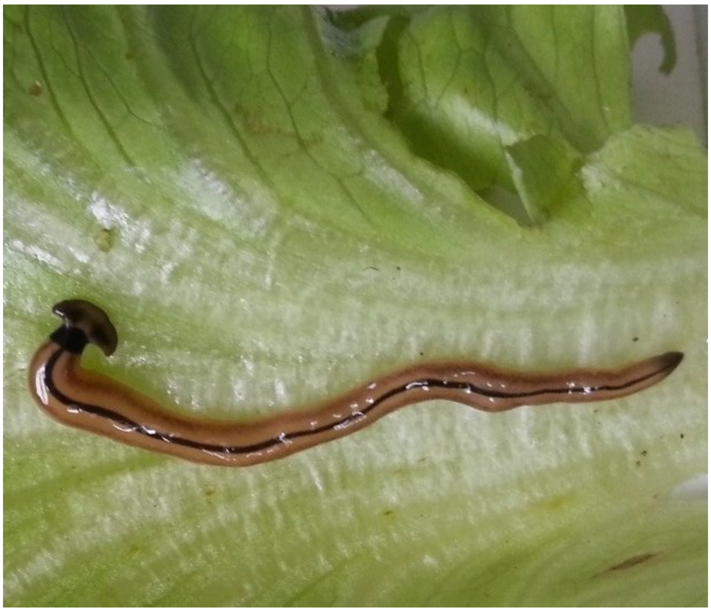

#	Date	Locality	Department / State	Country - Continent	Origin
**V28**	06/03/2016	Sainte Marie	La Réunion	La Réunion - Africa	Fontaine, Romuald

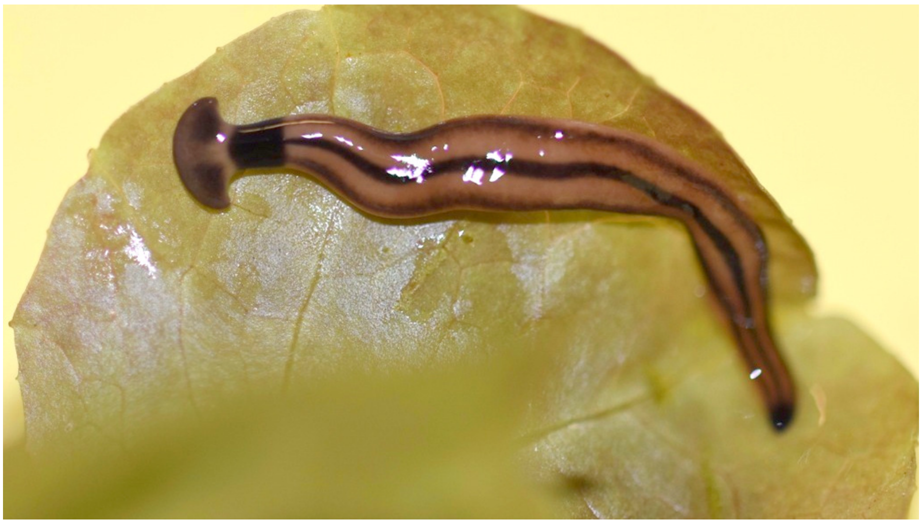

Bipalium vagum V29

#	Date	Locality	Department / State	Country - Continent	Origin
**V29**	12/02/2009	unknown	La Réunion	La Réunion - Africa	Gilson, Michel

Note: early record (2009).

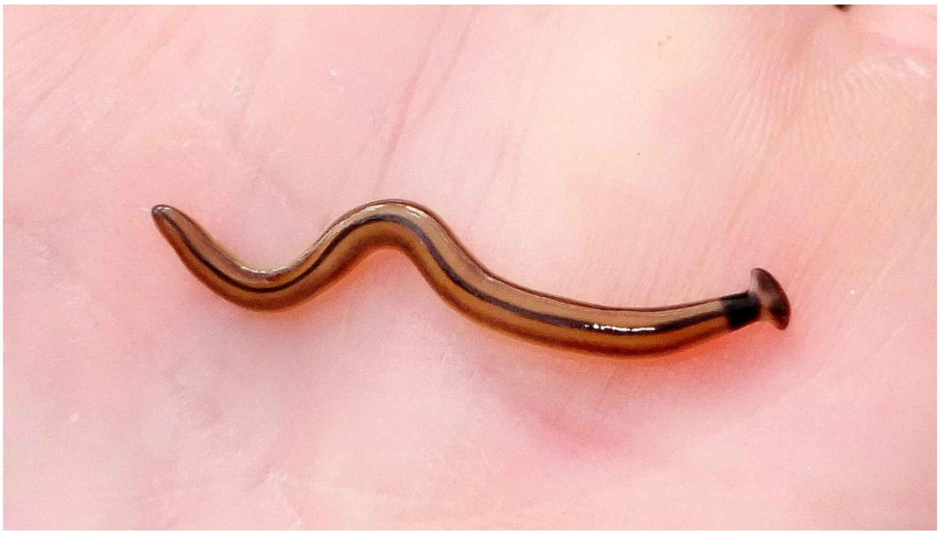

Bipalium vagum V30

#	Date	Locality	Department / State	Country - Continent	Origin
**V30**	03/03/2010	unknown	La Réunion	La Réunion - Africa	Gilson, Michel

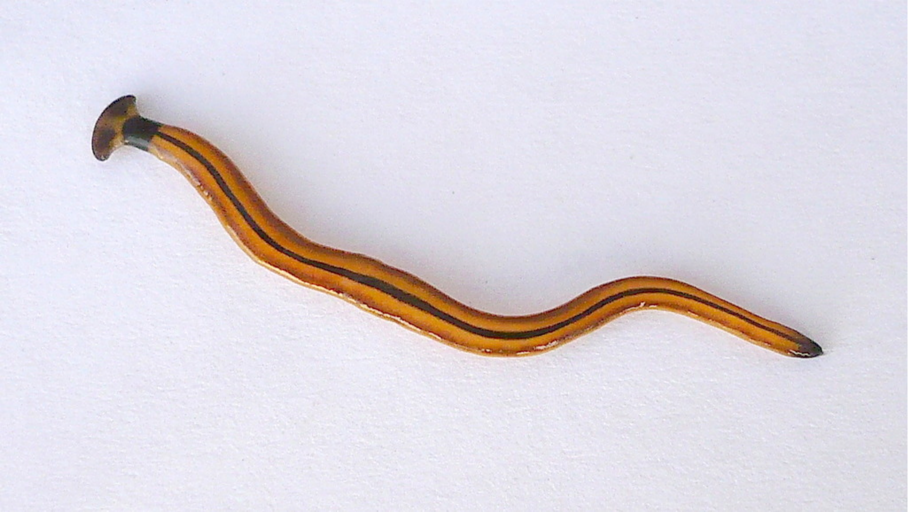

Bipalium vagum V31

#	Date	Locality	Department / State	Country - Continent	Origin
**V31**	01/05/2011	unknown	La Réunion	La Réunion - Africa	Martiré, Dominique

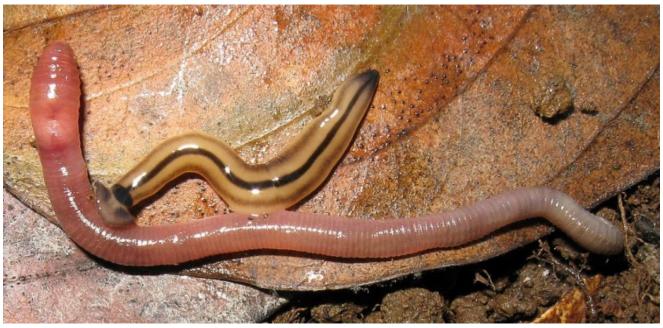

Bipalium vagum V32

#	Date	Locality	Department / State	Country - Continent	Origin
**V32**	28/10/2013	unknown	La Réunion	La Réunion - Africa	Martiré, Dominique

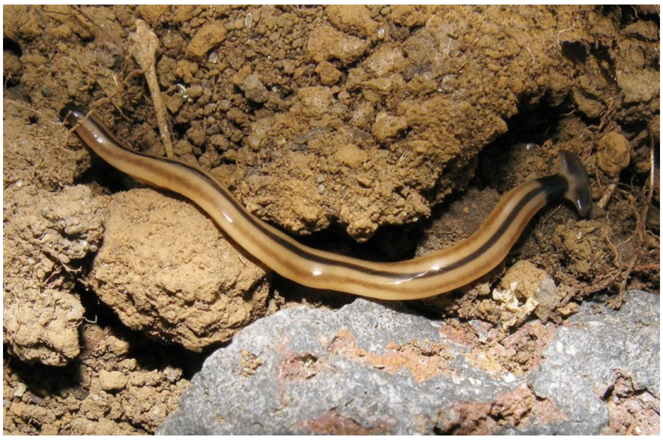

Bipalium vagum V33

#	Date	Locality	Department / State	Country - Continent	Origin
**V33**	17/08/2015	unknown	La Réunion	La Réunion - Africa	Lacoste, Marie

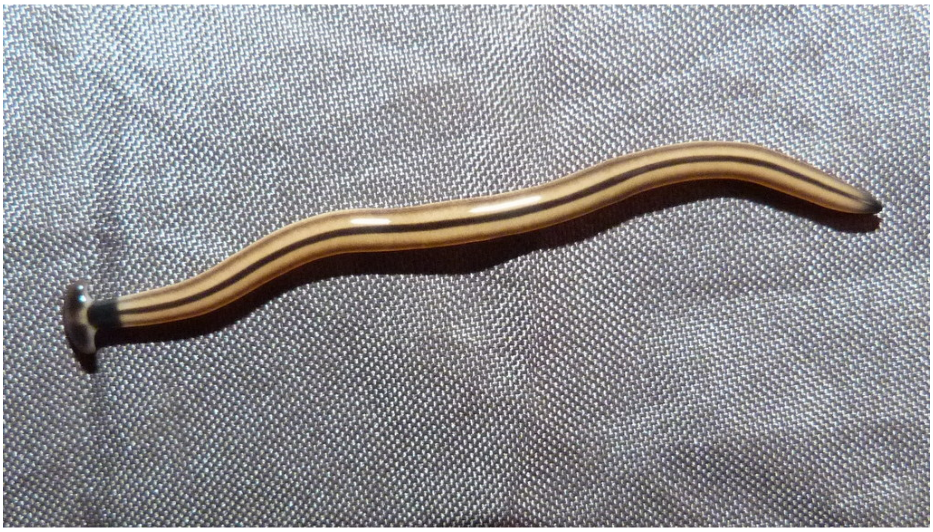

Supplement: Supplemental Information 2 — Photographs and details of specimens of bipaliines obtained from citizen science, without molecular data. Corresponds to Tables 5–8 of the paper. [file peerj-06-4672-s002.pdf]
