# Supplementary material for: Giant worms chez moi! Hammerhead flatworms (Platyhelminthes, Geoplanidae, Bipalium spp., Diversibipalium spp.) in metropolitan France and overseas French territories
Source: PeerJ. 2018 May 22;6:e4672. doi: 10.7717/peerj.4672 (PMC5969052; doi:10.7717/peerj.4672)
Supplement: Supplemental Information 3 [file peerj-06-4672-s003.pdf]

Des vers géants chez moi ! Plathelminthes (Plathelminthes, Geoplanidae, *Bipalium* spp., *Diversibipalium* spp.) en France métropolitaine et dans les territoires français d'outre-mer

Jean-Lou Justine [1*], Leigh Winsor [2], Delphine Gey [3], Pierre Gros [4] et Jessica Thévenot [5]

1 Institut Systématique, Évolution, Biodiversité (ISYEB), Muséum National d'Histoire Naturelle, CNRS, Sorbonne Université, EPHE, 57 rue Cuvier, CP 51, 75005 Paris, France

2 College of Science and Engineering, James Cook University, Townsville, Australia

3 Service de Systématique Moléculaire, Muséum National d'Histoire Naturelle, Paris, France

4 Amateur naturaliste, Cagnes-sur-Mer, France

5 Responsable « espèces exotiques envahissantes », UMS PatriNat (AFB, CNRS, MNHN), Muséum National d'Histoire Naturelle, Paris, France

Auteur de correspondance : Jean-Lou Justine, justine@mnhn.fr

Institut Systématique Évolution Biodiversité (ISYEB),

Muséum National d'Histoire Naturelle, CNRS, Sorbonne Université, EPHE,

57 rue Cuvier, CP 51, 75005 Paris, France

Traduction intégrale en français de :

Giant worms *chez moi!* Hammerhead flatworms (Platyhelminthes, Geoplanidae, *Bipalium* spp., *Diversibipalium* spp.) in metropolitan France and overseas French territories

PeerJ 6 : e4672 (2018)

DOI : 10.7717/peerj.4672

Résumé

Contexte. Les espèces des genres *Bipalium* et *Diversibipalium*, ou bipaliinés, sont des géants parmi les Plathelminthes terrestres (famille des Geoplanidae), atteignant une longueur de 1 m ; ils se distinguent facilement des autres vers plats par la forme caractéristique, en marteau, de leur tête. Les bipaliinés, qui ont leur origine dans les régions chaudes de l'Asie, sont des espèces envahissantes maintenant répandues dans le monde entier. Cependant, la littérature scientifique est très pauvre sur la répartition de ces espèces très répandues, et leur invasion dans les pays européens n'a pas été étudiée. **Méthodes.** Dans cet article, sur la base d'une enquête quadriennale basée sur les sciences participatives, qui a permis d'obtenir des observations de 1999 à 2017 et un total de 111 signalements, nous fournissons des informations sur les 5 espèces présentes en France métropolitaine et dans les territoires français d'outre-mer. Nous avons également étudié la variabilité moléculaire des séquences de la cytochrome-oxydase 1 (COI). **Résultats.** Trois espèces sont signalées en France métropolitaine : *Bipalium kewense*, *Diversibipalium multilineatum*, et une espèce *Diversibipalium* « noire » sans nom. Nous signalons également la présence de *B. kewense* dans des territoires d'outre-mer tels que la Polynésie française (Océanie), la Guyane française (Amérique du Sud), les Antilles françaises en Martinique, Guadeloupe, Saint Martin et Saint Barthélemy et aussi Montserrat (Amérique centrale), et à La Réunion (au large de l'Afrique du Sud-Est). Pour *B. vagum*, les observations incluent la Guyane française, la Guadeloupe, la Martinique, Saint Barthélemy, Saint Martin, Montserrat, La Réunion et la Floride (USA). Une nouvelle espèce probable, *Diversibipalium* sp. « bleu », est signalée de l'île de Mayotte (au large de l'Afrique du Sud-Est). *Bipalium kewense*, *B. vagum* et *D. multilineatum* ont chacun montré une variabilité de 0% de leurs séquences COI, quelle que soit leur origine, ce qui suggère que les spécimens sont clonaux et que la reproduction sexuée est probablement absente. Le barcode du COI a été efficace pour identifier les espèces, avec des différences de plus de 10% entre les espèces ; cela suggère que le barcode peut être utilisé à l'avenir pour identifier ces espèces envahissantes. Dans le sud-ouest de la France métropolitaine, une petite zone située dans le département des Pyrénées-Atlantiques s'est avérée être un point chaud de biodiversité et d'abondance des bipaliinés pendant plus de 20 ans, probablement en raison du climat local doux. **Discussion.** Les résultats actuels suggèrent fortement que les espèces présentes en France métropolitaine et dans les territoires d'outre-mer devraient être considérées comme des espèces exotiques envahissantes. Nos nombreux signalements en France métropolitaine soulèvent des questions : en tant que scientifiques, nous avons été étonnés que ces vers longs et colorés puissent échapper à l'attention des scientifiques et des autorités d'un pays développé européen pendant si longtemps. Une meilleure connaissance des Plathelminthes terrestres est certainement nécessaire.

Introduction

Les vers plats terrestres (Plathelminthes, Geoplanidae) sont des animaux prédateurs associés au sol. Bien que les petites espèces (généralement moins de 1 cm de longueur) telles que *Microplana* spp. ou *Rhynchodemus* spp. sont autochtones en Europe (Álvarez-Presas et al., 2012), les grandes espèces ne le sont pas. Les signalements des vers plats exotiques envahissants en Europe ces dernières années (Sluys 2016) incluent *Arthurdendyus triangulatus*, originaire de Nouvelle-Zélande, *Platydemus manokwari*, de Papouasie-Nouvelle-Guinée, *Obama nungara*, du Brésil et *Parakontikia ventrolineata*, *Caenoplana coerulea* et *Caenoplana bicolor*, d'Australie (voir **Tableau 1** pour les auteurs de taxons et les références clés). Toutes ces espèces sont des animaux bien visibles, de plusieurs centimètres de longueur. Encore plus grandes sont les espèces de *Bipalium* (et les genres proches), ou « vers plats à tête en marteau » : elles peuvent dépasser 20 centimètres (von Graff 1899) et une espèce atteint même une longueur de 1 mètre en élongation (Kawakatsu et al. 1982). Dans cet article, nous nous intéressons à ces espèces géantes et nous rapportons des nouvelles découvertes obtenues principalement par les sciences participatives en France métropolitaine et dans les territoires français d'outre-mer des Antilles (Guadeloupe, Martinique et Saint Barthélemy), en Amérique du Sud (Guyane française) et dans l'Océan Indien (La Réunion, Mayotte). Cinq espèces ont été trouvées, parmi lesquelles trois peuvent être attribuées à des taxons binomiaux connus (*Bipalium kewense*, *Bipalium vagum* et *Diversibipalium multilineatum*) et deux n'ont pas de nom.

Les Plathelminthes terrestres sont transportés entre les pays, et à l'intérieur des pays, par le transport des plantes. Winsor (1983a) a résumé les connaissances sur la répartition mondiale de *Bipalium kewense*, en compilant les mentions de l'espèce dans 39 territoires ; en 2004, l'espèce a été répertoriés dans 45 territoires (Winsor et al., 2004), puis signalée en Italie du Nord et péninsulaire, en Sardaigne et Sicile (Gremigni 2003), République Tchèque et Slovaquie (Košel 2002), Cuba (Morffe et al., 2016), Équateur (Wizen 2015) et Pakistan (M. Darley, communication personnelle à LW). Comme l'a commenté Sluys (2016) : « Presque chaque année, on trouve *B. kewense* dans de nouveaux endroits : par exemple, cette année (2016) l'espèce a été trouvée sur l'île de São Miguel aux Açores et sur l'île de São Tomé dans le golfe de Guinée ». Bien que ces signalements dans des petites îles éloignées soient importants pour notre connaissance de ces espèces envahissantes (et nous ajoutons, en effet, beaucoup de nouveaux signalements de ce type dans ce document), nous considérons que la

principale conclusion de cet article est que plusieurs espèces de vers plats sont établies, probablement depuis plus de 20 ans, dans un pays européen, la France. Cela met en évidence un aveuglement inattendu des scientifiques et des autorités face à une invasion par de grands animaux invasifs bien visibles.

L'identification des Plathelminthes terrestres à partir de spécimens ou de photographies est parfois un exercice futile, en l'absence d'étude anatomique détaillée. Dans cet article, nous avons testé l'identification avec des séquences de la cytochrome-oxydase 1 (COI). Nous confirmons que le barcode par COI est efficace pour les espèces étudiées ici ; de plus, notre étude de barcode a révélé que, dans chaque espèce, les spécimens ne présentaient aucune variabilité génétique, ce qui suggère qu'ils sont clonaux, sans reproduction sexuée.

Matériel et méthodes

Sciences participatives et collecte d'informations

En 2013, l'un d'entre nous (JLJ) a organisé en France un réseau de science participative pour la collecte d'informations sur les Plathelminthes terrestres. Cela comprenait un blog (http://bit.ly/Plathelminthe) et un compte Twitter (https://twitter.com/Plathelminthe4). Ces efforts ont été annoncés par les médias (radio, télévision et journaux).

Des rapports d'observation de Plathelminthes terrestres ont été reçus des citoyens, principalement par courriel, parfois par téléphone. Des photos et des détails sur la localité ont été sollicités, et seuls les rapports incluant cette information ont été pris en compte. Les signalements erronés (limaces, myriapodes, vers de terre, sangsues, chenilles, Nématomorphes et Némertes) ont été éliminés. Les informations collectées à partir des sciences participatives ont permis de suivre plusieurs Plathelminthes terrestres (Justine et al., 2014a). Les photographies ont été étudiées et les espèces ont été identifiées dans la mesure du possible. Seules les informations relatives aux bipaliinés sont rapportées dans cet article. Parfois, les citoyens ont fourni des documents datant d'avant l'enquête, comme un film amateur pris en 1999. La plupart des citoyens ont fourni une autorisation d'utiliser les photographies au moment du contact initial par courriel. Lorsque nous avons préparé ce document pour publication, nous avons demandé l'autorisation d'utiliser les photographies et de les publier sous une licence Creative Commons 4.0 ; seulement un des citoyens a refusé de fournir l'autorisation, mais certains d'entre eux n'ont pas répondu, probablement simplement

parce qu'ils ont changé leurs courriels ou ne les ont pas lus. Dans ces cas, nous fournissons des informations scientifiques sur la présence d'espèces, mais nous n'incluons pas la photographie du ver ou le nom du citoyen dans le document.

À l'origine, ces efforts visaient à recueillir des informations en provenance de la France métropolitaine, mais ils ont atteint de façon inattendue les territoires français sur d'autres continents et fourni des informations et des spécimens supplémentaires.

Collecte des spécimens

Dans certains cas, après examen de photographies, des spécimens ont été sollicités auprès des citoyens ayant signalé des observations ; ils ont été envoyés vivants ou dans l'éthanol par les citoyens, enregistrés dans les collections du Muséum National d'Histoire Naturelle à Paris (MNHN), et traités pour analyse moléculaire.

Lorsque les spécimens ont été obtenus vivants, ils ont été fixés dans de l'eau chaude puis conservés dans de l'éthanol à 95%. Dans certains cas, des spécimens ont également été fixés dans l'eau chaude et conservés dans une solution à 4% de formaldéhyde.

Les descriptions des espèces sont basées sur des spécimens et des photographies obtenus dans ce projet.

Séquences moléculaires

Pour l'analyse moléculaire, un petit morceau du corps (1-3 mm^3) a été prélevé sur le bord latéral des individus fixés à l'éthanol. L'ADN génomique a été extrait en utilisant le Mini Kit DNA QIAamp (Qiagen). Deux séries d'amorces ont été utilisées pour amplifier le gène COI. Un fragment de 424 pb (désigné dans ce texte par « séquence courte ») a été amplifié avec les amorces JB3 (= COI-ASmit1) (direct 5'-TTTTTGGGCATCCTGAGGTTTAT-3 ') et JB4.5 (= COI-ASmit2) (inverse 5'-TAAAGAAAGAACATAATGAAAATG-3') (Bowles et al., 1995, Littlewood et al., 1997). La réaction de PCR a été réalisée dans 20 µl, contenant 1 ng d'ADN, 1 x tampon de PCR CoralLoad, 3 Mm de MgCl2, 66 µM de chaque dNTP, 0,15 µM de chaque amorce et 0,5 unité d'ADN polymérase Taq (Qiagen). Le protocole d'amplification était : 4' à 94°C, suivi de 40 cycles de 94°C pendant 30", 48°C pendant 40", 72°C pendant 50", avec une extension finale à 72°C pendant 7'. Un fragment de 825 pb a été amplifié avec les amorces BarS (direct 5'-GTTATGCCTGTAATGATTG-3 ') (Álvarez-Presas et al., 2011) et COIR (inverse 5'-CCWGTYARMCCHCCWAYAGTAAA-3') (Lázaro et al.,

2009), selon Mateos et al. (2013). Les produits de PCR ont été purifiés et séquencés dans les deux directions sur un séquenceur à 96 capillaires DNA Analyzer 3730xl (Applied Biosystems). Les résultats des deux analyses ont été concaténés pour obtenir une séquence de COI de 909 pb (désignée dans ce texte par « séquence longue »). Les séquences ont été vérifiées en utilisant le logiciel CodonCode Aligner (CodonCode Corporation, Dedham, MA, USA), comparées au contenu de la base de données GenBank en utilisant BLAST et déposées dans GenBank sous le numéro d'accès MG655587-MG655618. Pour plusieurs échantillons, seules des séquences « courtes » ont été obtenues (**Tableau 2**).

Arbres et distances

MEGA7 (Kumar et al., 2016) a été utilisé pour estimer les distances génétiques (distance du paramètre kimura-2) et l'histoire évolutive a été déduite de la distance des paramètres kimura-2 en utilisant la méthode du Neighbor-Joining (Saitou & Nei 1987) ; toutes les positions de codons ont été utilisées, avec un bootstrap de 1000 répétitions. L'histoire évolutive a également été déduite en utilisant la méthode du maximum de vraisemblance (ML). Le meilleur modèle d'évolution des données, estimé par MEGA7 (Kumar et al., 2016) sous modèle bayésien (BIC), a été le modèle Hasegawa-Kishino-Yano (Hasegawa, Kishino et Yano, 1985) avec une distribution discrète de gamma et certains sites invariables (HKY + G + I). L'arbre ML a été calculé par MEGA7, avec un bootstrap de 100 réplications.

Note à propos de la taxonomie de *Diversibipalium*

La taxonomie basée sur la morphologie des Plathelminthes terrestres s'appuie sur une série de caractères, en particulier ceux qui sont fournis par l'anatomie interne, et surtout ceux du système reproducteur (Winsor et al., 1998). Les organes reproducteurs ne sont disponibles que dans les spécimens sexuellement mûrs et nécessitent des préparations histologiques approfondies pour leur description. Malheureusement, de nombreuses espèces de Plathelminthes terrestres ont été décrites uniquement à partir de la morphologie externe. Certaines espèces se reproduisent uniquement asexuellement (scissiparité) et ne montrent donc pas d'organes mûrs ; c'est notamment le cas de certaines espèces envahissantes lorsqu'elles ne sont pas dans leur région d'origine. Cependant, les bipaliinés représentent un cas particulier car la morphologie externe, c'est-à-dire la présence d'une tête « en marteau », est caractéristique de la sous-famille, qui peut donc être facilement différenciée si une photographie de la tête est disponible. Le genre *Diversibipalium* Kawakatsu et al., 2002 est un groupe collectif créé pour accueillir temporairement des espèces de la sous-famille des

Bipaliinae dont l'anatomie de l'appareil de copulation est encore inconnue (Kawakatsu et al., 2002). Pour cette raison, nous attribuons nos deux espèces non décrites, « noire » et « bleue » à ce genre. Nous insistons sur le fait que l'attribution d'espèces au genre *Diversibipalium* ne signifie pas que ces espèces ont des caractères en commun : la seule caractéristique qu'elles partagent est notre ignorance de leur anatomie interne. Ces deux espèces seront examinées histologiquement et décrites en détails par les auteurs, ultérieurement.

Résultats

Collectes d'information par les sciences participatives

Après la découverte initiale en juin 2013 de deux espèces de Plathelminthes terrestres dans son jardin par Pierre Gros, entomologiste amateur et photographe, plus de 600 signalements ont été reçus sur 4 ans (juin 2013-septembre 2017). La plupart des signalements provenaient de citoyens, d'autres de scientifiques ou autres professionnels. De façon inattendue, ces signalements ont inclus des mentions de plus de 8 espèces de Plathelminthes terrestres (Justine et al., 2014a), le plus récent étant *Marionfyfea adventor*. Parmi ceux-ci, 111 signalements concernaient des bipaliinés. La **Figure 1** est une carte de ces signalements en France métropolitaine.

Les résultats sont présentés comme suit : après une évaluation de l'identification des spécimens basée sur la morphologie et les molécules, des paragraphes distincts fournissent, pour chaque espèce, une brève description et son aire de répartition en France métropolitaine et d'outre-mer, basées sur les spécimens et les photographies obtenus grâce aux sciences participatives.

Identification moléculaire des spécimens échantillonnés

Des séquences ont été obtenues à partir de spécimens appartenant à cinq espèces (**Tableau 2**), dont trois espèces nommées, *Bipalium kewense* (spécimens provenant de 13 localités, 17 séquences y compris réplicats), *Diversibipalium multilineatum* (spécimens de 4 localités, 8 séquences incluant réplicats), *Bipalium vagum* (spécimens de 3 localités, 5 séquences y compris réplicats) et deux espèces non nommées, *Diversibipalium* « noir » (1 spécimen de 1 localité, 1 séquence) et *Diversibipalium* « bleu » (spécimens de 2 localités, 6 séquences y compris réplicats).

Un arbre (**Figure 2**) a été construit à partir d'une analyse de nos nouvelles séquences de COI et de séquences de GenBank. Les deux arbres NJ et ML ont montré des topologies comparables, mais les valeurs bootstrap des branches, dans les deux arbres, ont été contrastées : 100% pour toutes les branches représentant des espèces, et très faibles pour les nœuds supérieurs. Nous avons donc considéré que les arbres étaient informatifs pour montrer l'identité génétique de tous les spécimens d'une espèce, mais pas pour inférer les relations entre les taxons. Ainsi, aucun autre commentaire sur les relations interspécifiques n'est donné dans le reste de ce texte ; en ce sens nous suivons les principes généraux du barcode par COI (Hebert et Gregory 2005) : « nous soulignons que les barcodes par ADN ne visent pas à rétablir les relations phylogénétiques ; ils cherchent plutôt à identifier les espèces connues et à aider à la découverte de nouvelles ». Nous avons remarqué, mais ne commentons pas, l'identification erronée probable de certaines séquences déposées dans GenBank, comme *Novibipalium venosum* ou « *D. multilineatum* » HM346600.

Chacune des trois espèces nommées appartenait à un clade avec un soutien par bootstrap élevé (100%) (**Figure 2**).

Pour *Bipalium kewense*, le clade comprend des séquences GenBank d'Espagne, des Açores et de Cuba ; nos 13 nouvelles séquences (hors réplicats) proviennent de 7 localités en France métropolitaine, 3 territoires français d'outre-mer (Guadeloupe, Martinique, Guyane française) et 2 autres pays, Monaco et Portugal. Toutes les séquences de COI étaient strictement identiques.

Pour *Diversibipalium multilineatum*, le clade comprend des séquences GenBank d'Italie et de France (séquence du spécimen MNHN JL177, déjà publié par Mazza et al., 2016), et nos 6 nouvelles séquences (hors réplicats) proviennent de 3 localités en France métropolitaine. Toutes les séquences COI étaient strictement identiques.

Pour *Bipalium vagum*, aucune séquence n'a été trouvée dans GenBank. Nos 5 nouvelles séquences proviennent d'un territoire français d'outre-mer (Guadeloupe) et 2 autres pays, Montserrat (Antilles) et Floride, USA. Toutes les séquences de COI étaient strictement identiques.

Pour *Diversibipalium* « noir » de France métropolitaine et *Diversibipalium* « bleu » de Mayotte, chacune des séquences n'avait aucune correspondance dans les séquences GenBank

ou nos nouvelles séquences, suggérant qu'elles appartiennent à une espèce qui n'a jamais été séquencée pour le gène de COI.

Distances entre les taxons

Des séquences « courtes » ont été obtenues à partir de tous les spécimens et des séquences « longues » ont été obtenues à partir de seulement quelques-uns d'entre eux. Les distances entre les espèces de bipaliinés ont été calculées à partir de deux séries de séquences, les séquences « courtes » et les séquences « longues ».

Le premier ensemble comprenait des séquences « courtes » et 7 taxons de bipaliinés étaient disponibles. Les distances variaient de 10,9% à 21,2% (**Tableau 3**). Les taxons les plus proches étaient *B. kewense* - *D. multilineatum* avec une distance interspécifique de 10,9%, et les plus éloignés étaient *Diversibipalium* « bleu » et *B. adventitium* avec 21,2%.

Le deuxième ensemble comprenait uniquement des séquences « longues » et 4 taxons de bipaliinés étaient disponibles. Les distances étaient plus élevées qu'avec des séquences courtes et variaient de 15,9% à 25,9% (**Tableau 4**). Les taxons les plus proches étaient, là aussi, *B. kewense* - *D. multilineatum* avec une distance interspécifique de 15,9%, et les plus éloignés étaient *Diversibipalium* « bleu » et *D. multilineatum* avec 25,9%.

Morphologie, taxonomie et répartition

Bipalium kewense Moseley, 1878

Morphologie et patron de couleurs (Figures 3-9)

Les spécimens vivants sont longs et minces et ont une longueur comprise entre 100 mm et 270 mm (**Tableau 5**). Les spécimens conservés à partir desquels les résultats COI ont été obtenus mesuraient 170 mm (MNHN JL224), 120 mm (MNHN JL308) et 65 mm (MNHN JL270) avec un rapport distance de la bouche/ longueur du corps de 41,2%, 41,7% et 32,3% respectivement. Aucun des spécimens conservés examinés n'avait de gonopore et ils sont donc considérés comme non sexuels. L'extrémité antérieure est élargie en une plaque frontale en forme de demi-lune transversale avec des bords recourbés (falciforme). La couleur dorsale générale est habituellement un ocre clair (**Figure 3**), avec cinq bandes longitudinales de couleur noire à grise : une bande médiane, une paire latérale et des bandes marginales paires qui commencent à la base de la plaque frontale, là où elle joint le corps et le « cou ». La

plaque dorsale (**Figures 4, 5**) est généralement de la même couleur que le corps, ou légèrement plus foncée, avec des bords postérieurs recourbés. La bande médiane est noire, étroite, avec des bords nets, s'étendant caudalement à partir du bas du cou sur toute la longueur du corps, et est plus large sur la zone pharyngée. Des bandes latérales paires, de couleur brun foncé à brun pâle, avec des bords diffus, constantes sur toute la longueur du corps, sont séparées des bandes médiane et marginale par une largeur égale de couleur de fond. Les bandes noires paires, fines et marginales, aux bords nets, s'étendent sur toute la longueur du corps. Les bandes latérales et marginales paires s'unissent juste en arrière du cou pour former une bande transversale noire incomplète sur le cou, interrompue dorsalement par un petit espace médian, et ventralement par la sole. La plaque frontale, ventralement, est de couleur grisâtre avec une marge ocre clair. La surface ventrale (**Figure 6**) est de couleur ocre clair, avec une sole rampante distincte et blanc cassé, délimitée par des rayures gris-violet diffuses, étroites, longitudinales et paires, commençant à la terminaison ventrale du collier et s'étendant toute la longueur du corps. Dans la **Figure 7**, nous montrons des preuves de prédation sur un ver de terre autochtone européen non identifié, et sur les **Figures 8-9** des preuves de la reproduction par scissiparité où le fragment détaché est immédiatement mobile mais ne possède pas la tête caractéristique en forme de marteau.

Différenciation des autres espèces

Les spécimens de *B. kewense* qui nous ont été envoyés, ou pour lesquels nous n'avons reçu que des photographies, correspondaient aux descriptions morphologiques publiées de l'espèce (Winsor 1983a). *Bipalium kewense* se différencie extérieurement des espèces rayées similaires par la bande transversale noire incomplète au niveau du cou (le « collier »), la fine bande longitudinale médiane dorsale qui débute au niveau ou au-dessous de la bande transversale du cou, l'allure et la forme des rayures dorsales et ventrales, et la position relative des ouvertures corporelles (Winsor 1983a).

Signalements obtenus par les sciences participatives

Nous avons obtenu 50 signalements de *B. kewense*, dont 14 confirmés par des molécules (**Tableau 2**) et 36 par des photographies seulement (**Tableau 6**). Les localités où des bipaliinés ont été trouvés dans le milieu extérieur, généralement dans des jardins, sont au Portugal (1 signalement), en Martinique (3), Guadeloupe (6), Guyane française (1), Polynésie française (1), La Réunion (1), Monaco (1), soit 7 territoires répartis sur 5 continents (Europe,

Amérique du Nord, Amérique du Sud, Afrique, Océanie) et 36 en France métropolitaine (**Figure 1**), dans 9 départements : Corse-Sud (Corse) (2), Var (2), Gironde (1), Loire-Atlantique (1), Landes (3), Alpes-Maritimes (5), Yonne (2), Hautes-Pyrénées (1) et Pyrénées-Atlantiques (16). En outre, nous avons reçu deux rapports dans des serres dans le département de l'Yonne. Parmi les 34 signalements en France métropolitaine, 16, soit plus de la moitié, provenaient du département des Pyrénées-Atlantiques (**Tableaux 2 et 6**). La répartition de nos signalements est illustrée dans la **Figure 1** pour la France métropolitaine (Corse comprise). Les dates des signalements sont réparties de 1999 à 2017 ; le signalement le plus ancien (1999) était dans les Pyrénées-Atlantiques.

Résultats moléculaires

Les séquences de COI étaient strictement identiques pour les spécimens de toutes les localités où des échantillons ont été séquencés.

Diversibipalium multilineatum (Makino et Shirasawa, 1982)

Morphologie et patron de couleurs (Figures 10-14)

Les spécimens vivants mesuraient entre 150 mm (MNHN JL177) et 210 mm (MNHN JL059). Les spécimens conservés représentatifs à partir desquels les résultats COI ont été obtenus mesuraient 85 mm (MNHN JL210), 65 mm (MNHN JL161A) et 60 mm (MNHN JL142A) de longueur (**Tableau 5**), avec un ratio distance de la bouche / longueur du corps de 29,4%, 38,5% et 41,7% respectivement. Aucun des spécimens examinés n'avait de gonopore et ils sont donc considérés comme non sexuels. Le corps est allongé (**Figure 10**) avec l'extrémité antérieure élargie en une plaque frontale de forme semi-lunaire transversalement avec des bords arrondis (**Figures 11-13**). Immédiatement en arrière de la tête, le corps se rétrécit pour former un « cou », puis s'élargit progressivement jusqu'à la largeur maximale sur la région pharyngienne, puis se rétrécit légèrement jusqu'à une extrémité postérieure arrondie. La couleur générale de la face dorsale, y compris la plaque frontale, est généralement un brun ocre clair avec cinq bandes longitudinales espacées uniformément, de couleur noire à brun foncé : une bande médiane, une paire de bandes latérales et une paire de bandes marginales. La bande médiane est noire et étroite avec des marges nettes. Elle a une forme lenticulaire caractéristique prononcée commençant au tiers antérieur de la plaque frontale, puis se rétrécissant en une fine bande foncée s'étendant vers la queue sur toute la longueur du corps,

plus large sur la zone pharyngée. De chaque côté de la bande médiane, chacune séparée par une largeur égale de couleur de fond, se trouvent une bande latérale et une bande submarginale qui se rejoignent au niveau du cou dans la courbure interne de la tête au niveau du « cou » et s'étendent sur toute la longueur du corps. Les bandes latérales sont de couleur noire à brun foncé avec des marges diffuses, d'environ 2-3 fois la largeur de la bande médiane ; les bandes marginales paires sont marron, étroites, et ont à peu près la même épaisseur que la bande médiane. La surface ventrale (**Figure 14**) est d'un ocre brun clair, généralement un peu plus pâle que le dos, avec une sole rampante blanche distincte, délimitée par des bandes longitudinales paires, marron et étroites, commençant à la terminaison ventrale du collier, et se prolongeant sur l'ensemble la longueur du corps. Une bande sombre mi-ventrale foncée, généralement discontinue, s'étend de la base de la plaque frontale à l'extrémité postérieure.

Différenciation des autres espèces

Les spécimens de *D. multilineatum* qui nous ont été envoyés ou pour lesquels nous n'avons reçu que des photographies correspondaient à la description morphologique publiée de l'espèce (Makino et Shirasawa 1983, Mazza et al., 2016). *Diversibipalium multilineatum* se différencie extérieurement des espèces à rayures similaires par la présence d'un début lenticulaire de la bande médiane sur la plaque frontale, la présence de bandes ventrales médianes distinctes, la bande longitudinale mi-ventrale mince, sombre et généralement incomplète, et la position relative de la bouche.

Signalements obtenus par les sciences participatives

Nous avons obtenu un total de 19 signalements. Un signalement provenait de Suisse et 16 d'endroits dans le milieu extérieur en France métropolitaine, dans les départements de l'Ariège (1), de la Haute-Garonne (3), de l'Isère (2), des Landes (2), du Val d'Oise (2) et des Pyrénées-Atlantiques (6) ; un signalement a été confirmé deux années de suite (2014-2015) dans le même jardin de Bellocq (Pyrénées-Atlantiques). En outre, deux signalements provenaient de serres, dans le département du Lot (1) et dans le Val d'Oise (1). Parmi les 16 signalements dans le milieu extérieur en France métropolitaine, plus d'un tiers (6) vient du département des Pyrénées-Atlantiques. La répartition de nos signalements est présentée à la **Figure 5** pour la France métropolitaine (Corse comprise). Les dates des signalements allaient

de 2010 à 2017 ; le signalement le plus ancien (2010) était dans les Pyrénées-Atlantiques (**Tableaux 2, 7**).

Résultats moléculaires

Comme pour *B. kewense*, les séquences COI de *D. multilineatum* étaient strictement identiques dans les spécimens de toutes les localités.

Bipalium vagum Jones et Sterrer, 2005

Morphologie et patron de couleurs (Figures 15-18)

Les spécimens vivants sont de taille moyenne, l'un mesurant environ 36 mm (**Tableau 5**, observation V04, à partir d'une photo avec échelle). Les échantillons conservés, à partir desquels les résultats COI ont été obtenus, mesuraient respectivement 27,5 mm (MNHN JL164), 25,6 mm (MNHN JL163) et 15 mm (MNHN JL307) avec un ratio distance de la bouche / longueur du corps 60,7%, 50,4% et 49% et un ratio distance du gonopore / longueur du corps de 70,7% (MNHN JL163) et 72% (MNHN JL307).

La couleur dorsale générale est brun pâle, avec trois bandes longitudinales dorsales noires à brunes : une large bande médiane nettement délimitée noire et deux bandes latérales brun foncé, moins nettement délimitées, le tout commençant au niveau de la bande transversale du cou, continuant sur toute la longueur du corps, et se terminant souvent par une extrémité noire bien définie. Les bandes longitudinales sont séparées les unes des autres par une largeur égale de couleur de fond (**Figures 15-18**).

Différenciation des autres espèces

Les spécimens de *B. vagum* qui nous ont été envoyés, ou pour lesquels nous n'avons reçu que des photographies, correspondaient à la description morphologique publiée de l'espèce (Jones & Sterrer 2005). *Bipalium vagum* se distingue extérieurement des espèces de morphologie similaire par une combinaison des caractères, en particulier sa taille relativement petite, la bande de cou transversale qui est continue dorsalement, d'où vient la large bande médiane noire, et la position relative des ouvertures du corps.

Signalements obtenus par les sciences participatives

Aucun signalement n'a été obtenu de France métropolitaine. Nous avons obtenu 39 signalements **(Tableaux 2, 8**), tous dans le milieu extérieur, de Guyane française (4 signalements) et de 5 îles des Antilles, dont Montserrat (1) et 4 territoires français, à savoir la Guadeloupe (10), la Martinique **(**3), Saint Barthélemy (2) et Saint Martin (1), et de l'île de la Réunion dans l'océan Indien (15) ; des spécimens de Floride, aux États-Unis, ont également été séquencés. Malheureusement, malgré les nombreux documents photographiques de La Réunion, aucun spécimen n'a été reçu pour le séquençage, mais la morphologie et le patron de couleur étaient similaires à ceux des autres localités (**Figures 15-18**). Les dates des signalements allaient de 2005 à 2017, et le plus ancien (2005) venait de Guyane française (**Tableaux 2, 7**).

Résultats moléculaires

Les séquences COI étaient strictement identiques pour les spécimens de toutes les localités.

Diversibipalium sp. « noir » de France métropolitaine

Morphologie et patron de couleurs (Figures 19-21)

Le spécimen vivant atteint une longueur de 20 à 25 mm. Un spécimen sexuel préservé (MNHN JL090) mesure 20 mm de long et 3,2 mm de large, la bouche étant ventralement à 6 mm (rapport distance de la bouche/ longueur du corps de 30%) et le gonopore à 7,8 mm (rapport distance du gonopore/ longueur du corps de 39%).

La couleur générale dorsale de ce petit Plathelminthe est noire, sans aucune trace de rayures dorsales (**Figures 19-21**). La surface ventrale est de couleur gris clair avec une sole rampante plus pâle.

Différenciation des autres espèces

En l'absence de données détaillées dans la littérature, il est actuellement difficile de déterminer si *Diversibipalium* sp. 1 « noir » est une nouvelle espèce, ou l'une des petites espèces noires de *Diversibipalium* telles que *Diversibipalium* sp. « Kuanmoto » de Kawakatsu et al. (2005).

Origine possible de cette espèce

Nous ne proposons aucune hypothèse concernant l'origine géographique de cette espèce, à part le fait qu'elle n'est évidemment pas européenne, puisqu'aucun bipaliiné n'est connu de ce continent.

Résultats moléculaires

Le barcode COI de cet échantillon est clairement différent de toutes les autres séquences connues. Nous pouvons affirmer sans risque que cette espèce n'a jamais été séquencée auparavant. Il n'est pas facile de savoir si l'espèce a déjà été décrite ou non et cela nécessiterait l'examen de spécimens mûrs.

Diversibipalium sp. « bleu » de Mayotte (Océan Indien)

Morphologie et patron de couleurs (Figures 22-26)

Les spécimens vivants atteignent une longueur de 45 mm (**Figure 22**). Le spécimen sexuel préservé a une longueur de 9 mm et une largeur de 1 mm, la bouche étant située à environ 3,5 mm de l'extrémité antérieure (rapport distance de la bouche/ longueur du corps 39%) et le gonopore à 6,5 mm de l'extrémité antérieure (rapport distance du gonopore/ longueur du corps 72,2%).

La plaque frontale de ce beau petit Plathelminthe est d'une couleur brun rouille qui s'étend jusqu'à des taches irrégulières sur le « cou ». La couleur de fond dorsale est un bleu-vert iridescent (« turquoise foncé à paillettes »), et la surface ventrale est d'une couleur brun très pâle, avec la sole rampante blanche à vert pâle. L'irisation et la couleur bleu-vert sont perdues lors de la fixation, laissant une couleur de fond brun foncé (**Figures 22-26**).

Différenciation des autres espèces

Il n'existe aucune mention d'un Plathelminthe bipaliiné avec cette morphologie.

Origine possible de cette espèce

Mayotte et les Comores sont de petites îles volcaniques qui ont connu un intense commerce humain depuis des siècles avec les îles voisines et Madagascar et des territoires plus lointains dont l'Asie. N'importe lequel de ces territoires pourrait être le lieu d'origine de cette espèce.

Signalements obtenus par les sciences participatives

Nous avons obtenu des signalements de cette espèce uniquement de Mayotte, de la part de deux observateurs indépendants, l'un qui a fourni des spécimens et des photographies et l'autre qui n'a fourni que des photographies (**Tableaux 2, 9**).

Résultats moléculaires

Le barcode COI de cet échantillon est clairement différent de toutes les autres séquences connues. Nous pouvons affirmer sans risque que cette espèce n'a jamais été séquencée auparavant. Il n'est pas facile de savoir si l'espèce a déjà été décrite ou non.

Discussion

Validité du COI pour le barcode des Plathelminthes bipaliinés

Le barcode basé sur les séquences du gène mitochondrial de la cytochrome c oxydase I (COI) a été proposé comme solution au problème de l'identification des espèces (Hebert et al., 2003). Les barcodes basés sur les COI se sont avérés efficaces dans divers groupes, notamment les papillons (Lepidoptera) (Hebert et al., 2003) ou les poissons (Ward et al., 2005). Chez les vers plats (Plathelminthes), bien que le barcode basé sur les séquences COI ne soit pas le meilleur choix pour certains groupes (Vanhove et al., 2013), des études récentes ont montré qu'il permet de différencier efficacement des espèces de monogènes (Ayadi et al., 2017, Chaabane et al., 2016) et divers Triclades (Álvarez-Presas & Riutort 2014), y compris les Plathelminthes terrestres (famille Geoplanidae) (Álvarez-Presas et al 2011, 2012, 2014).

La présente étude montre que les séquences courtes de COI, facilement obtenues à partir de presque tous les échantillons, ont des distances interspécifiques de 10,9 à 21,2% (**Tableau 3**). Ces distances interspécifiques sont suffisamment élevées pour différencier les espèces de bipaliinés, en particulier en l'absence de variation intraspécifique. Les séquences longues fournissent des distances interspécifiques encore plus élevées, allant de 15,9 à 25,9% (**Tableau 4**), mais celles-ci sont moins faciles à obtenir et la base de données ne comprend que quatre espèces. Bien sûr, on pourrait objecter que la base de données actuelle (7 espèces avec des séquences courtes) est extrêmement limitée par rapport au nombre d'espèces décrites chez les bipaliinés - plus de 160 (Winsor 1983a). Cependant, la base de données actuelle

comprend les espèces les plus envahissantes au monde, les distances interspécifiques sont élevées et la variation intraspécifique est presque inexistante pour la plupart des espèces. Pour ces raisons, nous croyons que l'identification des espèces communes des vers plats bipaliinés peut être effectuée de manière fiable à partir des barcodes COI. Le barcode peut se faire à partir d'un ver très petit, immature, ou même d'un fragment. De plus, les barcodes COI peuvent probablement alerter les scientifiques sur la présence d'espèces non préalablement séquencées, si une séquence différente de celles rapportées dans la présente étude est trouvée.

Le fait que certaines bipaliinés ne se reproduisent pas sexuellement en dehors de leur habitat naturel ou de leur climat tropical ou subtropical, mais seulement par scissiparité (Winsor 1983a), est probablement une raison expliquant pourquoi aucune variabilité n'a été trouvée dans les spécimens, puisque les spécimens sont des clones, et que peu de mutations peuvent se produire. Cependant, cette explication n'est pas suffisante, car plusieurs populations d'origines diverses, chacune se clonant elle-même, pourraient être présentes dans le monde. Par opposition, chez *Platydemus manokwari*, les séquences COI ont démontré l'existence d'au moins deux haplotypes dans le monde, correspondant probablement à deux populations et à différentes voies d'invasion du monde (Justine et al., 2015). Nos données actuelles sur les bipaliinés suggèrent qu'une population est à l'origine de l'invasion pour chaque espèce. Ceci est particulièrement frappant pour *B. kewense*, avec des données moléculaires identiques provenant de plusieurs continents.

Persistance de *Bipalium kewense* et *Diversibipalium multilineatum* dans le milieu extérieur en France métropolitaine

Bipalium kewense a été décrit à l'origine à partir de spécimens dans l'une des serres de Kew, au Royaume-Uni (Moseley, 1878). Originaire d'une zone allant du Vietnam au Cambodge, l'espèce est actuellement cosmopolite (Winsor 1983a). Cependant, les distinctions sont importantes entre une espèce qui se trouve uniquement dans des constructions protégées et restreintes telles que les serres, et les espèces qui peuvent librement vivre et se reproduire à l'air libre dans le milieu extérieur. Il est clair que *B. kewense* est une espèce envahissante à l'extérieur dans les pays aux climats semi-tropicaux humides ou tropicaux humides et semble se limiter aux habitats anthropogéniquement modifiés ; c'est le cas dans les Caraïbes, comme la Guadeloupe ou la Martinique, d'où nous avons obtenu des spécimens. Cependant, jusqu'à récemment (Justine et al., 2014b), on considérait que *B. kewense*, en Europe, n'était confiné qu'à des serres chaudes et n'était donc pas une espèce envahissante. L'examen de la littérature

et de l'information obtenue par les sciences participatives (**Figure 1**) prouve maintenant le contraire.

En France, la présence de *B. kewense* en plein air a été signalée à Orthez et Bayonne en 2005 (Vivant, 2005). Grâce aux sciences participatives, nous avons obtenu une vidéo du ver, filmé dans la localité voisine d'Urcuit en 1999. De plus, nous avons obtenu des informations sur la présence de l'espèce à Arthez de Béarn, Hasparren, Villefranque, Urt (2014), près de Jurançon (2016), Nay (2016), Saint Jean de Luz (2016), Billère et Ustaritz (2017) et, comme dans l'article de Vivant, à Bayonne et Orthez (2014). Nous avons obtenu des spécimens de Saint-Pée-sur-Nivelle (2013), Ustaritz (2014), Bassussary (2014) et Orthez (2014). Toutes ces localités se trouvent dans le département des Pyrénées-Atlantiques, et nous avons également trois signalements du département des Landes, au nord des Pyrénées-Atlantiques, le long de la côte atlantique, dont Mimbastes (2014, avec information moléculaire), Hagetmau (2008) et Biscarosse (2014) et un signalement du département des Hautes-Pyrénées, plus éloigné de la côte, à Peyrouse (2017) (**Tableaux 2, 6**). La remarque de Vivant (2005) selon laquelle l'animal a été collecté « cinq fois au cours des 20 dernières années », le signalement de 1999, et les récents signalements et spécimens dans la même localité (Orthez) en 2014 suggèrent fortement que l'espèce est maintenant établie à Orthez et dans plusieurs localités du département des Pyrénées-Atlantiques (**Figure 27**). Une hypothèse alternative serait qu'une seule pépinière près de Bayonne serve de réservoir permanent de Plathelminthes et que tous ces signalements soient en fait des spécimens échappés des plantes récemment achetées, mais qui sont ensuite morts après avoir été libérés en extérieur ; cette hypothèse est réfutée par des signalements sur plusieurs années dans des localités similaires. Récemment, une personne de Billère (Pyrénées-Atlantiques) nous a envoyé des signalements répétés dans le même jardin en septembre et décembre 2017 et en janvier 2018, montrant clairement de nombreux spécimens vivants en plein air, même en hiver ; ils ont été trouvés à diverses profondeurs sous la surface du sol en janvier, ce qui est clairement un moyen pour l'espèce de survivre à la saison froide.

Nous notons que tous nos signalements proviennent de jardins et qu'aucun d'entre eux ne provenait d'endroits éloignés de la présence humaine ; cela peut être attendu des données scientifiques des sciences participatives.

Nous commenterons brièvement le climat de cette région. Le département des Pyrénées-Atlantiques est le département le plus méridional de la côte atlantique de la France ;

il comprend une région montagneuse et une région de basse altitude le long de l'océan. Cette dernière a un climat atlantique. Au sein du département, nous notons que la plupart des signalements (Nay, Urcuit, Urt, Saint-Jean-de-Luz, Saint-Pée-sur-Nivelle, Ustaritz et Bassussary) proviennent d'une petite région autour de Bayonne, le long de la côte atlantique (**Figure 27**). Le principal facteur limitant pour une espèce tropicale en Europe est, bien sûr, les basses températures. Pour un Plathelminthe sensible à la sécheresse et au gel, le nombre de jours de sécheresse en été et le nombre de jours de gel en hiver sont également des facteurs limitants importants. Des relevés météorologiques détaillés sont disponibles pour Biarritz, une localité proche de Bayonne (Infoclimat 2017) : la température moyenne annuelle est de 13,7°C, les précipitations annuelles de 1483 mm, même les mois secs (juillet et août) montrent une moyenne de 9-10 jours avec de la pluie, et le nombre de jours avec une température inférieure à -5°C est seulement de 1,5j/an. Cela suggère que cette région est particulièrement adaptée aux Plathelminthes terrestres. D'autres localités du sud de la France, comme les départements du Var et des Alpes-Maritimes, et la Corse, toutes deux en climat méditerranéen, ont des températures plus élevées et pourraient donc mieux convenir aux espèces tropicales, mais elles connaissent de plus longues périodes de sécheresse (Infoclimat 2017).

Fait intéressant, un signalement de *Diversibipalium multilineatum* provient également du même département, à Bellocq (avec des signalements sur deux ans), et le seul signalement de *Diversibipalium* sp. « noir » est également du même département, à Saint-Pée-sur-Nivelle, dans un jardin où *B. kewense* est également présent. Parmi les autres Plathelminthes terrestres envahissants trouvés dans les Pyrénées-Atlantiques figurent *Obama nungara*, *Caenoplana bicolor* et *Parakontikia ventrolineata* (données des sciences participatives). Avec un total de six espèces de Plathelminthes terrestres invasifs, le département des Pyrénées-Atlantiques est clairement un point chaud de la diversité et un petit paradis pour les Plathelminthes envahissants.

Pour *Diversibipalium multilineatum*, nous avons également deux signalements dans les mêmes jardins pendant deux années consécutives (**Tableau 7**). Ceci suggère que cette espèce est également établie dans le milieu extérieur en France métropolitaine, mais le nombre total de signalements est plus faible (16 contre 34 pour *B. kewense*). L'un des signalements concernait des centaines d'animaux.

Une évaluation plus détaillée des données écoclimatiques et autres concernant la répartition des Plathelminthes envahissants en France et dans les territoires français dépasse le cadre de cet article.

Les Plathelminthes terrestres bipaliinés se qualifient-ils comme espèces invasives en France métropolitaine ?

Nous avons reçu plusieurs rapports de citoyens mentionnant des douzaines de spécimens dans leurs jardins (**fichiers supplémentaires 1 et 2**) ; dans certains cas, les citoyens ont signalé à plusieurs reprises des nombres élevés, même lorsque les vers ont été enlevés à la main et détruits. De tels rapports justifient que l'espèce soit considérée comme « envahissante » dans le sens commun du terme.

Cependant, le terme « espèce invasive » a une signification plus précise en science. Les espèces exotiques envahissantes (EEE) sont définies par la Convention sur la Diversité Biologique et l'Union Internationale pour la Conservation de la Nature comme des « espèces dont l'introduction et / ou la propagation en dehors de leur distribution naturelle passée ou présente menace la diversité biologique » (Convention sur la Diversité Biologique, 2018 ; Union Internationale pour la Conservation de la Nature, 2018). Des définitions juridiques sont également disponibles dans divers pays. Pour les États-Unis, le décret 13112) (Executive Order 13112, 1999) définit une espèce envahissante comme « une espèce exotique dont l'introduction cause ou est susceptible de causer des dommages économiques ou environnementaux ou de nuire à la santé humaine ». En Europe, l'Institut pour la Politique Environnementale Européenne (Kettunen et al., 2009) utilise la définition suivante : « Les espèces exotiques envahissantes (EEE) sont des espèces non indigènes dont l'introduction et / ou la dissémination en dehors de leur aire de répartition présente une menace pour la biodiversité ». Le texte juridique le plus récent (Parlement européen, 2014) se lit comme suit (quelques parties ont été supprimées pour simplification) : « (1) L'apparition d'espèces exotiques, que ce soit d'animaux, de plantes, de champignons ou de micro-organismes, dans de nouveaux endroits n'est pas toujours une source de préoccupation. Cependant, un sous-ensemble significatif d'espèces exotiques peut devenir envahissantes et avoir un impact négatif sérieux sur la biodiversité et les services écosystémiques associés, ainsi que d'autres impacts sociaux et économiques, qui devraient être évités. [...] (2) Les espèces exotiques envahissantes représentent l'une des principales menaces pour la biodiversité et les services écosystémiques connexes. [...] (3) La menace pour la biodiversité et les services

écosystémiques connexes que posent les espèces exotiques envahissantes prend différentes formes, notamment les impacts graves sur les espèces indigènes et la structure et le fonctionnement des écosystèmes par altération des habitats, prédation, compétition, transmission des maladies, le remplacement des espèces indigènes dans une proportion significative de l'aire de répartition et par des effets génétiques par hybridation ».

Selon ces définitions, les bipaliinés trouvés dans les jardins en France métropolitaine et dans d'autres localités mentionnées dans ce document devraient clairement être considérées comme des espèces exotiques envahissantes, parce que les bipaliinés sont des prédateurs et, en tant que tels, menacent la faune du sol. En l'absence d'études écologiques détaillées, nous ne pouvons pas estimer l'impact exact de ces vers sur la faune ; la très grande taille des Plathelminthes bipaliinés, qui en font les plus grands prédateurs terrestres d'invertébrés, suggère que cet impact n'est pas négligeable (Zaborski, 2002).

Une classification précise des espèces exotiques basée sur leurs impacts environnementaux a récemment été proposée (Blackburn et al., 2014) ; les bipaliinés remplissent trois des critères énumérés dans le Tableau 1 de Blackburn et al. 2014 : compétition, prédation et empoisonnement/toxicité. Les deux premiers critères sont remplis par le caractère prédateur des bipaliinés, en particulier sur les proies plus grandes qu'eux (Ducey et al., 1999, Johri 1952, Zaborski 2002) ; la présence de tétrodotoxine (Stokes et al., 2014) répond au critère de toxicité, ce qui est renforcé par les rapports d'animaux qui vomissent les bipaliinés qu'ils ont ingérés (Winsor, 1983b). Cependant, en l'absence d'études écologiques, les bipaliinés devraient actuellement être classées comme « données déficientes » (encadré 1 de Blackburn et al., 2014).

En conclusion, nos résultats suggèrent fortement que les bipaliinés sont des espèces exotiques envahissantes en Europe et dans les territoires français d'outre-mer mentionnés dans ce document (**Figure 28**), mais une évaluation exacte de leur impact écologique nécessite des études écologiques qui sortent du cadre de cet article.

Comment des vers invasifs de 40 cm de long ont-ils pu échapper à l'attention des scientifiques pendant 20 ans?

Au début de notre étude, nous avons été intrigués par l'absence presque totale d'informations publiées sur la présence de bipaliinés en France. L'article de Vivant (2005) était le seul que nous ayons pu trouver, et comme il a été publié dans une revue mycologique

plutôt peu diffusée, il n'a certainement pas reçu une attention nationale ou internationale. De plus, nous sommes toujours surpris par l'absence totale de réponse des autorités scientifiques à la présence de ces vers. L'un des premiers signalements que nous avons reçus (2013) provenait d'un jardin d'enfants dans lequel les enfants auraient été effrayés par des centaines de « petits serpents » dans l'herbe (ceux-ci ont ensuite été identifiés comme *D. multilineatum*). Nous avons également reçu un rapport d'un citoyen qui a montré un long bipaliiné trouvé sur la fourrure de son chat à son vétérinaire, qui lui a dit qu'il s'agissait d'un ténia (cestode). D'autres citoyens ont expliqué qu'ils ont essayé d'obtenir des identifications de Plathelminthes terrestres auprès des universités locales et à qui on a dit que les vers étaient des sangsues et/ou des animaux communs et sans intérêt. Les Plathelminthes envahissants n'étaient pas connus en France il y a 10 ans (Justine et al., 2014a) et les professionnels impliqués dans ces anecdotes n'ont probablement jamais reçu d'enseignement à leur sujet. Il est clair qu'il faut plus d'éducation sur les Plathelminthes terrestres qui, en Europe, seront de plus en plus souvent rencontrés par les citoyens et les professionnels de l'agriculture, de l'aménagement paysager, des sciences vétérinaires et de la médecine.

Il est également étonnant que la présence de tels animaux remarquables n'ait jamais provoqué de réponse de la part des autorités scientifiques, bien que les signalements d'invasions d'insectes minuscules soient souvent suivis de mesures appropriées ; encore une fois, l'ignorance des scientifiques professionnels, des techniciens scientifiques et des naturalistes amateurs au sujet des Plathelminthes terrestres était probablement la raison. Il est significatif, à cet égard, que la première mention récente des Plathelminthes terrestres en France, par l'un d'entre nous (PG) ait été rendue publique dans un forum internet dédié aux insectes. Nous nous attendons à ce que les mesures prises au niveau européen augmentent l'information sur les Plathelminthes terrestres à l'avenir (Tsiamis et al., 2016).

Conclusion

Dans cet article, nous avons rapporté la présence de cinq espèces de Plathelminthes bipaliinés en France métropolitaine et dans quelques pays européens et territoires français d'outre-mer sur trois continents (**Figures 1, 27, 28**) : beaucoup reste à faire, y compris une description formelle de deux espèces. Bien sûr, les résultats rapportés ici ne représentent qu'une infime partie de la propagation de ces espèces envahissantes dans le monde. Des initiatives comme la nôtre, y compris les sciences participatives et des études moléculaires de

spécimens sélectionnés, devraient être entreprises dans le monde entier. Nous avons montré que le barcode moléculaire, basé sur le COI, était efficace pour l'identification des cinq espèces étudiées ici, fournissant ainsi des outils pour de futures études. Nous avons présenté des preuves que plusieurs espèces se propagent et qu'au moins l'une d'elles est un prédateur des vers de terre, qui sont des constituants importants de la faune du sol (Jones et al., 2001, Murchie et Gordon, 2013). Nous avons également démontré que les Plathelminthes bipaliinés correspondent bien à la définition des espèces exotiques envahissantes dans les documents européens scientifiques (Kettunen et al., 2009) et juridiques (Parlement européen 2014), mais nous reconnaissons qu'une évaluation précise de leur impact sur la biodiversité locale est nécessaire - mais ceci est en dehors de la portée de cet article. Récemment, une tendance à nier les risques posés par les espèces non indigènes a émergé (Ricciardi & Ryan 2018) ; en opposition à ce « négationnisme », nous croyons fermement que les vers invasifs, en tant que prédateurs actifs, constituent un danger pour la faune indigène partout où ils sont introduits.

Remerciements

Nous remercions tous les citoyens qui ont participé à l'enquête ; ceux qui ont envoyé des spécimens sont particulièrement remerciés. Les noms des citoyens bénévoles, et parfois des scientifiques, qui ont fourni des photographies et/ou des spécimens sont indiqués dans les **Tableaux 2, 5 à 8** et dans les **fichiers supplémentaires**. Nous présentons nos excuses aux citoyens qui ont aimablement fourni des informations mais dont nous n'indiquons pas le nom parce qu'ils n'ont pas pu être contactés plus tard pour obtenir un consentement formel. Le soutien des différentes Fédérations Régionales de Défense contre les Organismes Nuisibles (FREDON), en France métropolitaine et dans les départements d'outre-mer, est reconnu. LW remercie Martin Darley pour le spécimen de *Bipalium kewense* du Pakistan. Mme Vivant a fourni une référence rare.

Références

Álvarez-Presas M, Carbayo F, Rozas J, and Riutort M. 2011. Land planarians (Platyhelminthes) as a model organism for fine-scale phylogeographic studies : understanding patterns of biodiversity in the Brazilian Atlantic Forest hotspot. *Journal of Evolutionary Biology* 24 :887-896.

Álvarez-Presas M, Mateos E, Tudo A, Jones H, and Riutort M. 2014. Diversity of introduced terrestrial flatworms in the Iberian Peninsula : a cautionary tale. *PeerJ* 2 :e430.

Álvarez-Presas M, Mateos E, Vila-Farré M, Sluys R, and Riutort M. 2012. Evidence for the persistence of the land planarian species *Microplana terrestris* (Müller, 1774) (Platyhelminthes, Tricladida) in microrefugia during the Last Glacial Maximum in the northern section of the Iberian Peninsula. *Molecular Phylogenetics and Evolution* 64 :491-499.

Álvarez-Presas M, and Riutort M. 2014. Planarian (Platyhelminthes, Tricladida) diversity and molecular markers : a new view of an old group. *Diversity* 6 :323-338.

Ayadi ZEM, Gey D, Justine J-L, and Tazerouti F. 2017. A new species of *Microcotyle* (Monogenea : Microcotylidae) from *Scorpaena notata* (Teleostei : Scorpaenidae) in the Mediterranean Sea. *Parasitology International* 66 :37-42.

Blackburn TM, Essl F, Evans T, Hulme PE, Jeschke JM, Kühn I, Kumschick S, Marková Z, Mrugała A, Nentwig W, Pergl J, Pyšek P, Rabitsch W, Ricciardi A, Richardson DM, Sendek A, Vilà M, Wilson JRU, Winter M, Genovesi P, and Bacher S. 2014. A unified classification of alien species based on the magnitude of their environmental impacts. *PLoS Biology* 12 :e1001850.

Boag B, Palmer LF, Neilson R, and Chambers SJ. 1994. Distribution and prevalence of the predatory planarian *Artioposthia triangulata* (Dendy) (Tricladida : Terricola) in Scotland. *Annals of Applied Biology* 124 :165-171.

Bowles J, Blair D, and McManus DP. 1995. A molecular phylogeny of the human schistosomes. *Molecular Phylogenetics and Evolution* 4 :103-109.

Breugelmans K, Quintana Cardona J, Artois T, Jordaens K, and Backeljau T. 2012. First report of the exotic blue land planarian, *Caenoplana coerulea* (Platyhelminthes, Geoplanidae), on Menorca (Balearic Islands, Spain). *Zookeys* 199 :91-105.

Carbayo F, Alvarez-Presas M, Jones HD, and Riutort M. 2016. The true identity of *Obama* (Platyhelminthes : Geoplanidae) flatworm spreading across Europe. *Zoological Journal of the Linnean Society* 177 :5–28.

Chaabane A, Neifar L, Gey D, and Justine J-L. 2016. Species of *Pseudorhabdosynochus* (Monogenea, Diplectanidae) from groupers (*Mycteroperca* spp., Epinephelidae) in the Mediterranean and Eastern Atlantic Ocean, with special reference to the "beverleyburtonae group" and description of two new species. *PLoS ONE* 11 :e0159886.

Connella JV, and Stern DH. 1969. Land planarians : Sexuality and occurrence. *Transactions of the American Microscopical Society* 88 :309-311.

Convention sur la Diversité Biologique. 2018. What are Invasive Alien Species? https ://www.cbd.int/invasive/WhatareIAS.shtml consulted 14/02/2018.

de Beauchamp P. 1962. *Platydemus manokwari* n. sp., planaire terrestre de la Nouvelle-Guinée Hollandaise. *Bulletin de la Société Zoologique de France* 87 :609-615.

Dendy A. 1892. Short descriptions of new Land Planarians. *Proceedings of the Royal Society of Victoria* 4 : 35-38.

Dendy A. 1894. Additions to the cryptozoic fauna of New Zealand. *Annals and Magazine of Natural History* 14: 393-401.

Ducey PK, Messere M, Lapoint K, and Noce S. 1999. Lumbricid prey and potential herpetofaunal predators of the invading terrestrial flatworm *Bipalium adventitium* (Turbellaria : Tricladida : Terricola). *American Midland Naturalist* 141 :305-314.

Executive Order 13112. 1999. Executive Order 13112 of February 3, 1999, Invasive Species. Federal Register / Vol. 64, No. 25 / Monday, February 8, 1999 / Presidential Documents, p. 6183-6186, disponible à : https ://www.gpo.gov/fdsys/pkg/FR-1999-02-08/pdf/99-3184.pdf, consulté 14/02/2018.

Gerlach J. 2017. *Partula* survival in 2017, a survey of the Society islands. *Published by the author (29pp) – disponible à : https ://islandbiodiversitycom/ ; consulté 10 Novembre 2017.*

Gremigni V. 2003. Turbellaria. In : Stoch F, ed. *Checklist of the species of the Italian fauna On-line Version 20 http ://wwwfaunaitaliait/checklist/indexhtml*.

Hasegawa M, Kishino H, and Yano T-a. 1985. Dating of the human-ape splitting by a molecular clock of mitochondrial DNA. *Journal of Molecular Evolution* 22 :160-174.

Hebert PDN, Cywinska A, Ball SL, and deWaard JR. 2003. Biological identifications through DNA barcodes. *Proceedings of the Royal Society of London Series B : Biological Sciences* 270 :313-321.

Hebert PDN, and Gregory TR. 2005. The promise of DNA barcoding for taxonomy. *Systematic Biology* 54 :852-859.

Hyman LH. 1951. *The Invertebrates : Platyhelminthes and Rhynchocoela*. New York : MacGraw-Hill.

Infoclimat A. 2017. Normes et records 1961-1990. *Disponible à : https ://www.infoclimat.fr/, consulté 10 novembre2017.*

Johri LN. 1952. A report on a Turbellarian *Placocephalus kewense*, from Delhi State and its feeding behaviour on the live earthworm *Pheretima posthume*. *Science and Culture (Calcutta)* 18 :291.

Jones HD. 1999. A new genus and species of terrestrial planarian (Platyhelminthes ; Tricladida ; Terricola) from Scotland, and an emendation of the genus *Artioposthia*. *Journal of Natural History* 33 :387-394.

Jones HD, Santoro G, Boag B, and Neilson R. 2001. The diversity of earthworms in 200 Scottish fields and the possible effect of New Zealand land flatworms (*Arthurdendyus triangulatus*) on earthworm populations. *Annals of Applied Biology* 139 :75-92.

Jones HD, and Sluys R. 2016. A new terrestrial planarian species of the genus *Marionfyfea* (Platyhelminthes : Tricladida) found in Europe. *Journal of Natural History* 50 :2673-2690.

Jones HD, and Sterrer W. 2005. Terrestrial planarians (Platyhelminthes, with three new species) and nemertines of Bermuda. *Zootaxa* 1001 :31-58.

Justine J-L. 2017. Plathelminthes terrestres invasifs. Blog (en français). https ://sites.google.com/site/jljjustine/plathelminthe-terrestre-invasif.

Justine J-L, Thévenot J, and Winsor L. 2014a. Les sept plathelminthes invasifs introduits en France. *Phytoma* :28-32 doi :10.6084/m6089.figshare.1447202.

Justine J-L, Winsor L, Barrière P, Fanai C, Gey D, Han AWK, La Quay-Velazquez G, Lee BPY-H, Lefevre J-M, Meyer J-Y, Philippart D, Robinson DG, Thévenot J, and Tsatsia F. 2015. The invasive land planarian *Platydemus manokwari* (Platyhelminthes, Geoplanidae) : records from six new localities, including the first in the USA. *PeerJ* 3 :e1037.

Justine J-L, Winsor L, Gey D, Gros P, and Thévenot J. 2014b. The invasive New Guinea flatworm *Platydemus manokwari* in France, the first record for Europe : time for action is now. *PeerJ* 2 :e297. [Traduction française intégrale disponible – voir documents supplémentaires]

Kawakatsu M, Makino N, and Shirasawa Y. 1982. *Bipalium nobile* sp. nov. (Turbellaria, Tricladida, Terricola), a new land planarian from Tokyo. *Annotationes Zoologicae Japonense* 55 :236-262.

Kawakatsu M, Ogren RE, Froehlich EM, and Sasaki G-Y. 2002. Additions and corrections of the previous land planarian indices of the world (Turbellaria, Seriata, Tricladida, Terricola). *Bulletin of the Fuji Women's College (Series 2)* 40 :157-177.

Kawakatsu M, Sluys R, and Ogren RE. 2005. Seven new species of land planarian from Japan and China (Platyhelminthes, Tricladida, Bipaliidae), with a morphological review of all Japanese bipaliids and a biogeographic overview of Far Eastern species. *Belgian Journal of Zoology* 135 :53-77.

Kettunen M, Genovesi P, Gollasch S, Pagad S, Starfinger U, ten Brink P, and Shine C. 2009. *Technical support to EU strategy on invasive alien species (IAS) - Assessment of the impacts of IAS in Europe and the EU (final module report for the European*

Commission). Brussels, Belgium : Institute for European Environmental Policy (IEEP).

Košel V. 2002. Checklist of turbellaria in Slovakia. *Acta Zoologica Universitatis Comenianae* 44 :37-40.

Kubota S, and Kawakatsu M. 2010. Distribution record of a single species of the collective group *Diversibipalium* (Plathelminthes, Tricladida, Continenticola, Geoplanidae, Bipaliinae) in Wakayama Prefecture, Honshu, Japan, with a taxonomic note of new higher classification of the Tricladida. *Nanki Seibutsu* 52 :97-101.

Kumar S, Stecher G, and Tamura K. 2016. MEGA7 : Molecular Evolutionary Genetics Analysis version 7.0 for bigger datasets. *Molecular Biology and Evolution* 33 :1870-1874.

Lázaro EM, Sluys R, Pala M, Stocchino GA, Baguñà J, and Riutort M. 2009. Molecular barcoding and phylogeography of sexual and asexual freshwater planarians of the genus *Dugesia* in the Western Mediterranean (Platyhelminthes, Tricladida, Dugesiidae). *Molecular Phylogenetics and Evolution* 52 :835-845.

Littlewood DTJ, Rohde K, and Clough KA. 1997. Parasite speciation within or between host species? - Phylogenetic evidence from site-specific polystome monogeneans. *International Journal for Parasitology* 27 :1289-1297.

Makino N, and Shirasawa Y. 1983. Morphological and ecological comparison with two new species of elongated slender land planarians have several stripes and their new scientific names. *Bulletin of Tokyo Medical College* 9 :69-83 [In Japanese, English summary].

Mateos E, Tudó A, Álvarez-Presas M, and Riutort M. 2013. Planàries terrestres exòtiques a la Garrotxa. *Annals de la Delegació de la Garrotxa de la Institució Catalana d'Història Natural* 6 :67-73.

Mazza G, Menchetti M, Sluys R, Solà E, Riutort M, Tricarico E, Justine J-L, Cavigioli L, and Mori E. 2016. First report of the land planarian *Diversibipalium multilineatum* (Makino & Shirasawa, 1983) (Platyhelminthes, Tricladida, Continenticola) in Europe. *Zootaxa* 4067 :577–580.

Morffe J, García N, Adams BJ, and Hasegawa K. 2016. First record of the land planarian *Bipalium kewense* Moseley, 1878 (Tricladida : Geoplanidae : Bipaliinae) from Cuba. *BioInvasions Records* 5 :127-132.

Moseley H. 1877. Notes on the structure of several forms of land planarians, with a description of two new genera and several new species, and a list of all species at present known. *Quarterly Journal of Microscospical Science* 17 :273-292.

Moseley HN. 1878. Description of a new species of land-planarian from the hothouses at Kew Gardens. *Annals and Magazine of Natural History* 1 :237-239.

Murchie AK, and Gordon AW. 2013. The impact of the "New Zealand flatworm", *Arthurdendyus triangulatus*, on earthworm populations in the field. *Biological Invasions* 15 :569-586.

Parlement Européen. 2014. Regulation (EU) No 1143/2014 of the European Parliament and of the Council of 22 October 2014 on the prevention and management of the introduction and spread of invasive alien species. *Official Journal of the European Union* :L 317/335-L 317/355, disponible à : http ://data.europa.eu/eli/reg/2014/1143/oj dans de nombreuses langues.

Ricciardi A, and Ryan R. 2018. The exponential growth of invasive species denialism. *Biological Invasions* 3 :549–553

Saitou N, and Nei M. 1987. The neighbor-joining method : a new method for reconstructing phylogenetic trees. *Molecular Biology and Evolution* 4 :406-425.

Sluys R. 2016. Invasion of the Flatworms. *American Scientist* 104 :288-295.

Stokes AN, Ducey PK, Neuman-Lee L, Hanifin CT, French SS, Pfrender ME, Brodie ED, III, and Brodie Jr ED. 2014. Confirmation and distribution of Tetrodotoxin for the first time in terrestrial invertebrates : Two terrestrial flatworm species (*Bipalium adventitium* and *Bipalium kewense*). *PLoS ONE* 9 :e100718.

Tsiamis K, Gervasini E, D'Amico F, Deriu I, Katsanevakis S, Crocetta F, Zenetos A, Arianoutsou M, Backeljau T, Bariche M, Bazos I, Bertaccini A, Brundu G, Carrete M, Çinar ME, Curto G, Faasse M, Justine J-L, Király G, Langer MR, Levitt Ya, Panov

VE, Piraino S, Rabitsch W, Roques A, Scalera R, Shenkar N, Sîrbu I, Tricarico E, Vannini A, Vøllestad LA, Zikos A, and Cardoso AC. 2016. The EASIN Editorial Board : quality assurance, exchange and sharing of alien species information in Europe. *Management of Biological Invasions* 7 :321–328.

Union Internationale pour la Conservation de la Nature. 2018. Invasive species, disponible à : https ://www.iucn.org/theme/species/our-work/invasive-species, consulté 14/02/2018.

Vanhove MP, Tessens B, Schoelinck C, Jondelius U, Littlewood DT, Artois T, and Huyse T. 2013. Problematic barcoding in flatworms : A case-study on monogeneans and rhabdocoels (Platyhelminthes). *Zookeys* :355-379.

Vivant J. 2005. *Bipalium kewense* Moseley, ver tropical terricole, existe à Orthez (Pyr. atl.). *Bulletin de la Société Mycologique Landaise* :46-48.

von Graff L. 1899. *Monographie der Turbellarien. II. Tricladida, Terricola (Landplanarien)*. Leipzig : Englemann.

Ward RD, Zemlak TS, Innes BH, Last PR, and Hebert PD. 2005. DNA barcoding Australia's fish species. *Philosophical Transactions of the Royal Society of London B Biological Sciences* 360 :1847-1857.

Winsor L. 1983a. A revision of the Cosmopolitan land planarian *Bipalium kewense* Moseley, 1878 (Turbellaria : Tricladida : Terricola). *Zoological Journal of the Linnean Society* 79 :61-100.

Winsor L. 1983b. Vomiting of land planarians (Turbellaria : Tricladida : Terricola) ingested by cats. *Australian Veterinary Journal* 60 :282-283.

Winsor L. 1991. A provisional classification of Australian terrestrial geoplanid flatworms (Tricladida : Terricola : Geoplanidae). *Victorian Naturalist (Blackburn)* 108 :42-49.

Winsor L, Johns PM, and Barker GM. 2004. Terrestrial planarians (Platyhelminthes : Tricladida : Terricola) predaceous on terrestrial gastropods. In : Barker GM, ed. *Natural enemies of terrestrial molluscs*. Oxfordshire, UK : CAB International, 227-278.

Winsor L, Johns PM, and Yeates GW. 1998. Introduction, and ecological and systematic background, to the Terricola (Tricladida). *Pedobiologia* 42 389-404.

Wizen G. 2015. Photograph. Caption : Huge terrestrial flatworm (*Bipalium kewense*), Mindo, Ecuador, March. Nature Picture Library, Image number 01504312. Available from : https ://www.naturepl.com/search/preview/huge-terrestrial-flatworm-bipalium-kewense-mindo-ecuador-march/0_01504312.html. Consulted on 06 Nov 2017.

Zaborski ER. 2002. Observations on feeding behavior by the terrestrial flatworm *Bipalium adventitium* (Platyhelminthes : Tricladida : Terricola) from Illinois. *American Midland Naturalist* 148 :401-408.

Tableaux

Tableau 1. Plathelminthes invasifs trouvés en Europe, auteurs de taxons et références clés.

Ce tableau fournit des informations complètes sur les auteurs et les taxons et les combinaisons, ce qui rend le texte général plus léger. Sluys (2016) a énuméré d'autres espèces avec des données et des informations limitées: *Artioposthia exulans* Dendy, 1901, *Australoplana sanguinea* (Moseley, 1877), *Dolichoplana striata* Moseley, 1877, *Kontikia andersoni* Jones, 1981.

Taxon et auteurs	Synonymes	Références pour le taxon	Référence principale pour la présence en Europe
Arthurdendyus triangulatus (Dendy, 1894) Jones, 1999	*Artioposthia triangulata*	Dendy 1894, Jones 1999	Boag et al. 1994
Platydemus manokwari De Beauchamp, 1963		de Beauchamp 1962	Justine et al. 2014b
Obama nungara Carbayo, Álvarez-Presas, Jones & Riutort, 2016	*Obama marmorata*	Carbayo et al. 2016	Carbayo et al. 2016
Parakontikia ventrolineata (Dendy, 1892) Winsor, 1991	*Kontikia ventrolineata*	Dendy 1892, Winsor 1991	Álvarez-Presas et al. 2014
Caenoplana coerulea Moseley, 1877		Moseley 1877	Álvarez-Presas et al. 2014, Breugelmans et al. 2012
Caenoplana bicolor (Graff, 1899) Winsor, 1991	Geoplana bicolor	von Graff 1899, Winsor 1991	Álvarez-Presas et al. 2014
Marionfyfea adventor Jones & Sluys, 2016		Jones & Sluys 2016	Jones & Sluys 2016
Diversibipalium multilineatum (Makino & Shirasawa, 1983) Kubota & Kawakatsu, 2010	*Bipalium multilineatum*	Makino & Shirasawa 1983, Kubota & Kawakatsu 2010	Mazza et al. 2016, cet article
Bipalium kewense Moseley, 1878		Moseley 1878	Cet article

Tableau 2: Spécimens avec identification moléculaire.

Espèce	MNHN	N° GenBank	Date	Localité	Département / État	Pays - Continent	COI	Réplicats	Collecteur
BK	JL089	MG655587	12/11/2013	Saint Pée sur Nivelle	Pyrénées-Atlantiques	France Métro. - Europe	court	1	Anonyme
BK	JL160	MG655605	23/05/2014	Cannes	Alpes-Maritimes	France Métro. - Europe	court	1	Iachia, Valeria
BK	JL167	MG655615	24/08/2014	Orthez	Pyrénées-Atlantiques	France Métro. - Europe	court	1	Rougeux, Christian
BK	JL174	MG655616	03/09/2014	Bassussary	Pyrénées-Atlantiques	France Métro. - Europe	long	1	Mercader, Elisabeth
BK	JL176 **	MG655617	05/09/2014	Auxerre (serre)	Yonne	France Métro. - Europe	long	1	Bellina, Arnaud
BK	JL184	MG655603	Oct. 2014	Ustaritz	Pyrénées-Atlantiques	France Métro. - Europe	court	1	Goyheneche, Iker
BK	JL188	MG655604	08/10/2014	Miramar	Grande Porto	Portugal - Europe	court	1	Soarès, Luciana
BK	JL212	MG655592	19/12/2014	Mimbastes	Landes	France Métro. - Europe	long	1	Jouveau, Séverin
BK	JL224	MG655607	23/02/2015	Trois Rivières	Guadeloupe	Guadeloupe – Amérique C.	long	1	Van Laere, Guy
BK	JL233	MG655608	27/09/2014	Monaco	Monaco	Monaco - Europe	long	3	Dusoulier, François
BK	JL253	MG655609	21/03/2015	Trois Rivières	Guadeloupe	Guadeloupe – Amérique C.	court	1	Van Laere, Guy
BK	JL254	MG655610	15/05/2015	Matoury	Guyane Française	Guyane Fr. – Amérique S.	court	2	Girault, Rémi
BK	JL270	MG655594	23/04/2015	Ducos	Martinique	Martinique -– Amérique C.	long	1	Lucas, Pierre-Damien
BK	JL308	MG655602	08/09/2016	Morne Vert	Guadeloupe	Guadeloupe -– Amérique C.	court	1	Coulis, Mathieu
BV	JL073	MG655611	Août 2013	Sanibel	Floride	USA - Amérique Nord	court	1	Justine, Jean-Lou
BV	JL163	MG655613	July 2014	Sanibel	Floride	USA - Amérique Nord	court	1	Justine, Jean-Lou
BV	JL164	MG655614	July 2014	Sanibel	Floride	USA - Amérique Nord	court	1	Justine, Jean-Lou
BV	JL213	MG655593	29/11/2014	Anse-Bertrand	Guadeloupe	Guadeloupe – Amérique C.	long	1	Charles, Laurent
BV	JL268	MG655595	Déc. 2014	Montserrat	Montserrat	Montserrat -– Amérique C.	court	1	Shoobs, Nathaniel F.
BV	JL307	MG655601	19/11/2015	Morne Vert	Guadeloupe	Guadeloupe – Amérique C.	court	1	Coulis, Mathieu
DM	JL177 *	KT922162	30/09/2014	Léguevin	Haute-Garonne	France Métro. - Europe	long	1	Chaim, Florence
DM	JL059	MG655618	15/06/2013	La Bastide de Serou	Ariège	France Métro. - Europe	court	1	Brugnara, Sébastien
DM	JL142	MG655612	22/04/2014	Saubrigues	Landes	France Métro. - Europe	long	2	Robineau, Thiérry
DM	JL161	MG655606	11/06/2015	Bellocq	Pyrénées-Atlantiques	France Métro. - Europe	long	1	Audiot, Marie-Claude
DM	JL208	MG655589	11/06/2014	Bellocq	Pyrénées-Atlantiques	France Métro. - Europe	long	1	Audiot, Marie-Claude
DM	JL209	MG655590	12/06/2014	Bellocq	Pyrénées-Atlantiques	France Métro. - Europe	long	1	Audiot, Marie-Claude
DM	JL210	MG655591	Juin 2014	Bellocq	Pyrénées-Atlantiques	France Métro. - Europe	long	1	Audiot, Marie-Claude
DM	JL298 ***	MG655600	01/06/2016	Novazzano	Canton du Tessin	Suisse - Europe	long	1	Pollini, Lucia
DBleu	JL280	MG655596	2015	Mtsamboro	Mayotte	Mayotte - Afrique	long	1	Charles, Laurent
DBleu	JL281	MG655597	29/04/2015	Mtsamboro	Mayotte	Mayotte - Afrique	long	3	Charles, Laurent
DBleu	JL282	MG655598	30/04/2015	Ouangani	Mayotte	Mayotte - Afrique	long	1	Charles, Laurent
DBleu	JL284	MG655599	05/05/2015	Mtsamboro	Mayotte	Mayotte - Afrique	long	1	Charles, Laurent
DNoir	JL090	MG655588	12/11/2013	Saint Pée sur Nivelle	Pyrénées-Atlantiques	France Métro. - Europe	court	1	Anonyme

* JL177 déjà publié (Mazza et al., 2016); ** spécimen de serre, tous les autres ont été récoltés en extérieur; *** Spécimen MCSN 719.990 / 77.590 conservé au Museo Cantonale de Storia Naturale, Lugano, Suisse, transmis par Jean Mariaux (Genève, Suisse). BK: *Bipalium kewense*; BV: *Bipalium vagum*; DM: *Diversibipalium multilineatum*; Dbleu: *Diversibipalium* sp. « bleu »; Dnoir: *Diversibipalium* sp. « noir ».

La plupart des collecteurs étaient des particuliers; mais ces collecteurs sont des professionnels: Arnaud Bellina, FREDON Bourgogne; Laurent Charles, Muséum Science et Nature, Bordeaux; Mathieu Coulis, CIRAD Martinique; Pierre-Damien Lucas, FREDON Martinique; Guy Van Laere, Parc National de Guadeloupe.

Tableau 3. Divergences entre les séquences « courtes ».

Il y avait un total de 266 positions dans les données finales.

	kewense	multilineatum	nobile	« noir »	« bleu »	vagum
multilineatum	0.109					
nobile	0.131	0.131				
« noir »	0.149	0.164	0.163			
« bleu »	0.206	0.202	0.164	0.192		
vagum	0.140	0.168	0.163	0.140	0.159	
adventium	0.136	0.178	0.173	0.173	0.212	0.164

Tableau 4. Divergences entre les séquences « longues ».

Il y avait un total de 857 positions dans les données finales.

	kewense	multilineatum	« bleu »
multilineatum	0.159		
« bleu »	0.230	0.259	
vagum	0.167	0.179	0.223

Tableau 5. Mesures de spécimens vivants de bipaliinés, estimées à partir de photographies avec des échelles obtenues grâce aux sciences participatives.

Espèce	N° de spécimen MNHN ou photographie de science participative	Localité	Longueur du corps (cm)
Bipalium kewense	MNHN JL089	France	21
	MNHN JL184	France	16
	MNHN JL188	Portugal	25
	MNHN JL224	Guadeloupe	21
	MNHN JL270	Martinique	11
	K04	Guadeloupe	13
	K05	Martinique	20
	K07	La Réunion	10
	K24	France	20
	K25	France	27
	K28	France	15
	K35	France	17
Diversibipalium multilineatum	MNHN JL177	France	15
	MNHN JL059	France	21
Bipalium vagum	V04	Guyane Française	3,6

Tableau 6. Signalements de *Bipalium kewense* identifiés à partir de photographies.

Les photographies ont été obtenues grâce aux sciences participatives; les spécimens ont été identifiés à partir de photographies par les auteurs. Aucune identification moléculaire n'était possible. Il y avait 36 signalements (35 en extérieur et un en serre). Le nom des auteurs des photographies n'est indiqué que lorsqu'un consentement formel à la publication a été obtenu des auteurs. Les photographies sont dans le Supplément 2. Pour le premier signalement, voir aussi Gerlach (2017).

#	Date	Localité	Département / État	Pays - Continent	Origine des données
**K01**	20/08/2017	Bora Bora	Polynésie Française	Polynésie Française - Océanie	Gerlach, Justin
**K02**	13/10/2010	Basse-Terre	Guadeloupe	Guadeloupe – Amérique C.	Guezennec, Pierre et Claudine
**K03**	22/01/2014	Non précisée	Guadeloupe	Guadeloupe – Amérique C.	Consentement non reçu
**K04**	14/01/2007	Petit-Bourg	Guadeloupe	Guadeloupe – Amérique C.	Lurel, Félix
**K05**	19/02/2015	La Trinité	Martinique	Guadeloupe – Amérique C.	Delannoye, Régis
**K06**	19/04/2016	Saint Joseph	Martinique	Guadeloupe – Amérique C.	Andrebe, Silvio
**K07**	25/08/2017	Plaine des Cafres	La Réunion	La Réunion - Afrique	Pronier, Pascal
**K08**	03/11/2013	Cagnes-sur-Mer	Alpes-Maritimes	France Métro. - Europe	Gros, Pierre
**K09**	19/01/2014	Cagnes-sur-Mer	Alpes-Maritimes	France Métro. - Europe	Gros, Pierre
**K10**	05/11/2014	Cagnes-sur-Mer	Alpes-Maritimes	France Métro. - Europe	Gros, Pierre
**K11**	16/10/2013	Beaulieu-sur-Mer	Alpes-Maritimes	France Métro. - Europe	Pelcer, Jean-Paul
**K12**	21/07/2014	Nice	Alpes-Maritimes	France Métro. - Europe	Gerriet, Olivier *
**K13**	15/10/2014	Appietto	Corse-Sud (Corse)	France Métro. - Europe	Consentement non reçu
**K14**	17/10/2013	Pietrosella	Corse-Sud (Corse)	France Métro. - Europe	Senee, Patrick
**K15**	23/08/2014	Arcachon	Gironde	France Métro. - Europe	Consentement non reçu
**K16**	21/11/2002	Saint-Jean-de-Vedas	Hérault	France Métro. - Europe	Peaucellier, Gérard
**K17**	27/10/2014	Biscarosse	Landes	France Métro. - Europe	Consentement non reçu
**K18**	27/09/2008	Hagetmau	Landes	France Métro. - Europe	Jeannotin, Josette

**K19**	22/09/2016	Nantes	Loire-Atlantique	France Métro. - Europe	Consentement non reçu
**K20**	16/10/2014	Grimaud	Var	France Métro. - Europe	Bernez, Alain
**K21**	01/08/2014	Toulon	Var	France Métro. - Europe	Consentement non reçu
**K22**	29/07/2014	Sens (serre)	Yonne	France Métro. - Europe	Burel, Jonathan **
**K23**	23/10/2017	Peyrouse	Hautes-Pyrénées	France Métro. - Europe	Tremosa, Clémence
**K24**	17/12/2014	Arthez de Béarn	Pyrénées-Atlantiques	France Métro. - Europe	Sillard, Dominique
**K25**	17/09/2017	Billère	Pyrénées-Atlantiques	France Métro. - Europe	Rolland, Geneviève
**K26**	28/01/2018	Billère	Pyrénées-Atlantiques	France Métro. - Europe	Rolland, Geneviève
**K27**	20/09/2014	Bayonne	Pyrénées-Atlantiques	France Métro. - Europe	Bonnefous, François
**K28**	18/08/2014	Hasparren	Pyrénées-Atlantiques	France Métro. - Europe	Voise, Mireille
**K29**	22/04/2016	Jurançon (près de)	Pyrénées-Atlantiques	France Métro. - Europe	Pauchet, Marjolaine
**K30**	29/04/2016	Nay	Pyrénées-Atlantiques	France Métro. - Europe	Lamaille, Corinne
**K31**	28/09/2014	Orthez	Pyrénées-Atlantiques	France Métro. - Europe	Rougeux, Christian
**K32**	22/08/2016	Saint Jean de Luz	Pyrénées-Atlantiques	France Métro. - Europe	Centelles, Ruben
**K33**	01/01/1999	Urcuit	Pyrénées-Atlantiques	France Métro. - Europe	Esposito, Mario
**K34**	14/09/2014	Urt	Pyrénées-Atlantiques	France Métro. - Europe	Chanderot, Vincent
**K35**	12/08/2017	Ustaritz	Pyrénées-Atlantiques	France Métro. - Europe	Lescourret, Monique & Bernard
**K36**	14/09/2014	Villefranque	Pyrénées-Atlantiques	France Métro. - Europe	Consentement non reçu

* Muséum d'Histoire Naturelle, Nice, France; ** FREDON Île de France.

Tableau 7. Signalements de *Diversibipalium multilineatum* identifiés à partir de photographies.

Les photographies ont été obtenues grâce aux sciences participatives; les spécimens ont été identifiés à partir de photographies par les auteurs. Aucune identification moléculaire n'était possible. Il y a 11 signalements, dont 2 provenant de serres. Le nom des auteurs des photographies n'est indiqué que lorsqu'un consentement formel à la publication a été obtenu des auteurs. Les photographies sont dans le supplément 2.

#	Date	Localité	Département / État	Pays - Continent	Origine
**M01**	27/06/2010	Longages	Haute-Garonne	France Métro. - Europe	Lombard, Yoann
**M02**	22/03/2011	Longages	Haute-Garonne	France Métro. - Europe	Lombard, Yoann
**M03**	06/07/2016	Saint-Egrève	Isère	France Métro. - Europe	Tuaillon, Jean-Louis
**M04**	17/05/2017	Saint-Egrève	Isère	France Métro. - Europe	Tuaillon, Jean-Louis
**M05**	27/06/2016	Benquet	Landes	France Métro. - Europe	Broustaut, François
**M06**	28/03/2014	Cahors (serre)	Lot	France Métro. - Europe	Consentement non reçu
**M07**	04/07/2014	Andilly (serre)	Val d'Oise	France Métro. - Europe	Burel, Jonathan *
**M08**	27/04/2015	Magny-en-Vexin	Val d'Oise	France Métro. - Europe	Mellac, Céline
**M09**	29/05/2016	Magny-en-Vexin	Val d'Oise	France Métro. - Europe	Mellac, Céline
**M10**	19/04/2010	Sames	Pyrénées-Atlantiques	France Métro. - Europe	Grenier-Falaise, Nadine
**M11**	07/04/2017	Billère	Pyrénées-Atlantiques	France Métro. - Europe	Vincent, Jean-François

* FREDON Île de France.

Tableau 8. Signalements de *Bipalium vagum* identifiés à partir de photographies.

Les photographies ont été obtenues grâce aux sciences participatives; les spécimens ont été identifiés à partir de photographies par les auteurs. Aucune identification moléculaire n'était possible. Il y a 33 enregistrements, tous en extérieur. Le nom des auteurs des photographies n'est indiqué que lorsqu'un consentement formel à la publication a été obtenu des auteurs. Les photographies sont dans le supplément 2.

#	Date	Localité	Département / État	Pays - Continent	Origine
**V01**	21/06/2005	Cayenne	Guyane Française	Guyane Française – Amérique S.	Girault, Rémi
**V02**	15/05/2017	Macouria	Guyane Française	Guyane Française – Amérique S.	Boutin, Élodie
**V03**	12/05/2017	Saint-Laurent-du-Maroni	Guyane Française	Guyane Française – Amérique S.	Muraine, François Xavier
**V04**	26/07/2017	Saül	Guyane Française	Guyane Française – Amérique S.	Sant, Sébastien
**V05**	21/08/2017	Petit-Bourg	Guadeloupe	Guyane Française – Amérique S.	De Tienda, Marine
**V06**	24/11/2013	Gosier	Guadeloupe	Guadeloupe – Amérique C.	Consentement non reçu
**V07**	30/10/2016	Gosier	Guadeloupe	Guadeloupe – Amérique C.	Brisson, Bernard
**V08**	22/11/2013	Petit Bourg	Guadeloupe	Guadeloupe – Amérique C.	Oettly, Olivier
**V09**	22/11/2014	Petit Bourg	Guadeloupe	Guadeloupe – Amérique C.	Marques, Maryvonne
**V10**	29/04/2011	Petit-Bourg	Guadeloupe	Guadeloupe – Amérique C.	Guezennec, Pierre et Claudine
**V11**	21/10/2017	Petit-Canal	Guadeloupe	Guadeloupe – Amérique C.	Charles, Laurent
**V12**	29/11/2016	Le Moule	Guadeloupe	Guadeloupe – Amérique C.	Consentement non reçu
**V13**	25/07/2010	La Trinité	Martinique	Martinique – Amérique C.	Delannoye, Régis
**V14**	18/11/2015	Morne Vert	Martinique	Martinique – Amérique C.	Coulis, Mathieu
**V15**	05/01/2018	Trois Ilets	Martinique	Martinique – Amérique C.	Consentement non reçu
**V16**	01/04/2014	Saint Barthélemy	Saint Barthélemy	Saint Barthélemy – Amérique C.	Moulard, Grégory
**V17**	01/05/2014	Saint Barthélemy	Saint Barthélemy	Saint Barthélemy – Amérique C.	Consentement non reçu
**V18**	11/05/2014	Saint Martin	Saint Martin	Saint Martin – Amérique C.	Yokoyama, Mark
**V19**	21/11/2015	Avirons	La Réunion	La Réunion - Afrique	Consentement non reçu

**V20**	23/03/2017	Bras Panon	La Réunion	La Réunion - Afrique	Saman-Latchimy, Teddy
**V21**	29/03/2017	Le Tampon	La Réunion	La Réunion - Afrique	Consentement non reçu
**V22**	26/10/2014	Petite Ile	La Réunion	La Réunion - Afrique	Abonnenc, José
**V23**	12/03/2016	Petite Ile	La Réunion	La Réunion - Afrique	Le Gars, René
**V24**	16/05/2014	Saint Louis	La Réunion	La Réunion - Afrique	Faujour, Anne
**V25**	08/04/2014	Saint Paul	La Réunion	La Réunion - Afrique	Consentement non reçu
**V26**	16/03/2016	Saint Pierre	La Réunion	La Réunion - Afrique	Collet, Jean
**V27**	10/03/2013	Sainte Marie	La Réunion	La Réunion - Afrique	Fontaine, Romuald
**V28**	06/03/2016	Sainte Marie	La Réunion	La Réunion - Afrique	Fontaine, Romuald
**V29**	12/02/2009	Non précisée	La Réunion	La Réunion - Afrique	Gilson, Michel
**V30**	03/03/2010	Non précisée	La Réunion	La Réunion - Afrique	Gilson, Michel
**V31**	01/05/2011	Non précisée	La Réunion	La Réunion - Afrique	Martiré, Dominique
**V32**	28/10/2013	Non précisée	La Réunion	La Réunion - Afrique	Martiré, Dominique
**V33**	17/08/2015	Non précisée	La Réunion	La Réunion - Afrique	Lacoste, Marie

Tableau 9. Signalements de *Diversibipalium* « bleu » identifiés à partir de photographies

. 1 signalement.

**Date**	**Localité**	**Département / État**	**Pays - Continent**	**Origine**
07/03/2014	Non précisée	Mayotte	Mayotte - Afrique	Duperron, Benoît

Figures

Figure 1. Carte de la France métropolitaine (incluant la Corse) montrant des signalements de Plathelminthes bipaliinés.

La plupart des signalements rapportés dans ce document sont en extérieur mais deux proviennent de serres. Notez la concentration des signalements dans la région Sud-Est, dans le département des Pyrénées-Atlantiques.

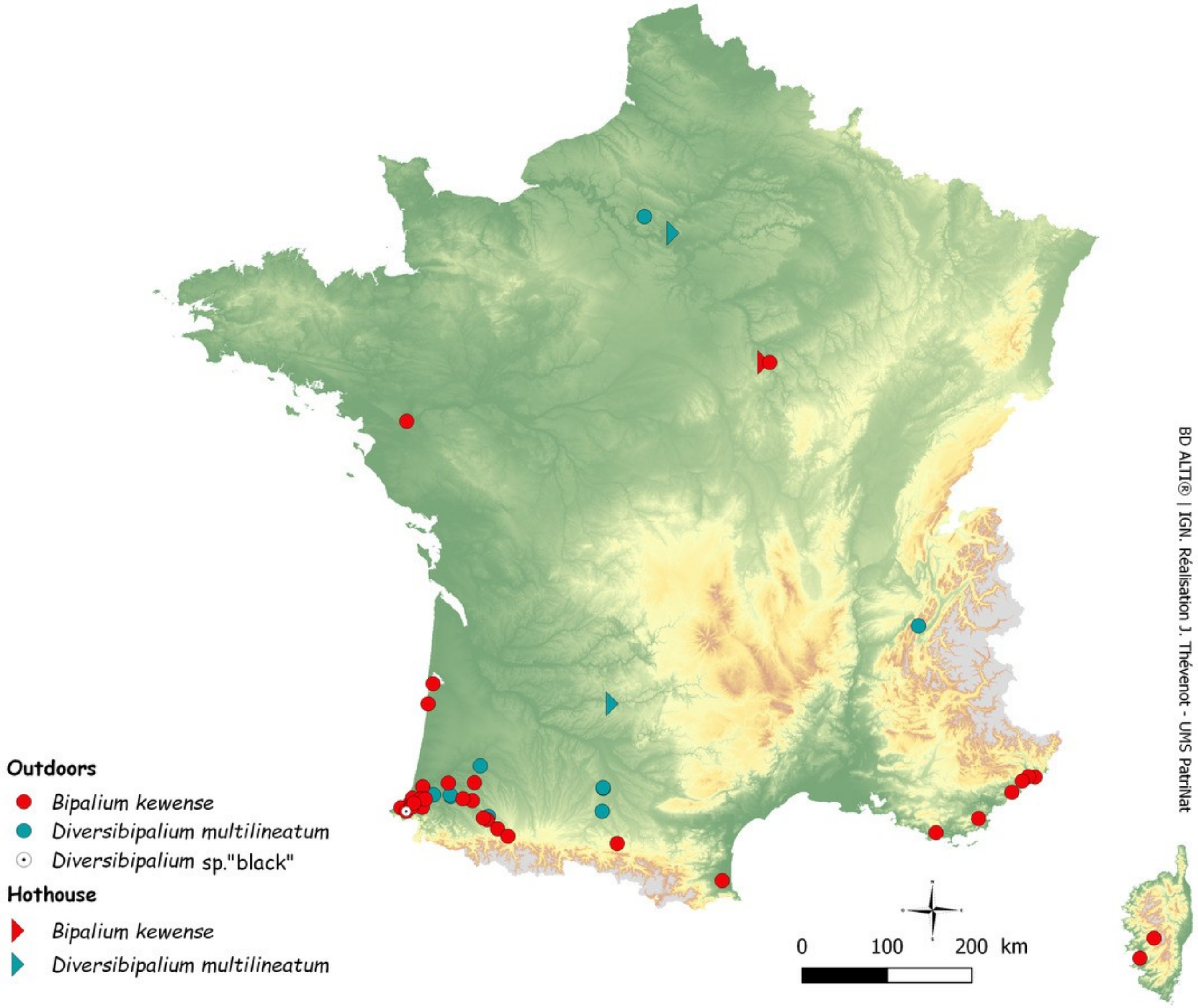

Figure 2 Relations évolutives des taxons

(page suivante)

L'arbre montré ici a été déduit en utilisant la méthode Neighbor-Joining. Le pourcentage d'arbres répliqués dans lesquels les taxons associés sont groupés dans le test de bootstrap (1000 réplicats) est affiché à côté des branches, uniquement lorsque > 70. L'histoire de l'évolution inférée par la méthode du Maximum de Vraisemblance avait une topologie similaire. Dans les deux arbres, les branches représentant les quatre espèces avec plusieurs échantillons (*Bipalium kewense*, *Bipalium vagum*, *Diversibipalium multilineatum* et *Diversibipalium* « bleu ») avaient toutes des valeurs bootstrap de 100%, mais les valeurs bootstrap pour les nœuds supérieurs étaient très faibles. Nous considérons que l'arbre est informatif pour montrer l'identité génétique de tous les spécimens d'une espèce, mais pas pour déduire les relations entre les taxons. Les nouveaux signalements avec des informations moléculaires sont indiqués par *. Pour les signalements en France métropolitaine, le numéro indique le code de département (c'est-à-dire par exemple 64: Pyrénées-Atlantiques).

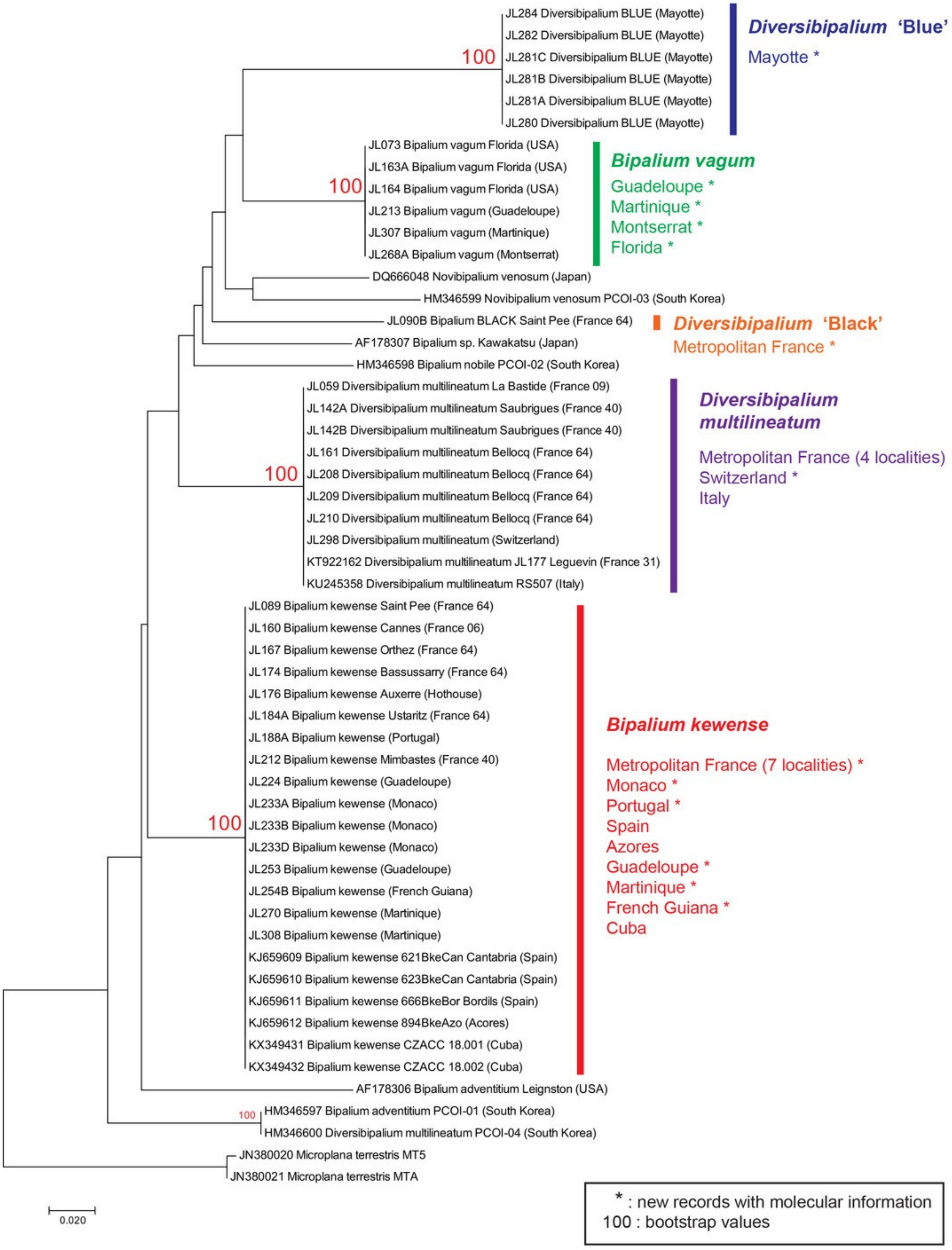
JL284 Diversibipalium BLUE (Mayotte)
JL282 Diversibipalium BLUE (Mayotte)
100
JL281C Diversibipalium BLUE (Mayotte)
JL281B Diversibipalium BLUE (Mayotte)
JL281A Diversibipalium BLUE (Mayotte)
JL280 Diversibipalium BLUE (Mayotte)
Diversibipalium 'Blue'
Mayotte *
JL073 Bipalium vagum Florida (USA)
JL163A Bipalium vagum Florida (USA)
100
JL164 Bipalium vagum Florida (USA)
JL213 Bipalium vagum (Guadeloupe)
JL307 Bipalium vagum (Martinique)
JL268A Bipalium vagum (Montserrat)
Bipalium vagum
Guadeloupe *
Martinique *
Montserrat *
Florida *
DQ666048 Novibipalium venosum (Japan)
HM346599 Novibipalium venosum PCOI-03 (South Korea)
JL090B Bipalium BLACK Saint Pee (France 64)
Diversibipalium 'Black'
Metropolitan France *
AF178307 Bipalium sp. Kawakatsu (Japan)
HM346598 Bipalium nobile PCOI-02 (South Korea)
JL059 Diversibipalium multilineatum La Bastide (France 09)
JL142A Diversibipalium multilineatum Saubrigues (France 40)
JL142B Diversibipalium multilineatum Saubrigues (France 40)
JL161 Diversibipalium multilineatum Bellocq (France 64)
100
JL208 Diversibipalium multilineatum Bellocq (France 64)
JL209 Diversibipalium multilineatum Bellocq (France 64)
JL210 Diversibipalium multilineatum Bellocq (France 64)
JL298 Diversibipalium multilineatum (Switzerland)
KT922162 Diversibipalium multilineatum JL177 Leguevin (France 31)
KU245358 Diversibipalium multilineatum RS507 (Italy)
Diversibipalium multilineatum
Metropolitan France (4 localities)
Switzerland *
Italy
JL089 Bipalium kewense Saint Pee (France 64)
JL160 Bipalium kewense Cannes (France 06)
JL167 Bipalium kewense Orthez (France 64)
JL174 Bipalium kewense Bassussarry (France 64)
JL176 Bipalium kewense Auxerre (Hothouse)
JL184A Bipalium kewense Ustaritz (France 64)
JL188A Bipalium kewense (Portugal)
JL212 Bipalium kewense Mimbastes (France 40)
JL224 Bipalium kewense (Guadeloupe)
JL233A Bipalium kewense (Monaco)
100
JL233B Bipalium kewense (Monaco)
JL233D Bipalium kewense (Monaco)
JL253 Bipalium kewense (Guadeloupe)
JL254B Bipalium kewense (French Guiana)
JL270 Bipalium kewense (Martinique)
JL308 Bipalium kewense (Martinique)
KJ659609 Bipalium kewense 621BkeCan Cantabria (Spain)
KJ659610 Bipalium kewense 623BkeCan Cantabria (Spain)
KJ659611 Bipalium kewense 666BkeBor Bordils (Spain)
KJ659612 Bipalium kewense 894BkeAzo (Acores)
KX349431 Bipalium kewense CZACC 18.001 (Cuba)
KX349432 Bipalium kewense CZACC 18.002 (Cuba)
Bipalium kewense
Metropolitan France (7 localities) *
Monaco *
Portugal *
Spain
Azores
Guadeloupe *
Martinique *
French Guiana *
Cuba
AF178306 Bipalium adventitium Leignston (USA)
100
HM346597 Bipalium adventitium PCOI-01 (South Korea)
HM346600 Diversibipalium multilineatum PCOI-04 (South Korea)
JN380020 Microplana terrestris MT5
JN380021 Microplana terrestris MTA
0.020
* : new records with molecular information
100 : bootstrap values

Figure 3. *Bipalium kewense*, morphologie générale.

Aspect dorsal du Plathelminthe avec une vue partielle de la surface ventrale. Notez l'extrémité postérieure arrondie indiquant la reproduction par scissiparité. Photo par Pierre Gros.

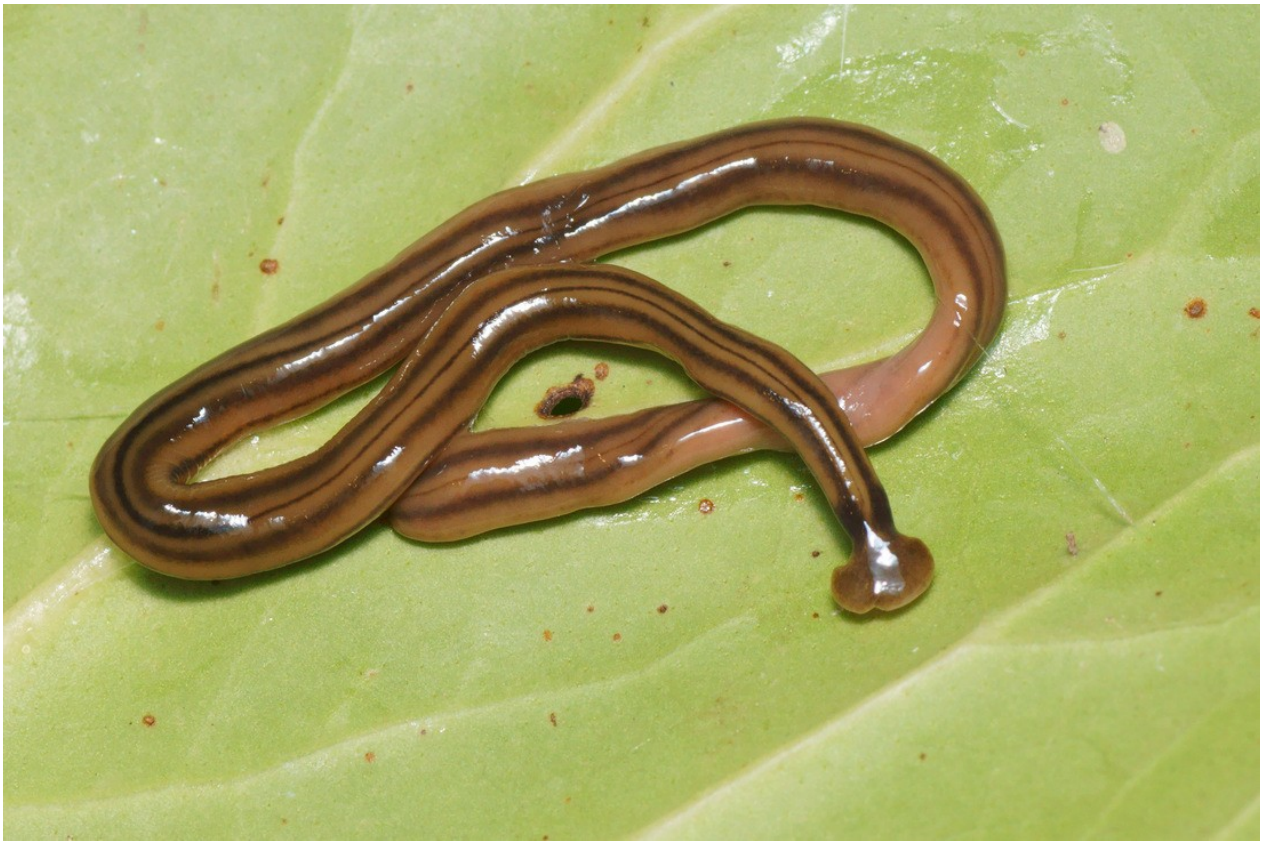

Figure 4. *Bipalium kewense*, morphologie générale de l'extrémité antérieure dorsale.

La plaque frontale déployée, la bande transversale noire (« collier ») au niveau du cou, et les bandes longitudinales médianes jumelées latérales et marginales dorsolatérales sont évidentes. Notez que la bande médiane dorsale ne passe pas sur la plaque frontale. Photo par Pierre Gros.

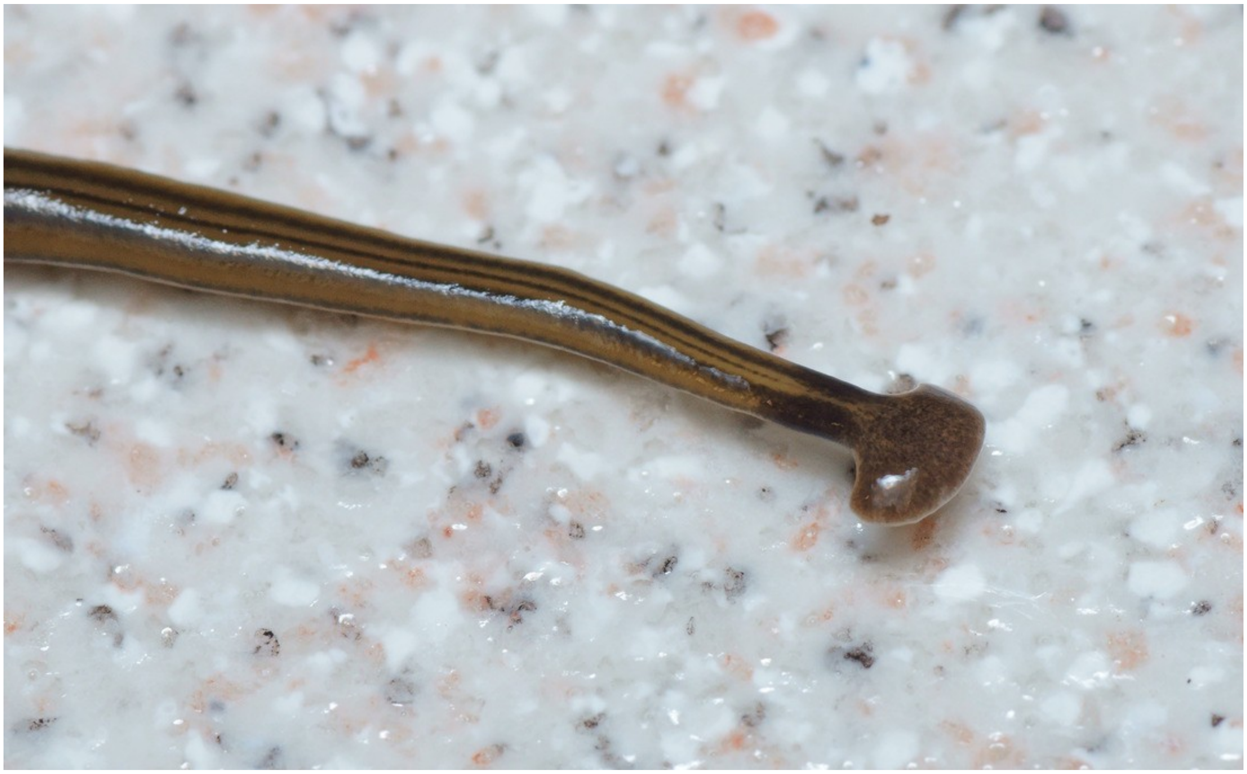

Figure 5. *Bipalium kewense*, vue de côté de la plaque frontale.

Bipalium kewense chasse ses proies, des vers de terre, en utilisant des mécanorécepteurs et des chémorécepteurs situés le long de la marge antérieur de la plaque frontale. Ces récepteurs sont exposés lorsque les papilles autour de la plaque frontale sont distendues et déplacées comme des doigts trapus dans un mouvement ondulant pour détecter l'environnement, ce qui est visible dans cette image. La surface inférieure de la plaque frontale est richement dotée d'une variété de glandes qui comprennent des sécrétions avec des fonctions adhésives, lubrifiantes et probablement liées aux toxines. Photo par Pierre Gros.

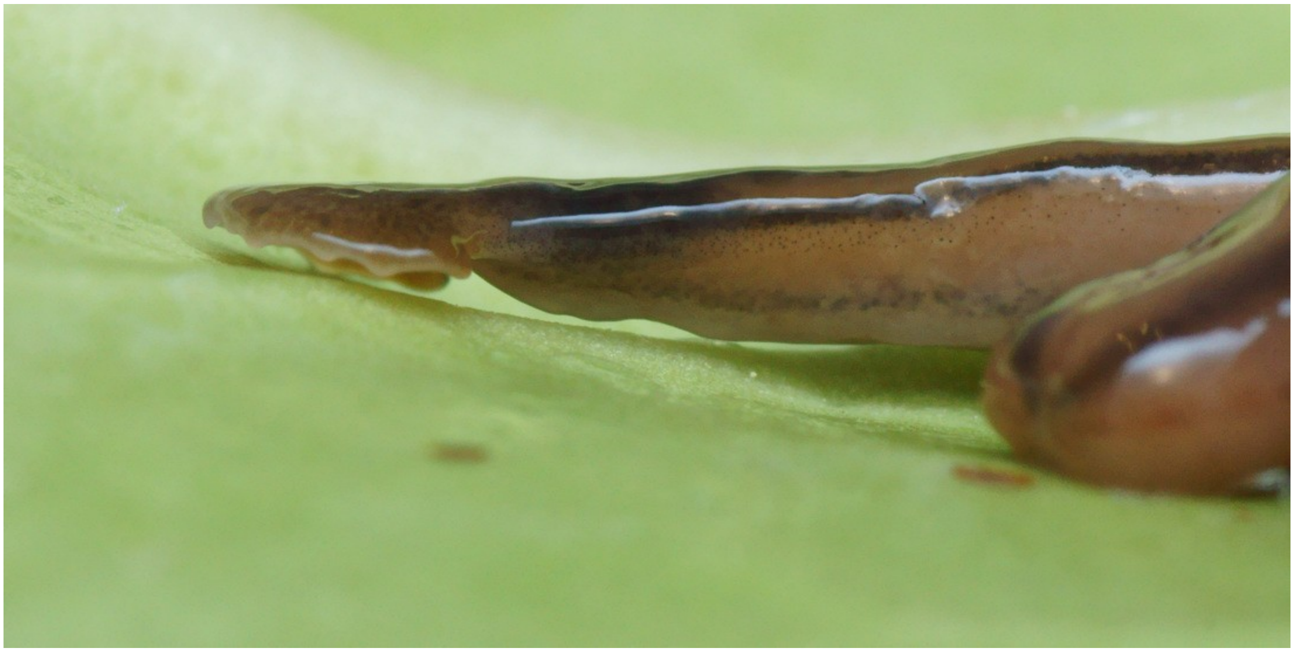

Figure 6. *Bipalium kewense*, morphologie générale, surface ventrale.

La bande du cou transversale foncé est incomplètement ventrale, et les bandes diffuses gris-violacé paires délimitent la sole rampante qui est blanc cassé. La position de la bouche est indiquée par *, et la position approximative du pharynx protrusible dans le corps est évidente sous l'aspect d'une zone pâle de chaque côté de la bouche. Photo par Pierre Gros

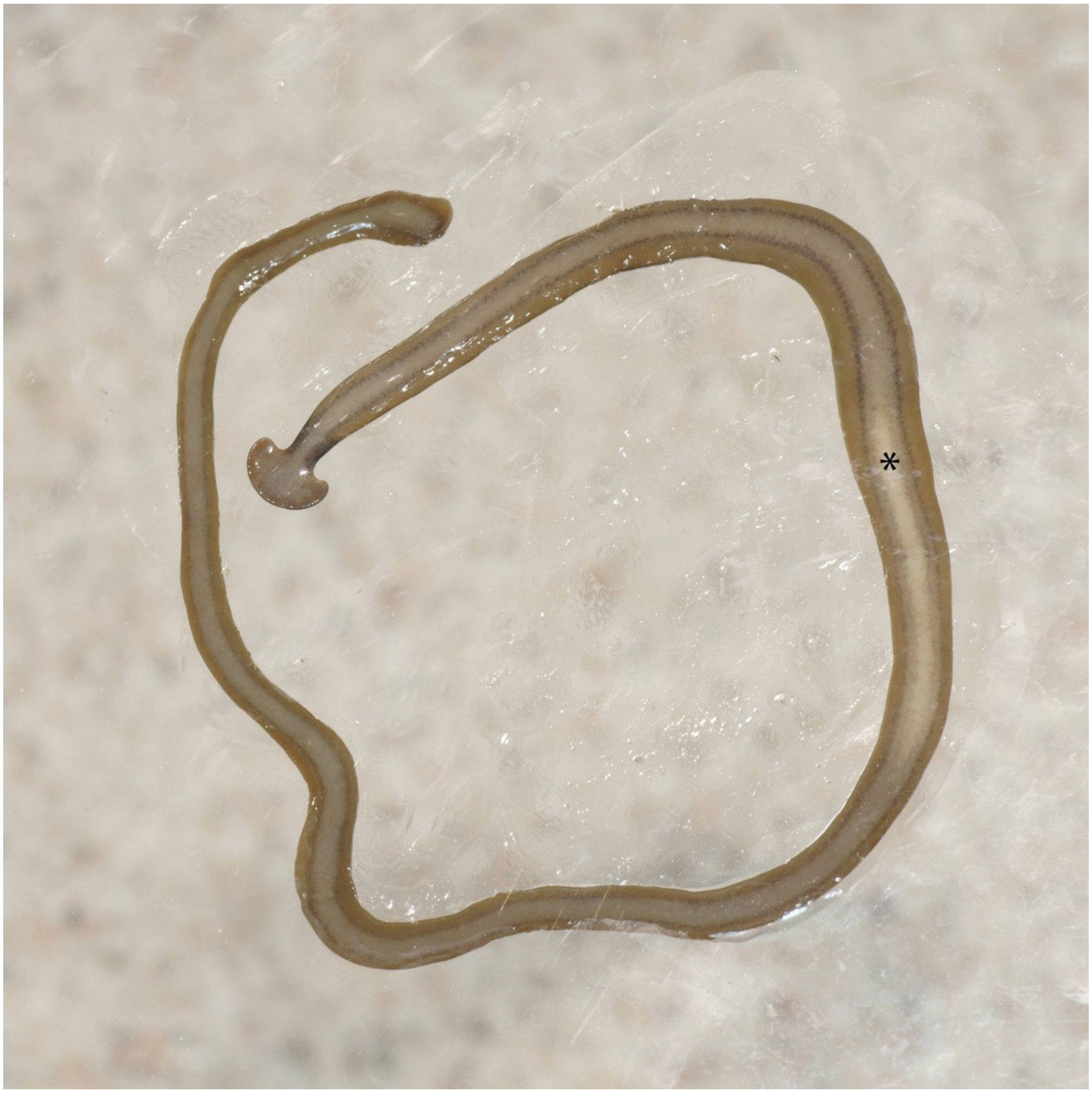

Figure 7. *Bipalium kewense*, prédation sur un ver de terre.

Le ver plat initie ici le processus de « coiffage » de l'extrémité antérieure du ver de terre. Les réactions observées de la proie suggèrent que c'est à ce stade que le Plathelminthe sécrète une toxine pour réduire la mobilité des proies (Stokes et al., 2014). Le Plathelminthe produit également des sécrétions à partir de sa plaque frontale et de son corps qui adhèrent à la proie, malgré les mouvements souvent violents et soudains de ce dernier au cours de cette phase de capture. Photo par Pierre Gros.

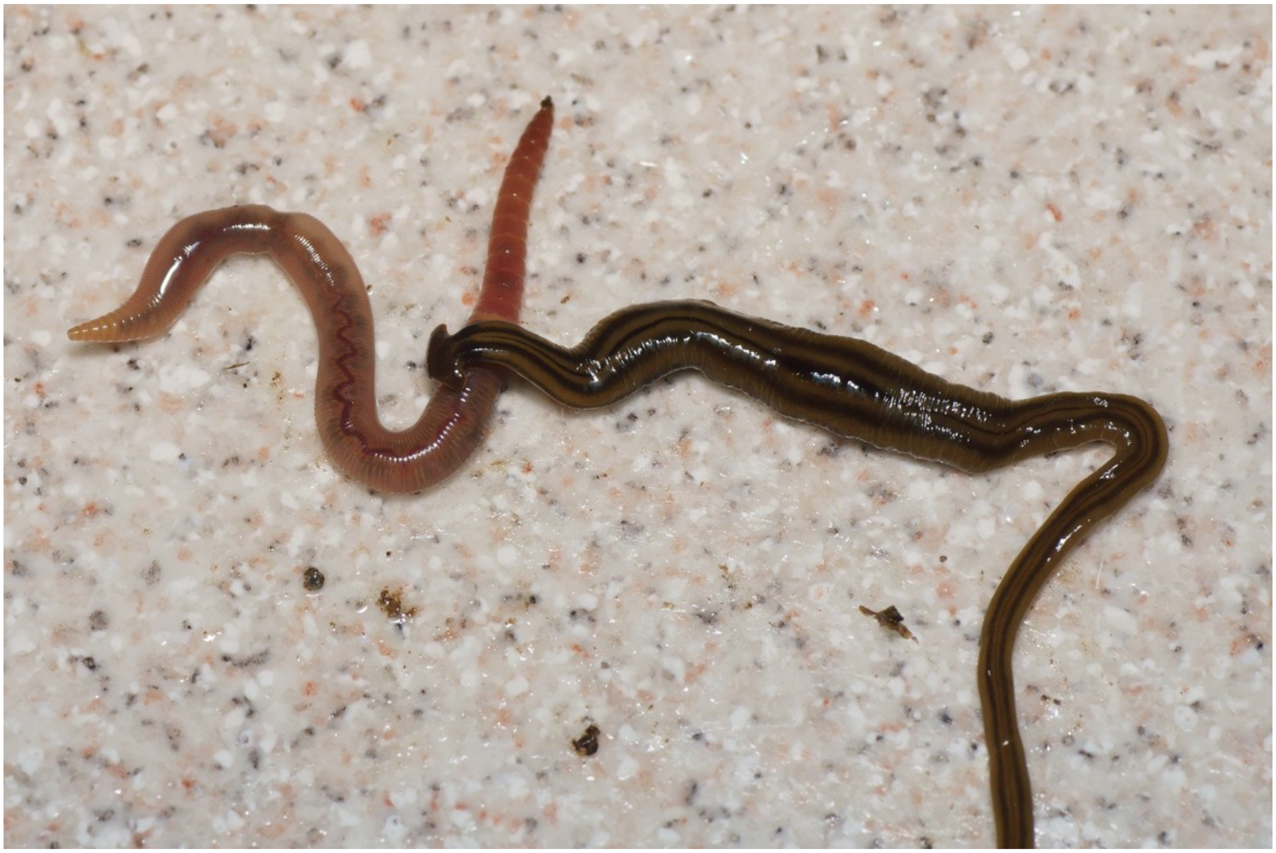

Figure 8. *Bipalium kewense*, reproduction par scissiparité.

La photographie montre un ver adulte (en haut) et le fragment qui vient de se séparer (en bas). Un à deux jours après l'alimentation, le processus de fission se manifeste d'abord par un léger pincement du corps, à une distance de l'ordre de 1-2 cm de l'extrémité de la queue. La rupture se produit lorsque l'extrémité de la queue adhère au substrat et que le reste du Plathelminthe s'éloigne. La reproduction sexuelle en dehors de leur habitat naturel est limitée aux individus qui sont en extérieur dans les climats tropicaux ou subtropicaux. Ailleurs, ils se reproduisent asexuellement. Les liens entre la sexualité et le climat, et le passage de la scissiparité à la production de cocons d'œufs, indiquent que plusieurs facteurs interagissent, notamment la disponibilité de la nourriture et la variabilité climatique (Winsor et al. 2004). Photo par Pierre Gros.

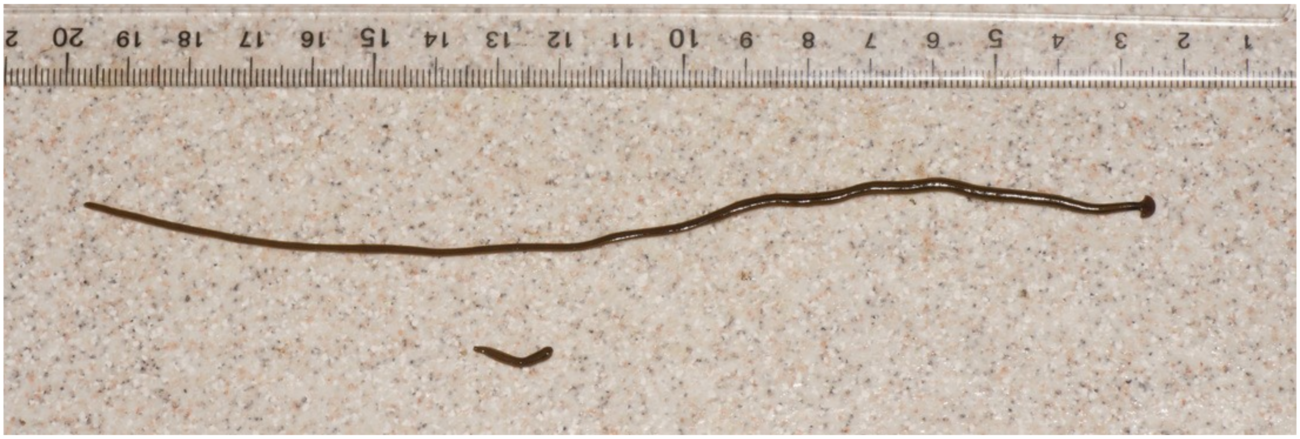

Figure 9. *Bipalium kewense*, reproduction par scissiparité - le fragment libéré de la queue.

Le fragment libéré de la queue est immédiatement mobile. Il développe une tête et un pharynx dans les 7-10 jours, et dans 2-3 semaines, il est adulte dans sa forme et son comportement (Connella & Stern 1969). On considère que la reproduction asexuée chez *B. kewense* et chez certains autres Plathelminthes est à la base du succès de colonisation de ces espèces (Hyman 1951, p. 163). Photo par Pierre Gros.

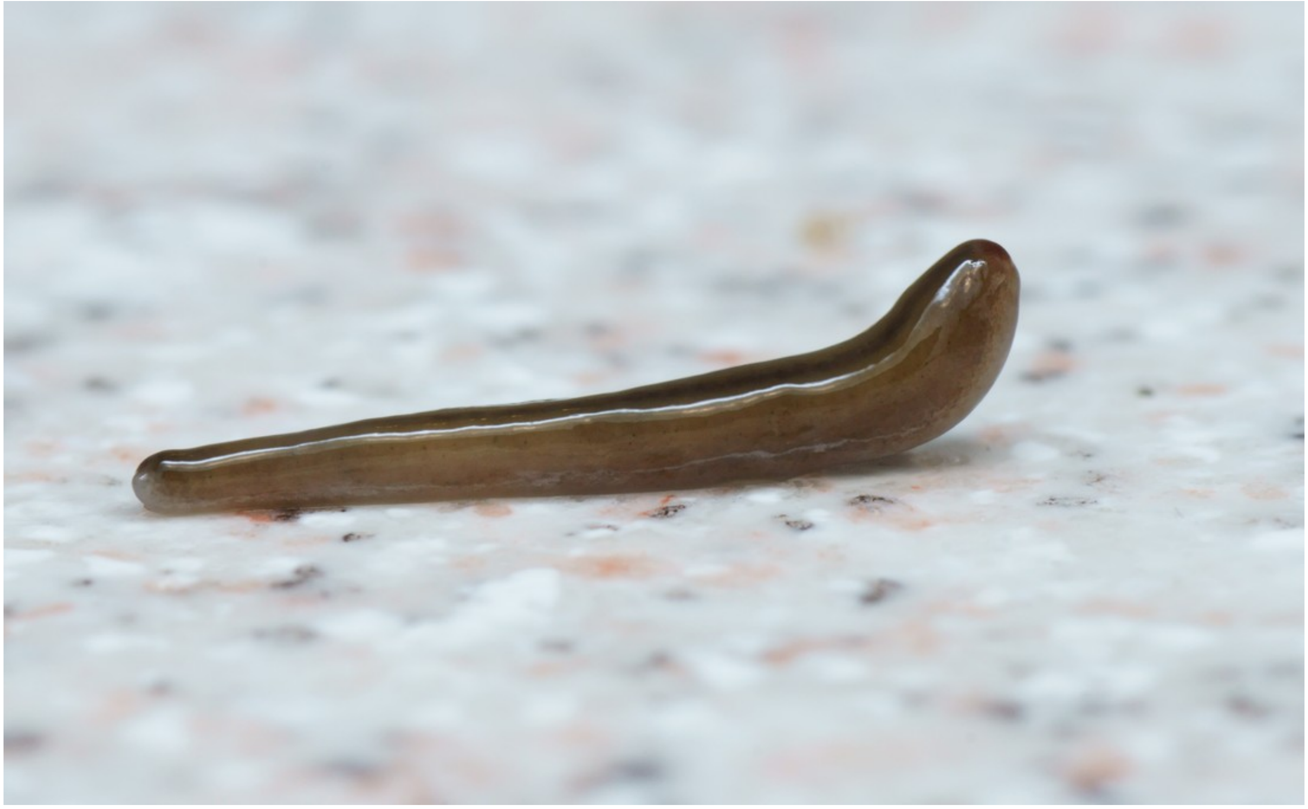

Figure 10. *Diversibipalium multilineatum*, morphologie générale.

Aspect dorsal avec une vue partielle de la surface ventrale. La bande médiane dorsale sombre s'étend sur la plaque frontale, et la plaque frontale est plus arrondie que la plaque frontale falciforme de *Bipalium kewense*. Notez l'extrémité postérieure arrondie du corps indiquant la reproduction par scissiparité. Photo par Pierre Gros.

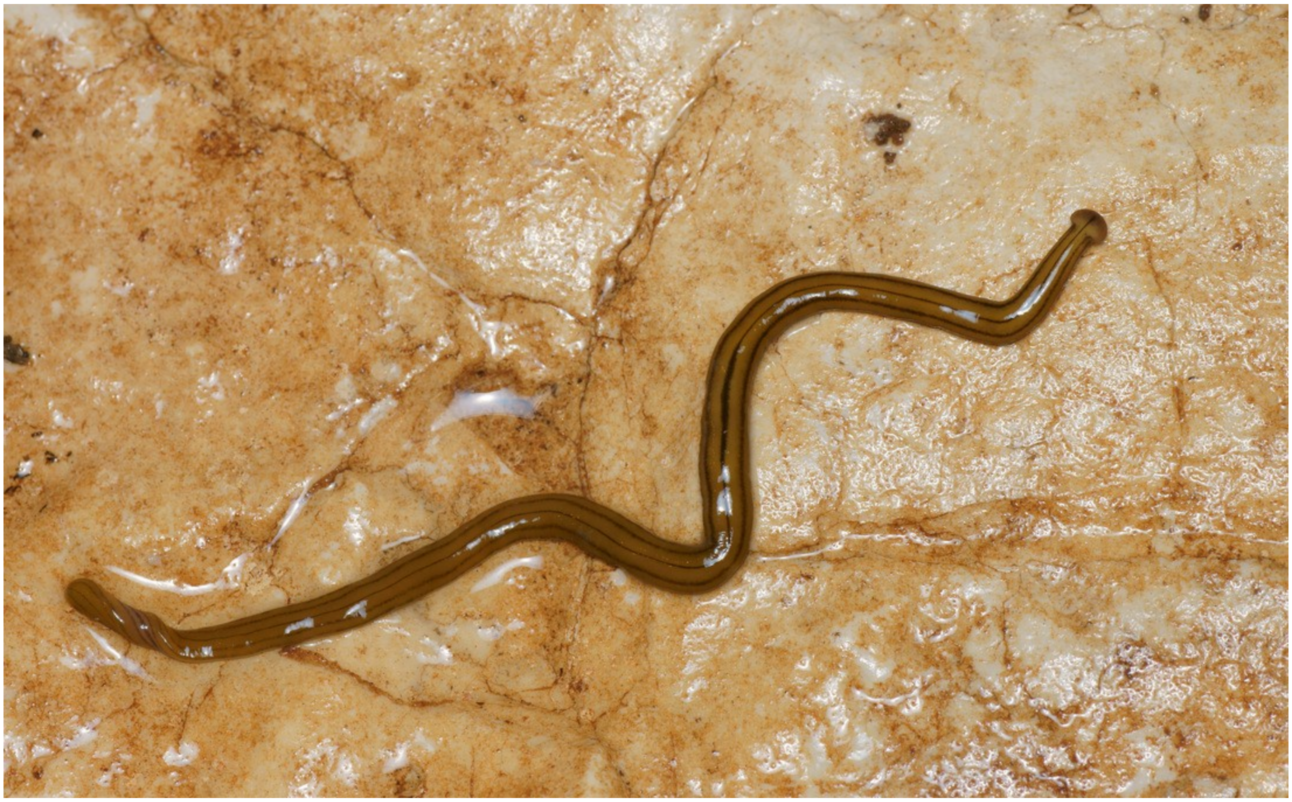

Figure 11. *Diversibipalium multilineatum*, plaque frontale.

Sur la plaque frontale, la bande dorsale médiane noire commence au tiers antérieur de la plaque frontale et a une forme oblancéolée caractéristique prononcée. Photo par Pierre Gros.

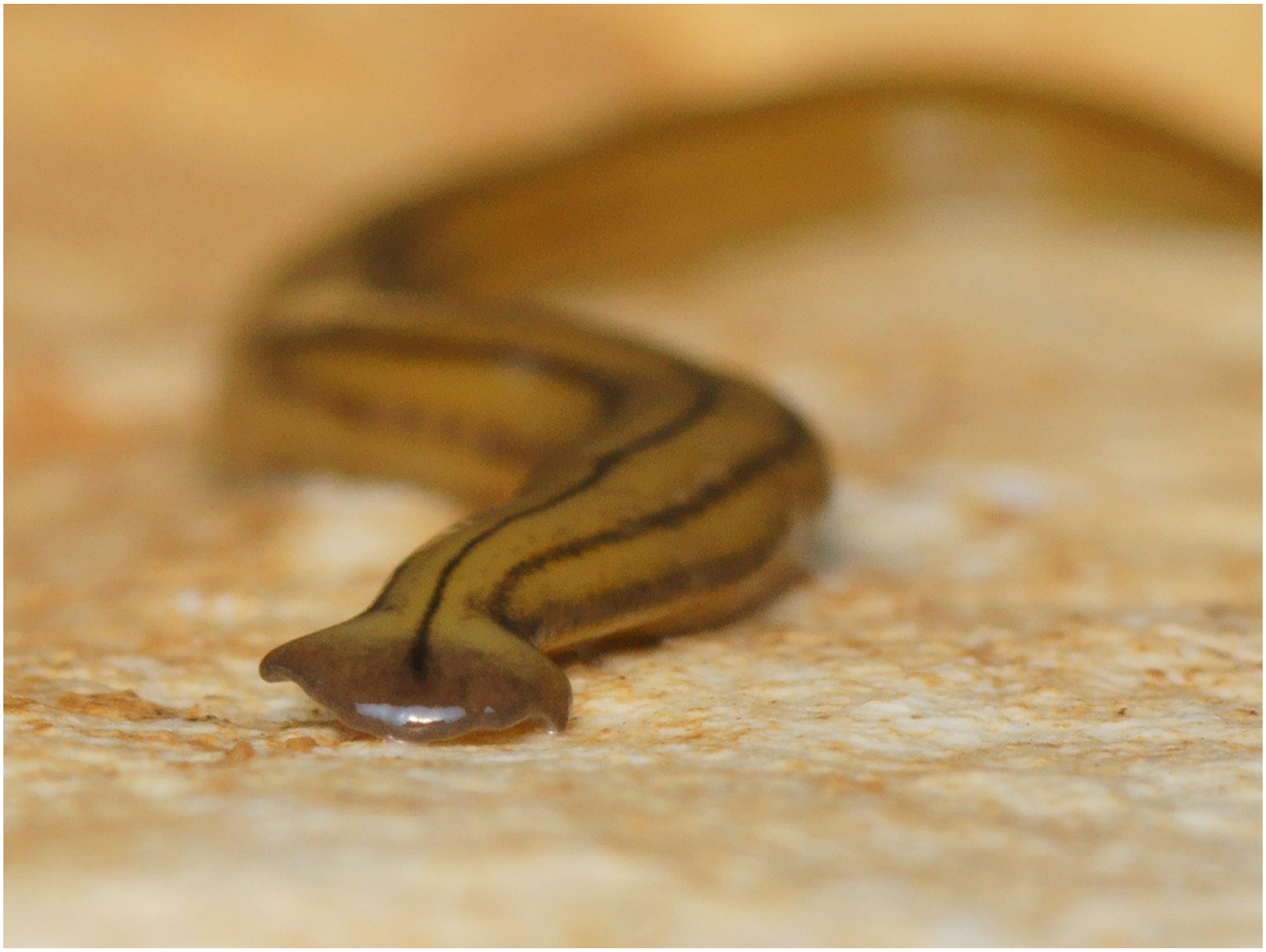

Figure 12. *Diversibipalium multilineatum*, morphologie générale, extrémité antérieure.

Les bandes dorsales latérales commencent immédiatement derrière la plaque frontale. Il n'y a pas de bande sombre transversale (ou « collier »). Photo par Pierre Gros.

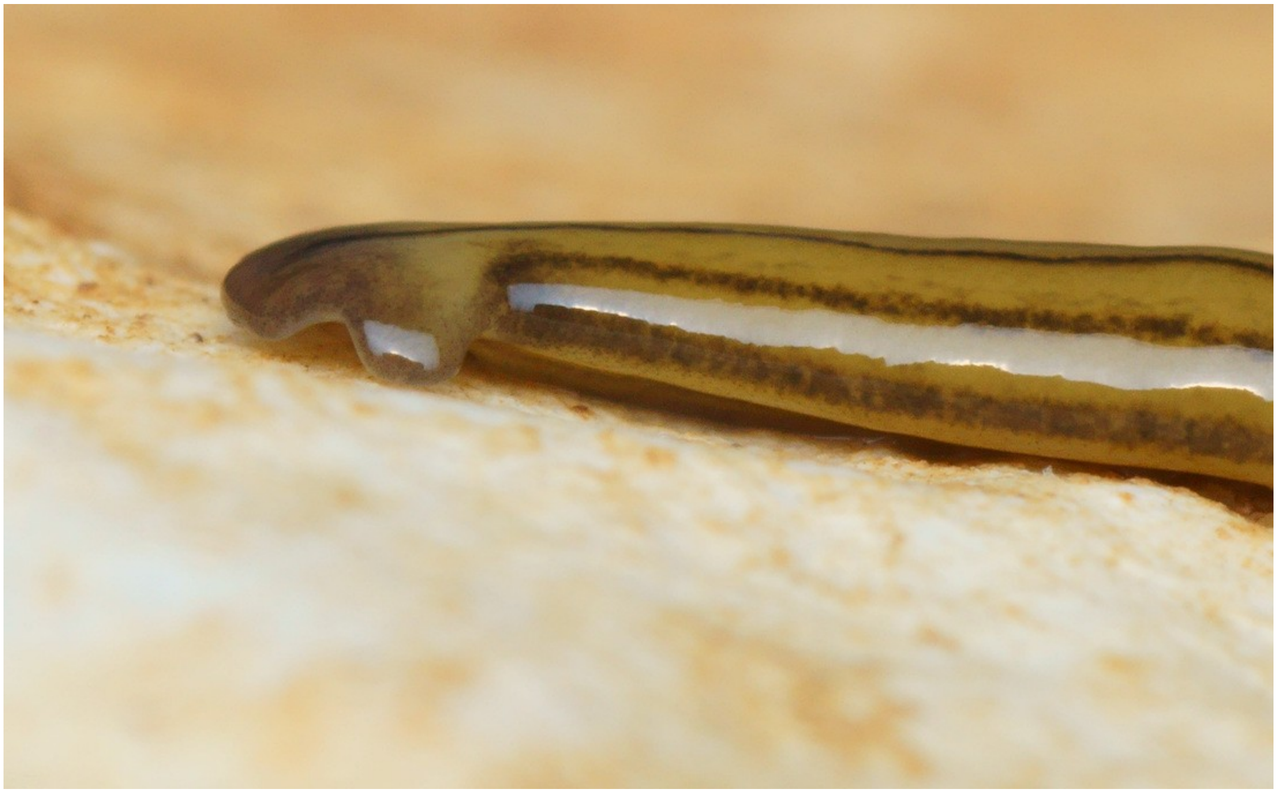

Figure 13. ***Diversibipalium multilineatum*, morphologie de la plaque frontale ventrale.**

La fine bande sombre, généralement discontinue, de la partie centrale de la face ventrale s'étend du tiers antérieur de la plaque frontale à l'extrémité postérieure. Il y a aussi de faibles indications sur les débuts des bandes latérales paires ventrales, sous la plaque frontale. Photo par Pierre Gros.

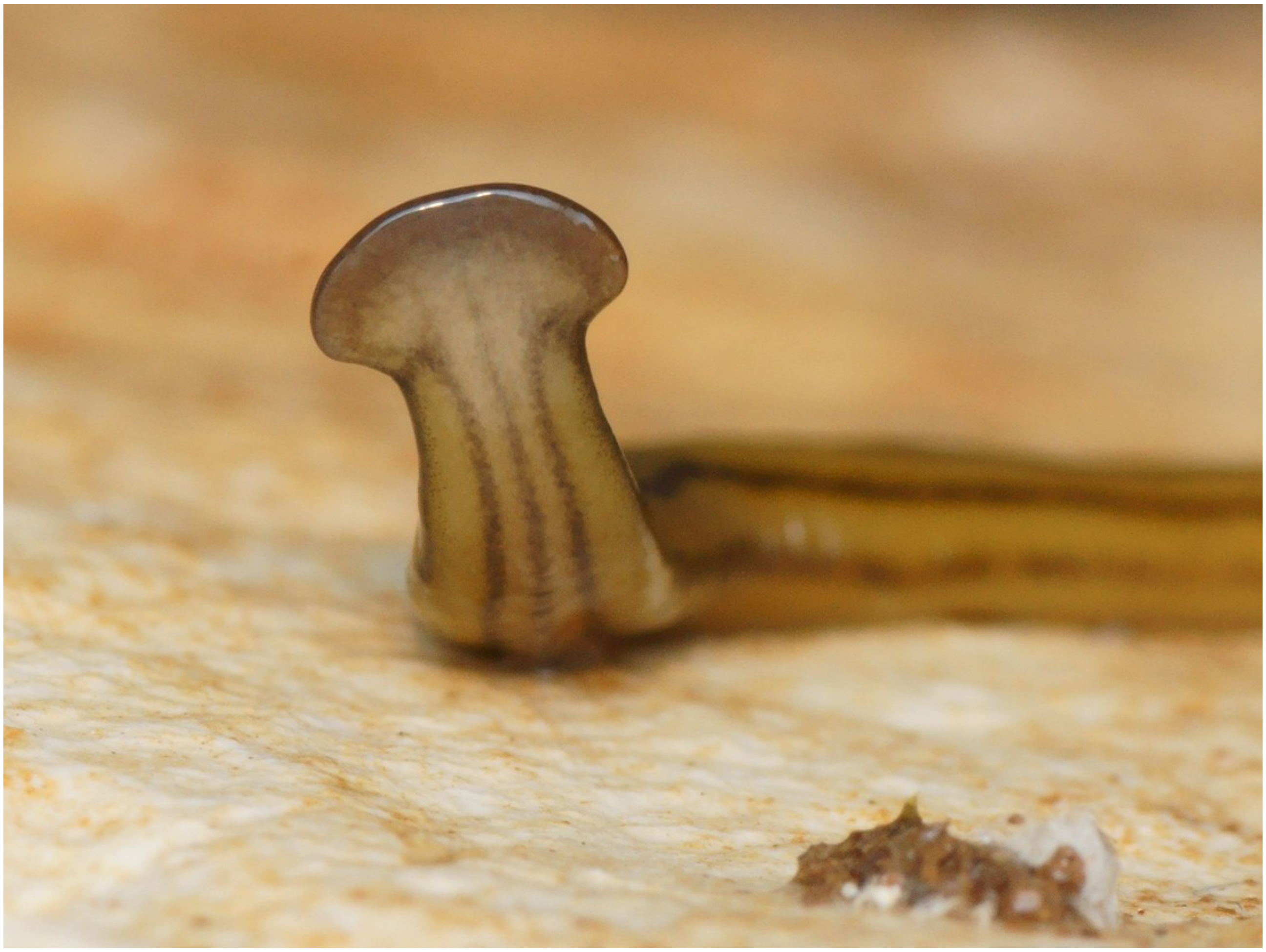

Figure 14. *Diversibipalium multilineatum*, morphologie générale, surface ventrale.

Les trois bandes longitudinales sombres commencent au « cou » et s'étendent sur toute la longueur du corps. La position de la bouche est indiquée par *, et la position approximative du pharynx protrusible dans le corps est mise en évidence par l'aspect diffus de la bande médiane dans cette région. Photo par Pierre Gros.

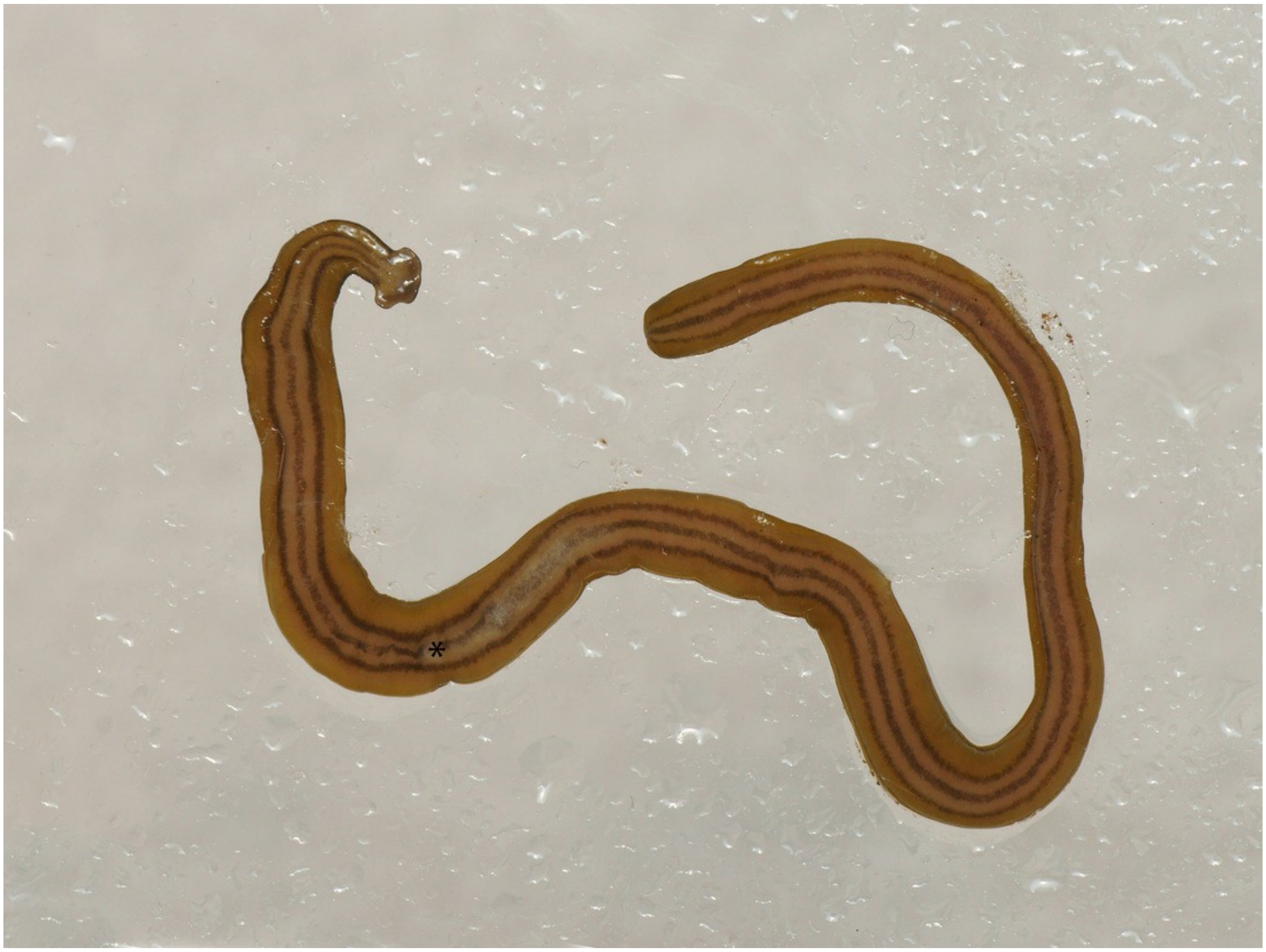

Figure 15. *Bipalium vagum*. Spécimen de Guyane française.

Le marquage dorsal sur ce spécimen est typique de l'espèce. Notez les taches sombres sur la plaque frontale, le tour de cou continu, les rayures médianes noires, les bandes latérales paires marron et la pointe caudale noire. Photo par Sébastien Sant, Parc Amazonien de Guyane.

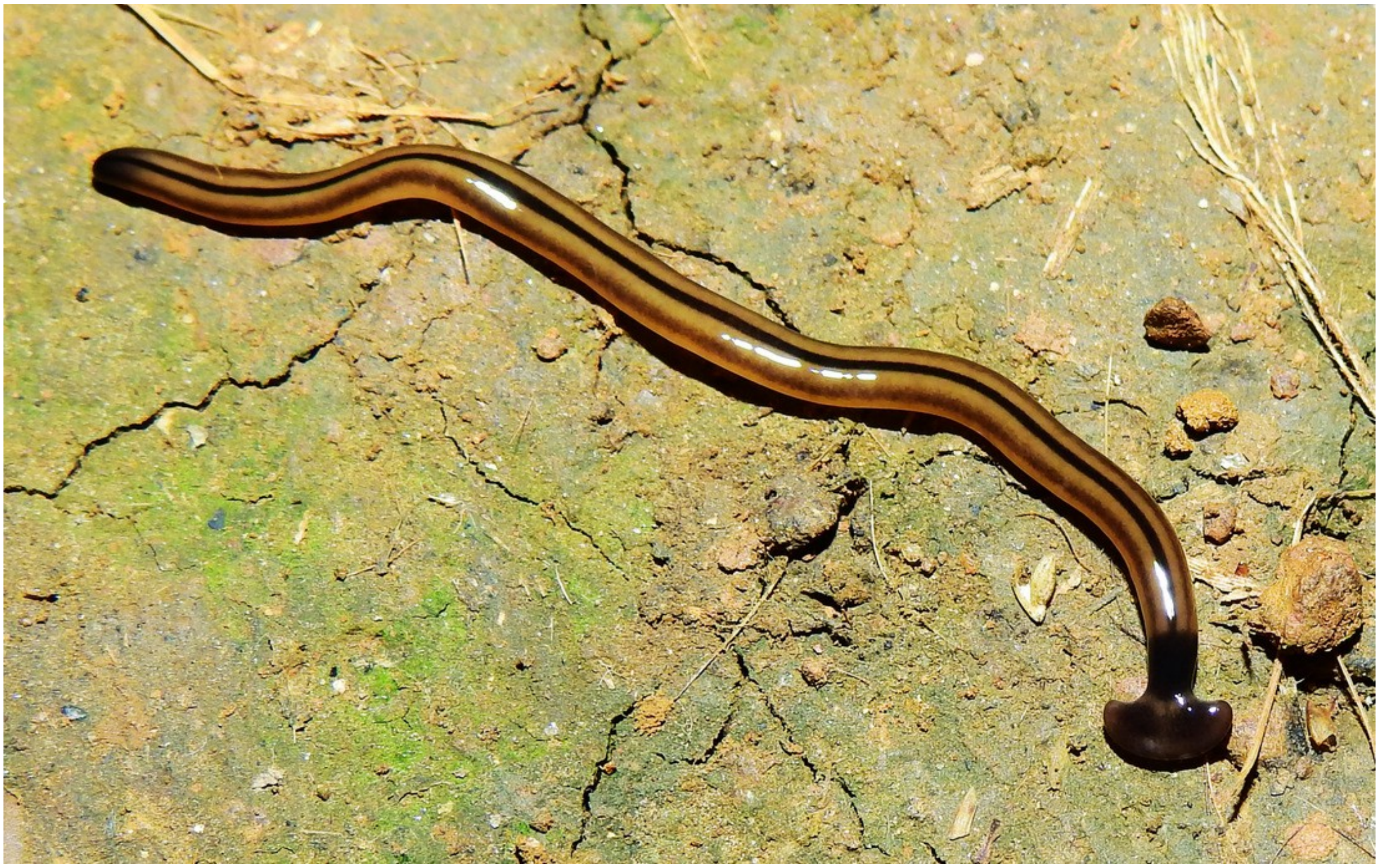

Figure 16. *Bipalium vagum*. Spécimen de Guadeloupe, Antilles.

Ce spécimen présente une pigmentation très claire, notamment sur la plaque frontale, les bandes latérales marron indistinctes et la pointe caudale. Photo par Pierre et Claudine Guezennec.

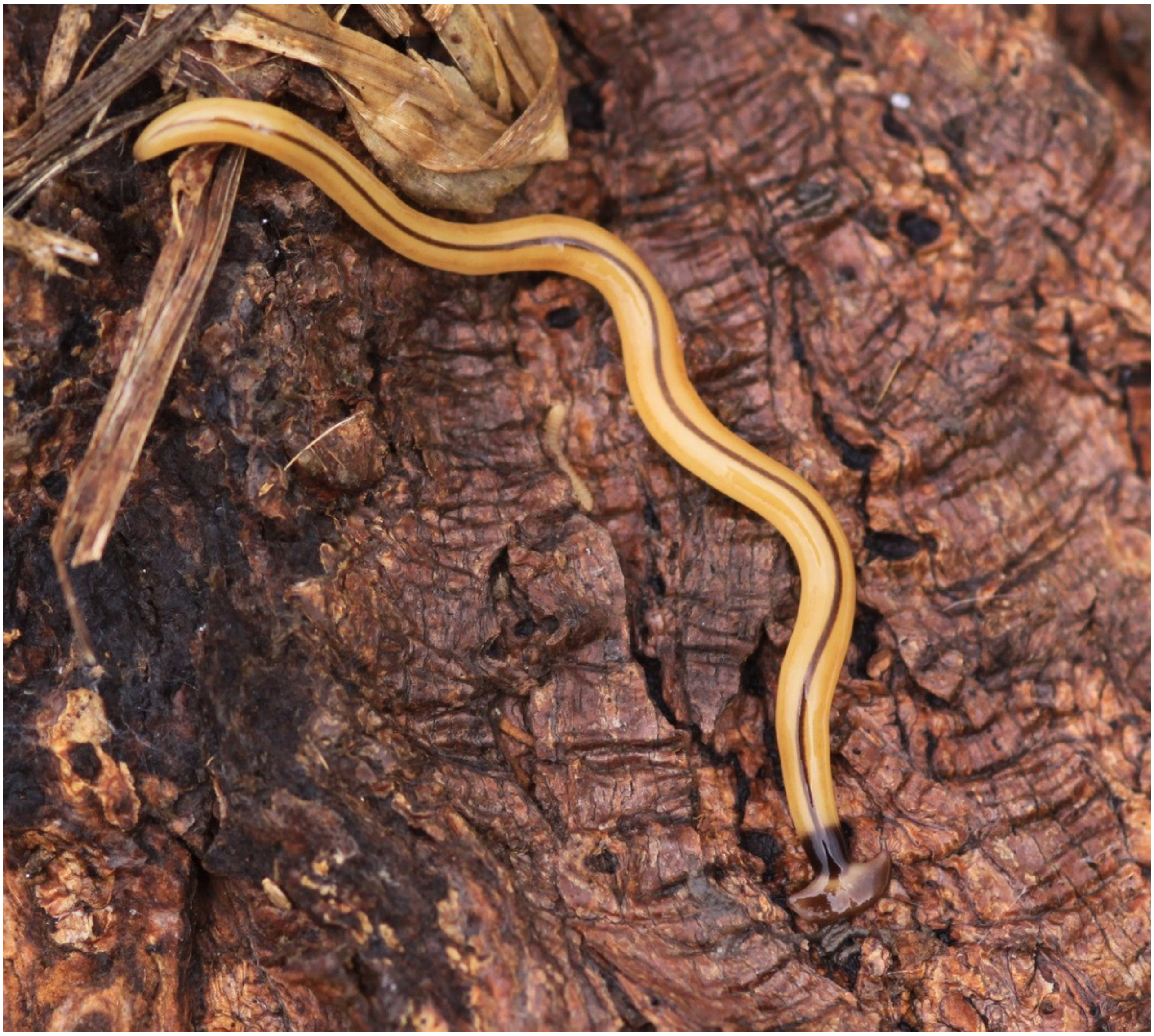

Figure 17. *Bipalium vagum*. Spécimen de Martinique, Antilles.

Dans ce spécimen, la plaque frontale présente une pigmentation marquée de sorte qu'elle apparaît presque noire. Photo par Mathieu Coulis.

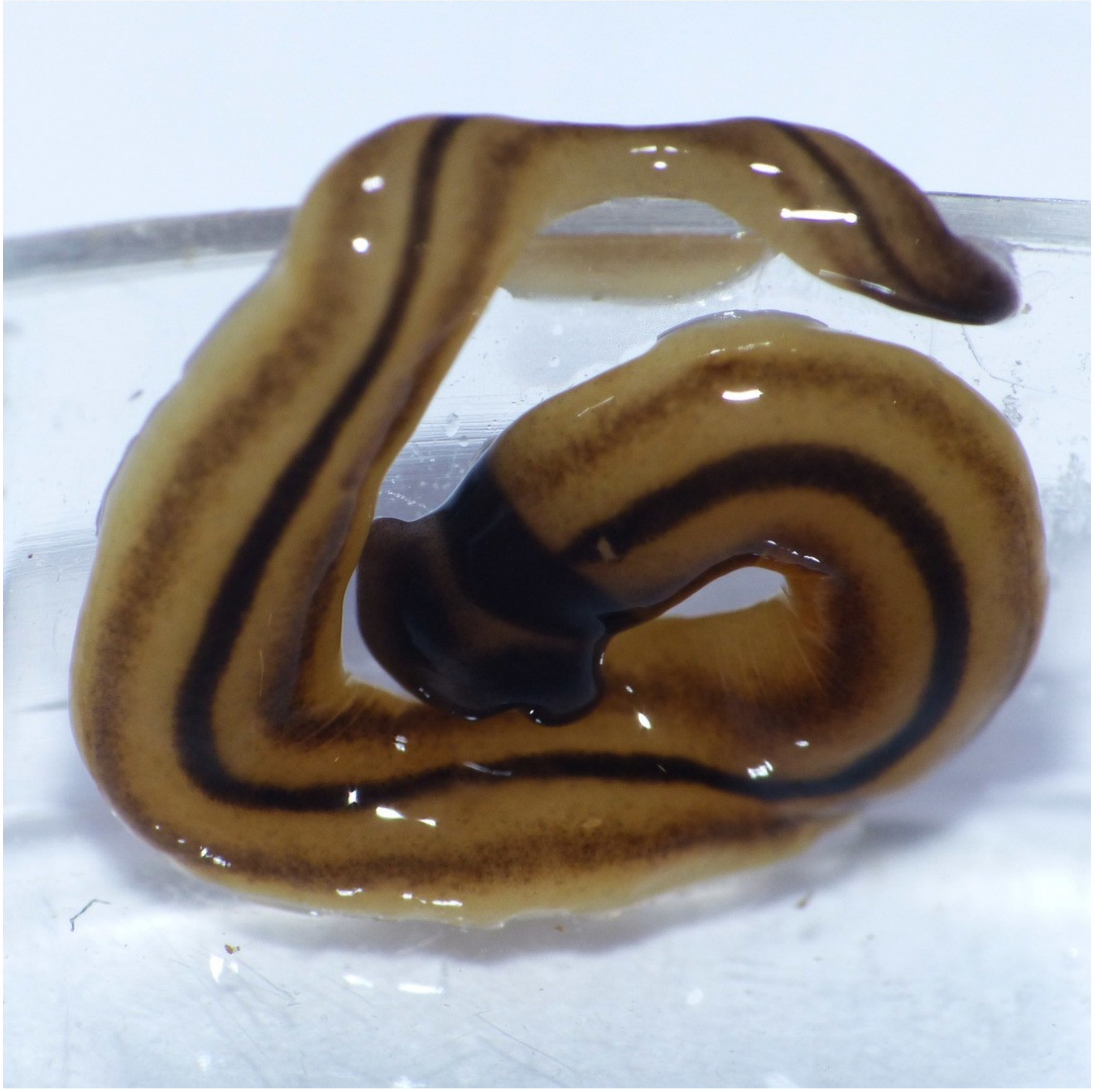

Figure 18. *Bipalium vagum*. Spécimen de La Réunion, Océan Indien.

Ce spécimen présente des marques typiques de l'espèce. Les taches foncées appariées sur la plaque frontale et la pointe caudale pigmentée foncée sont clairement visibles. Photo par Dominique Martiré.

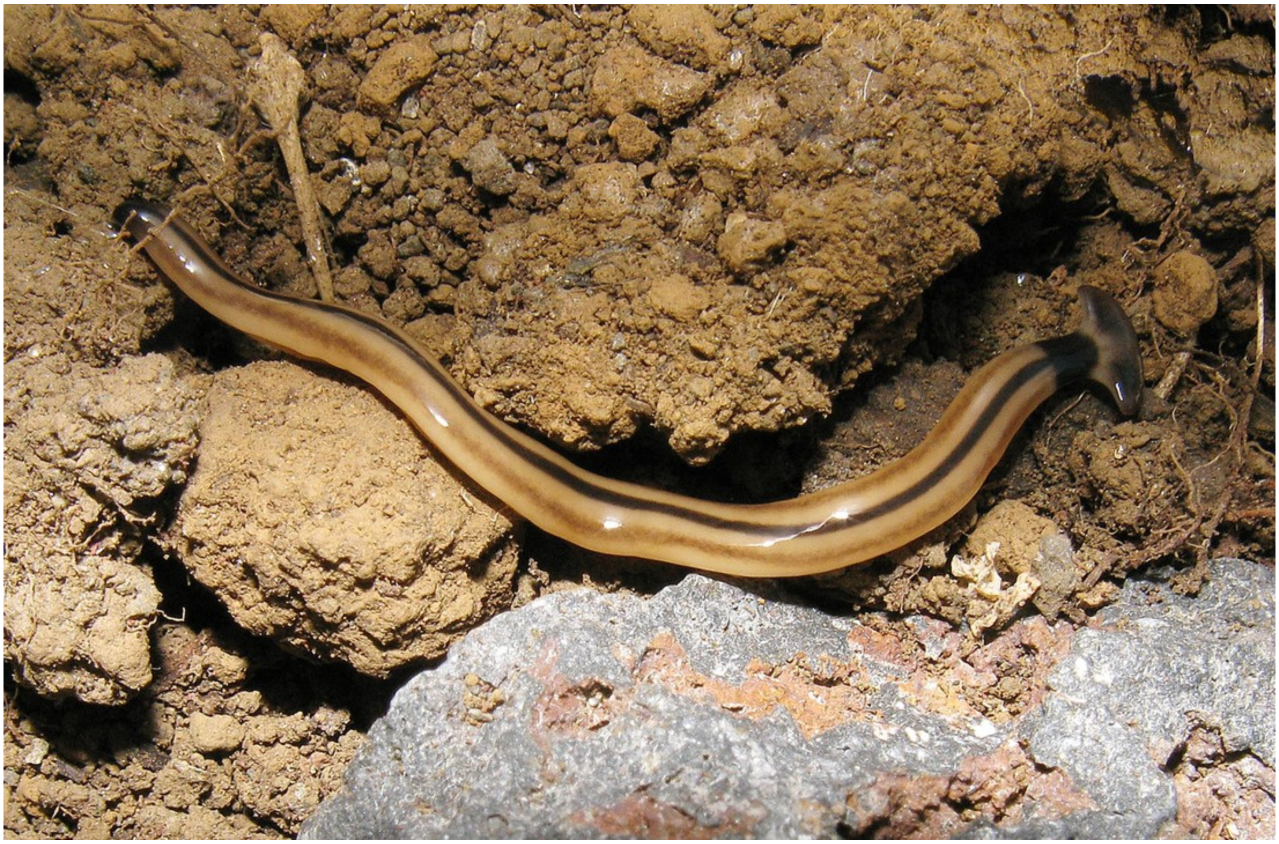

Figure 19. *Diversibipalium* sp. « noir » de France métropolitaine.

Dessins tirés de photographies de trois spécimens vivants en vue dorsale. La couleur de fond dorsale des spécimens est noire, sans aucune trace de rayures dorsales. L'échelle (10 mm) est valable pour les deux échantillons à gauche, l'échantillon à droite n’a pas d'échelle.

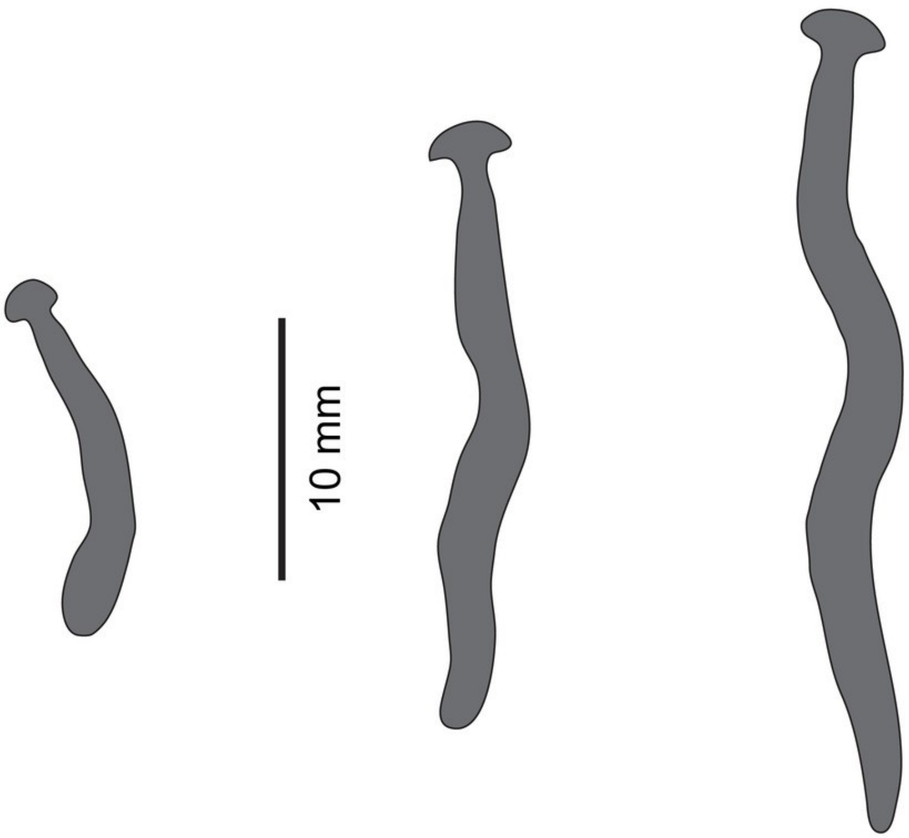

Figure 20. *Diversibipalium* sp. « noir » de France métropolitaine, spécimen conservé.

Spécimen MNHN JL090. Aspect dorsolatéral montrant le pharynx partiellement saillant. Photo par Jean-Lou Justine.

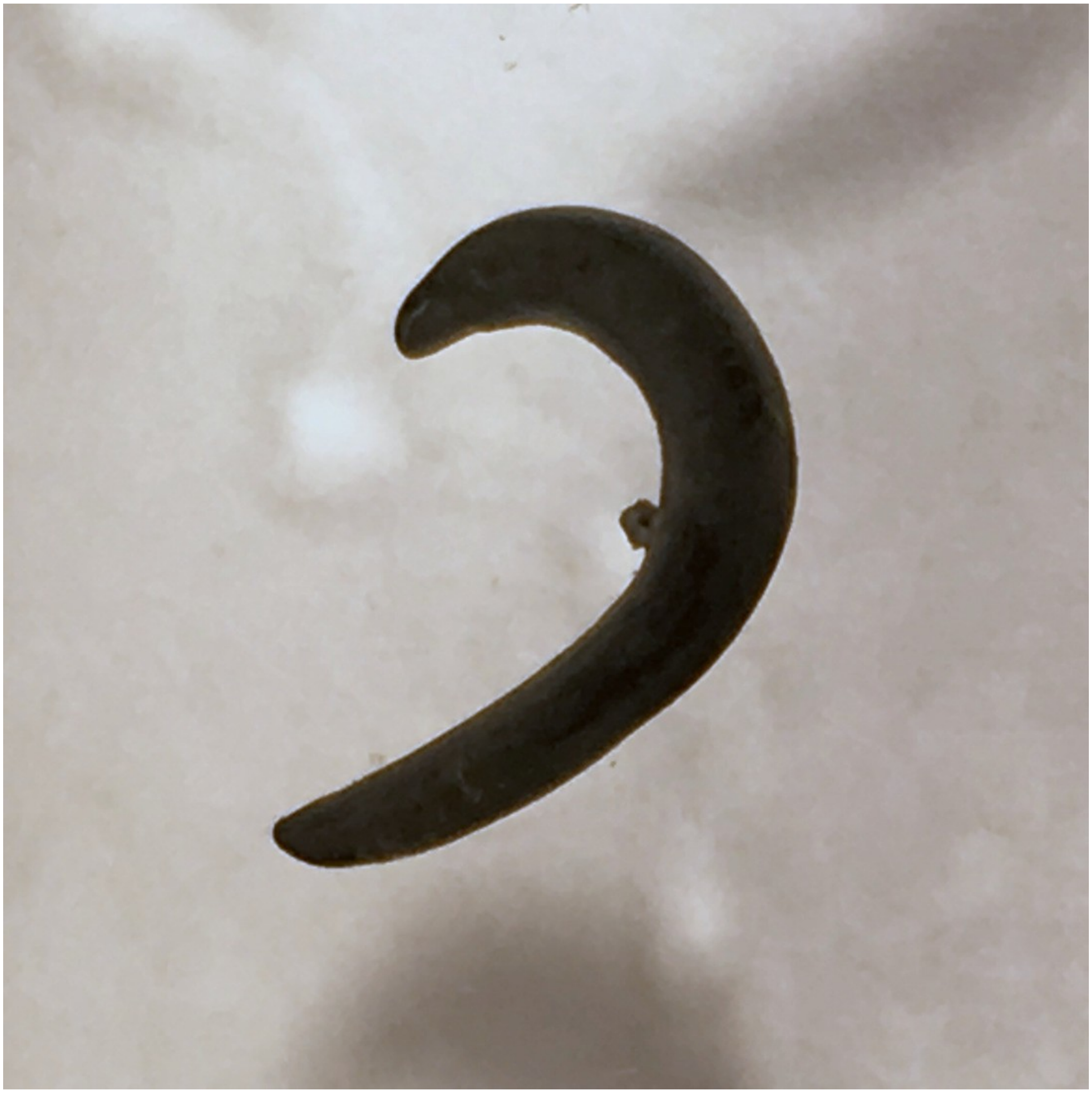

Figure 21. *Diversibipalium* sp. « noir » de France métropolitaine, spécimen conservé.

Spécimen MNHN JL090. Aspect ventral. La couleur ventrale générale est grise, avec la sole rampante un ton plus clair. Le pharynx est légèrement en saillie de la bouche, et le gonopore est évident sous forme d'une petite fente transversale blanche sur la sole rampante, environ 2 mm en arrière de la bouche. L'échelle est en mm. Photo par Jean-Lou Justine.

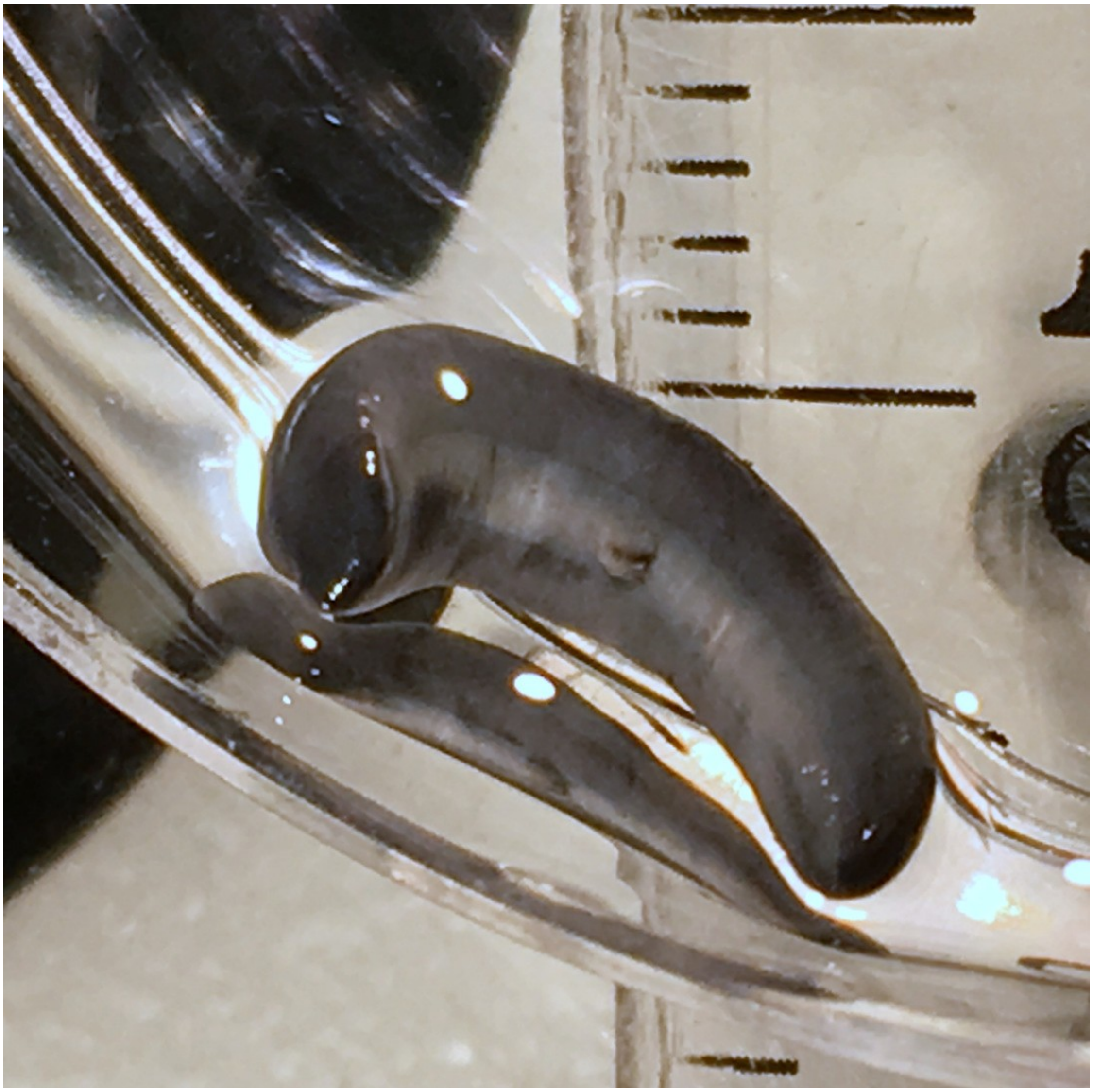

Figure 22. *Diversibipalium* sp. « bleu » de Mayotte, Océan Indien, aspect dorsal.

La plaque frontale de ce petit Plathelminthe est de couleur brune, avec un dos bleu. Ce spécimen vivant mesure environ 45 mm de long. Photo par Benoît Duperron, Conservatoire Botanique National de Mascarin.

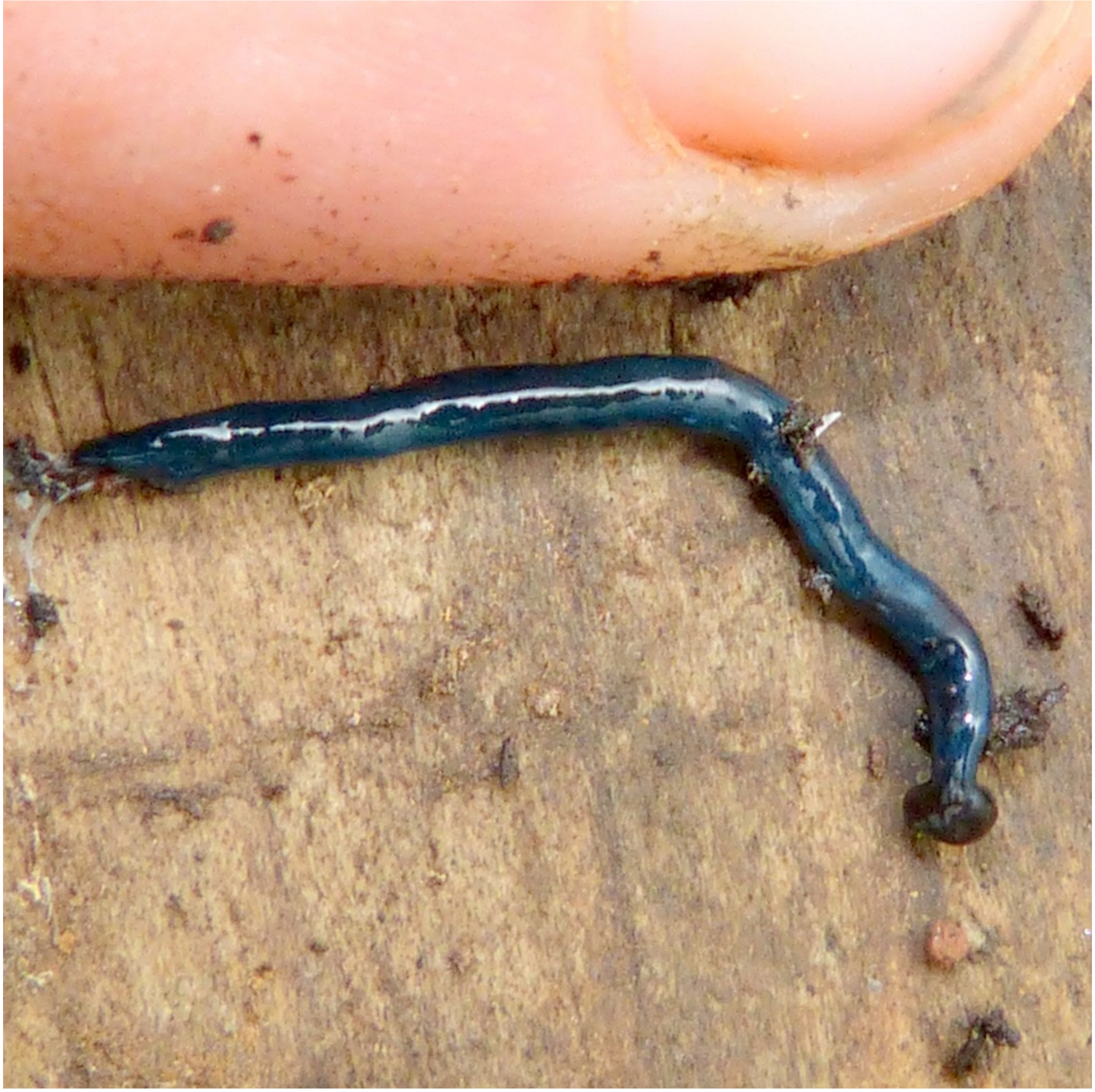

Figure 23. *Diversibipalium* sp. « bleu » de Mayotte, Océan Indien, aspect dorsal.

Spécimen MNHN JL282. La plaque frontale de ce petit Plathelminthe est une couleur brun rouille qui s'étend à quelques taches irrégulières sur le « cou ». La couleur de fond dorsale est un bleu-vert irisé (« turquoise foncé à paillettes »). Photo par Laurent Charles.

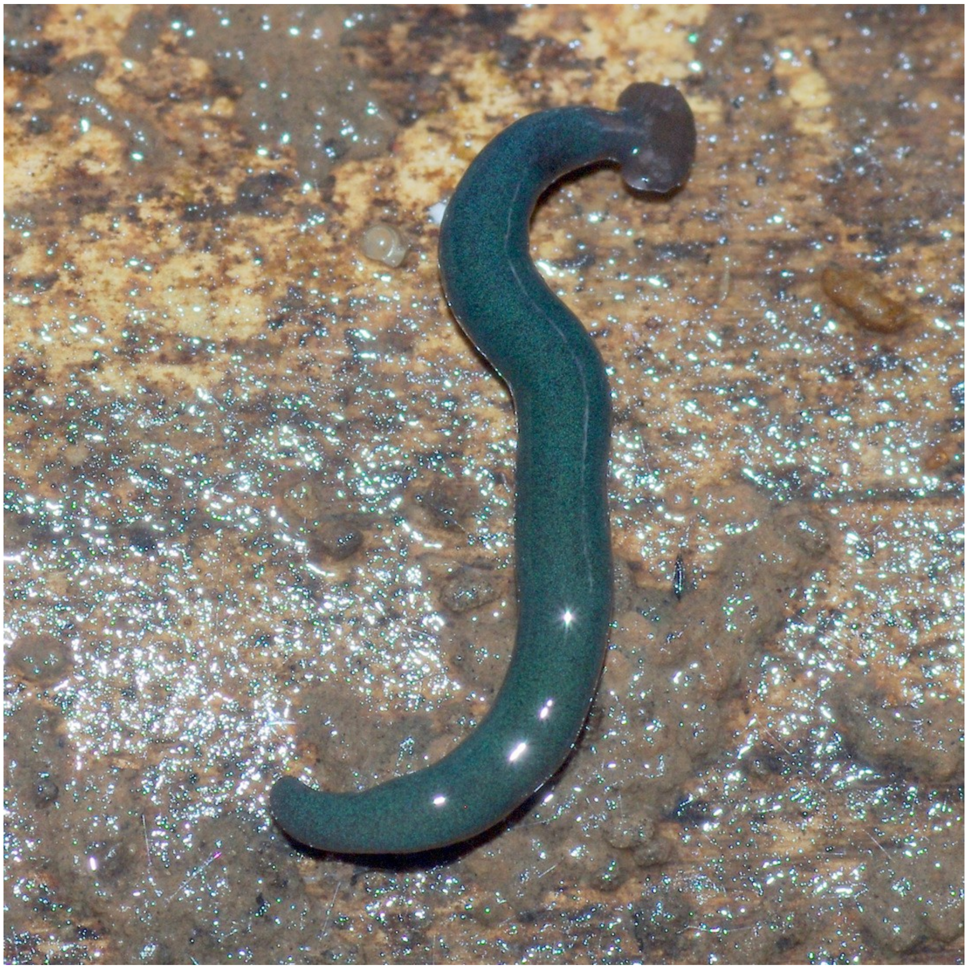

Figure 24. *Diversibipalium* sp. « bleu » de Mayotte, Océan Indien aspect dorsal.

Spécimen MNHN JL282. Comme pour Figure 25. Photo par Laurent Charles.

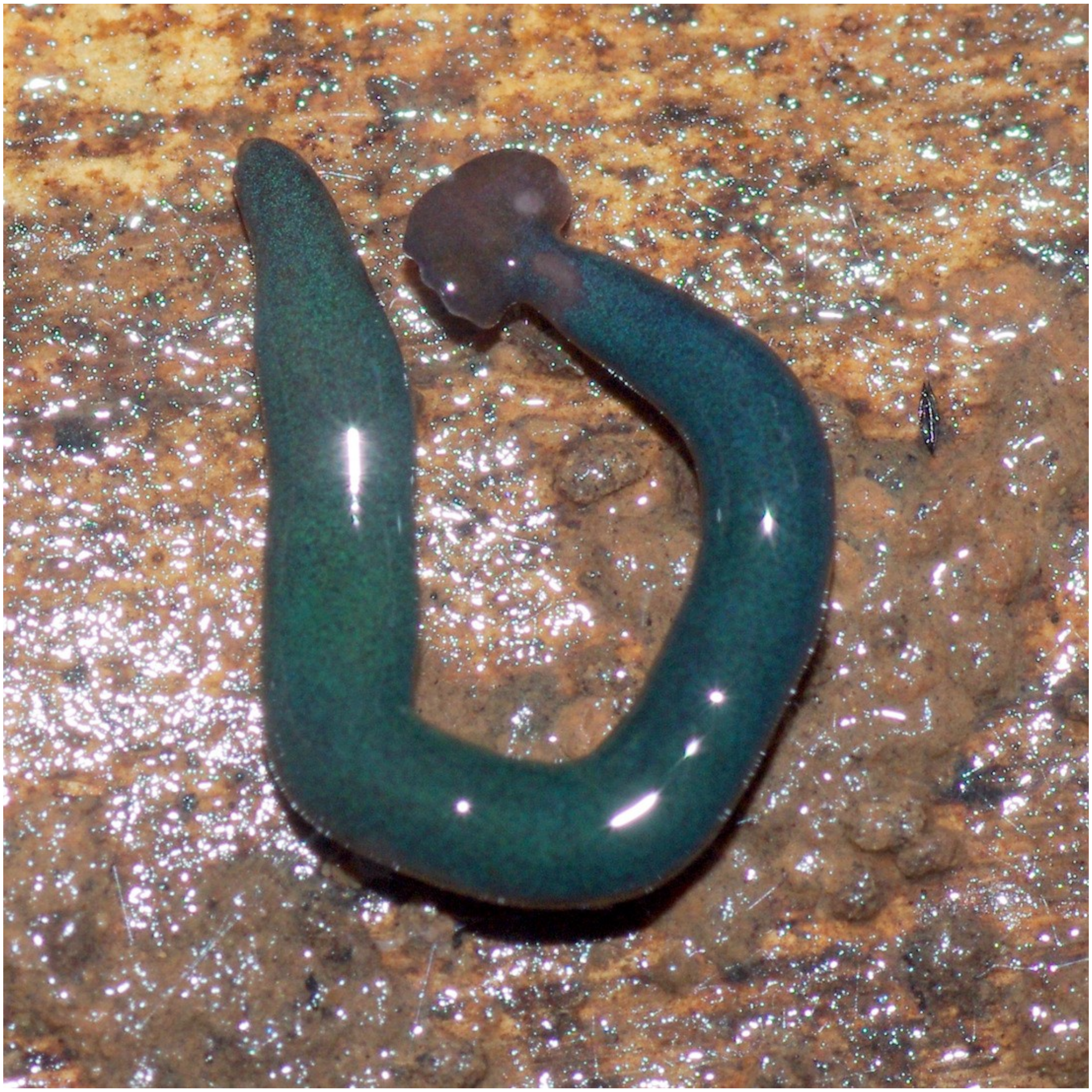

Figure 25. *Diversibipalium* sp. « bleu » de Mayotte, Océan Indien. Aspect dorsal d'un spécimen en régénération avec une extrémité antérieure endommagée.

Spécimen MNHN JL280. Sous un éclairage approprié, la couleur de l'échantillon prend une belle couleur verte presque métallique. L'irisation et la couleur bleu-vert sont perdues lors de la fixation, laissant le spécimen brun foncé. Photo par Laurent Charles.

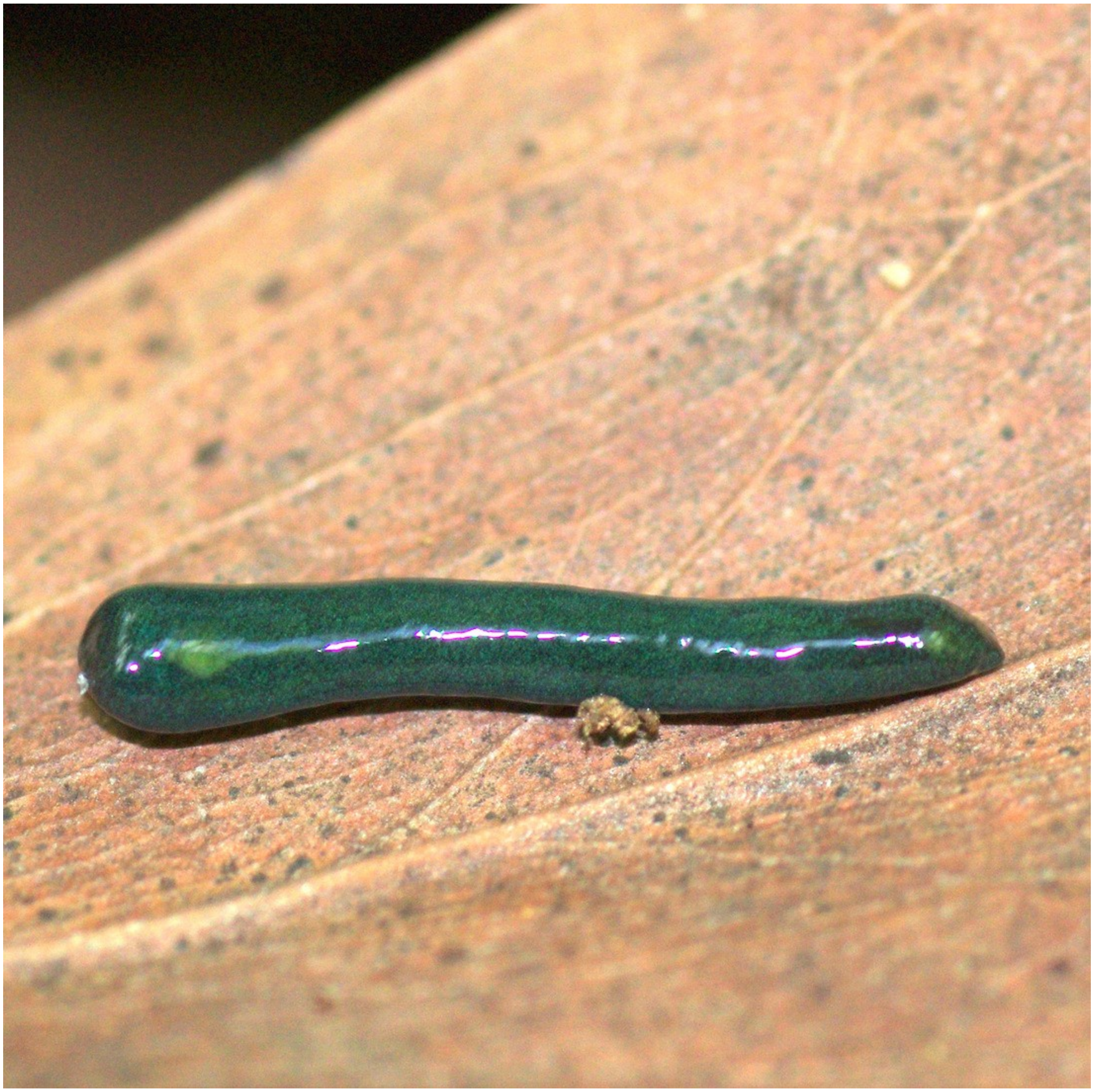

Figure 26. *Diversibipalium* sp. « bleu » de Mayotte, Océan Indien. Aspect dorsal d'un spécimen en régénération avec une extrémité antérieure endommagée.

Spécimen MNHN JL280. On peut voir une petite partie de la surface ventrale brune pigmentée avec la sole rampante médiane pâle. Photo par Laurent Charles.

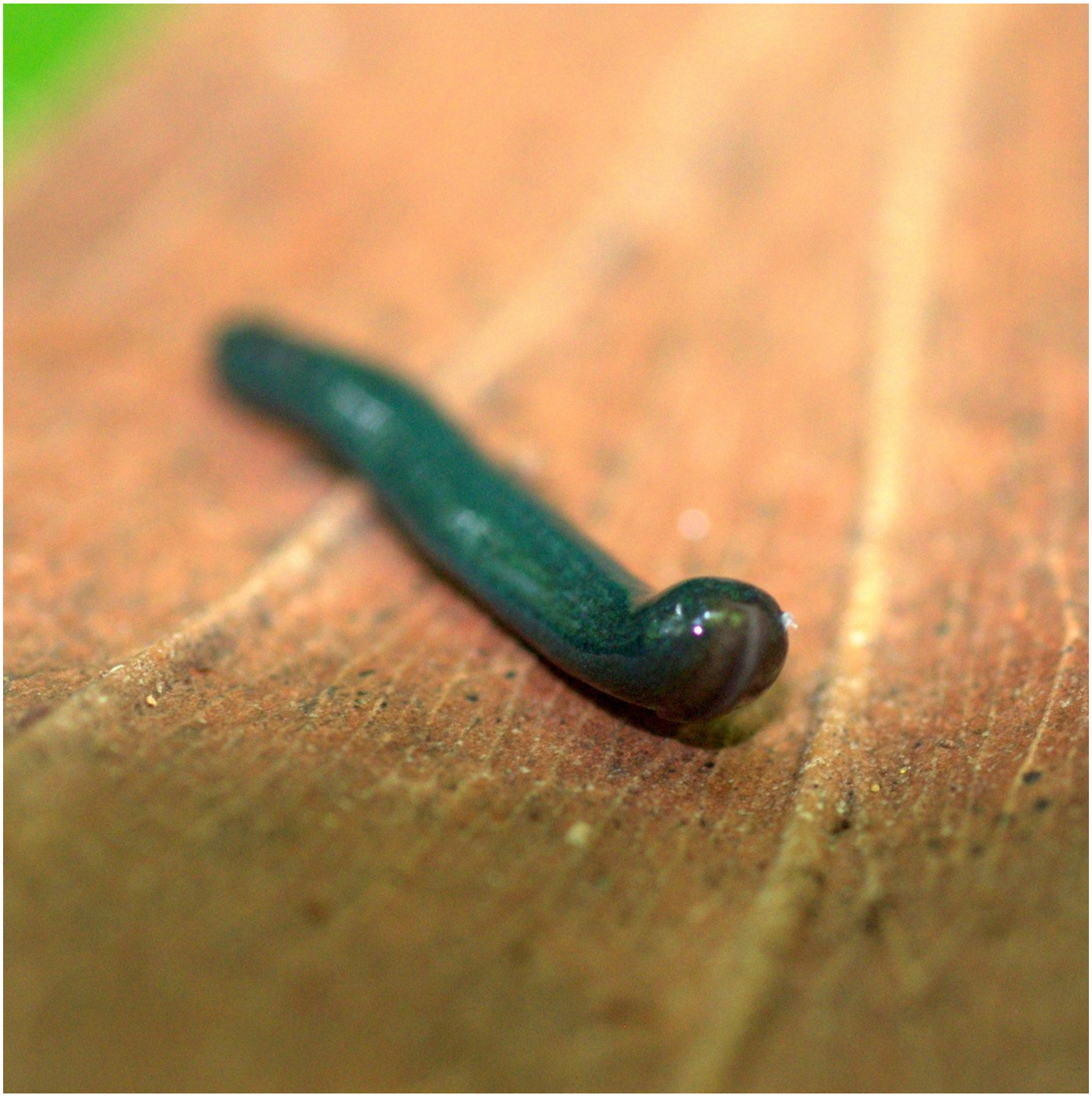

Figure 27. Carte de la partie sud-est de la France, montrant de nombreux nouveaux signalements de Plathelminthes bipaliinés.

Les noms des communes sont indiqués. La plupart des signalements proviennent du département des Pyrénées-Atlantiques, en particulier sa partie à faible altitude près de l'océan Atlantique.

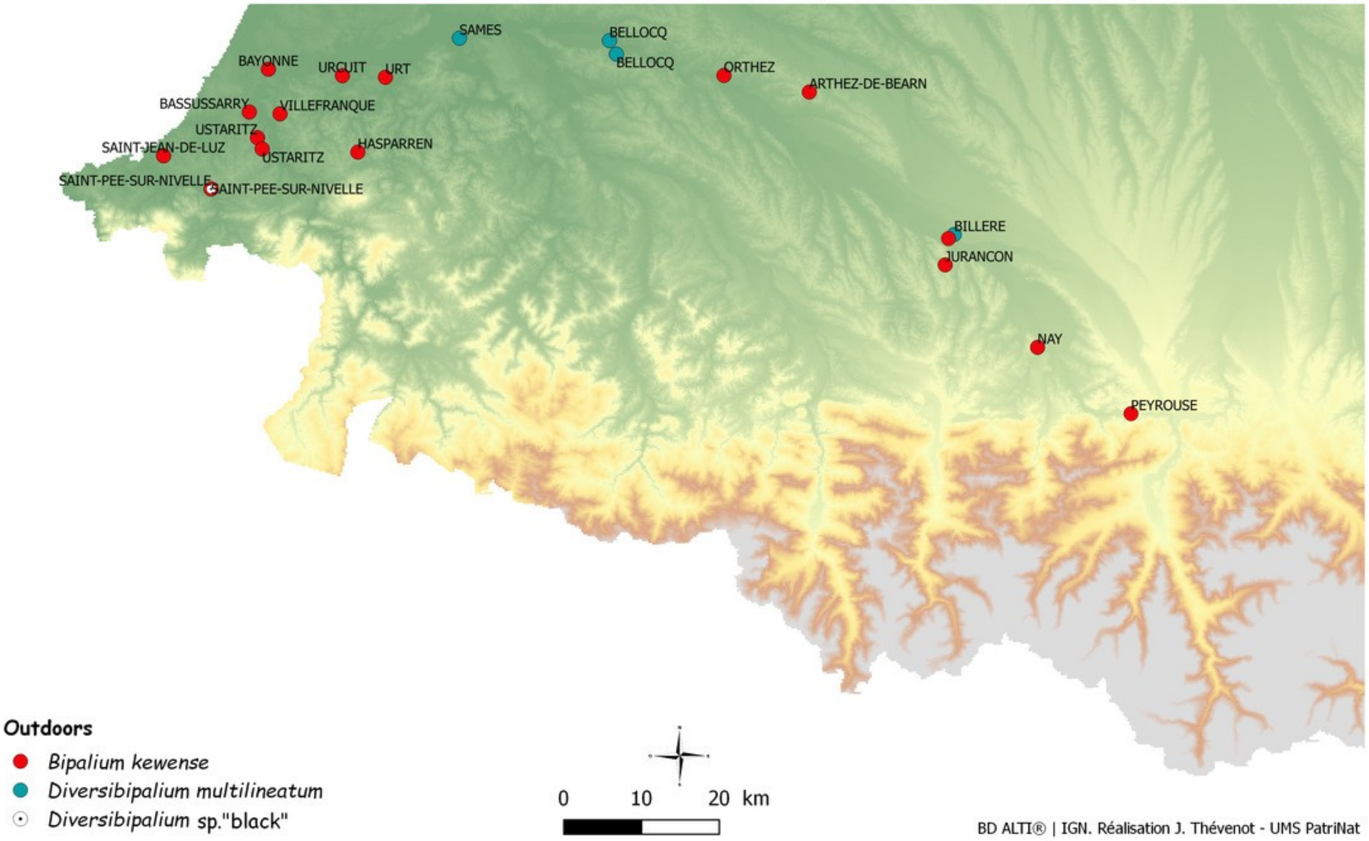

Figure 28. Carte du monde montrant de nouveaux signalements de Plathelminthes bipaliinés dans les territoires français.

Les nouveaux records proviennent de quatre continents (Amérique du Nord, Amérique du Sud, Polynésie, Afrique).

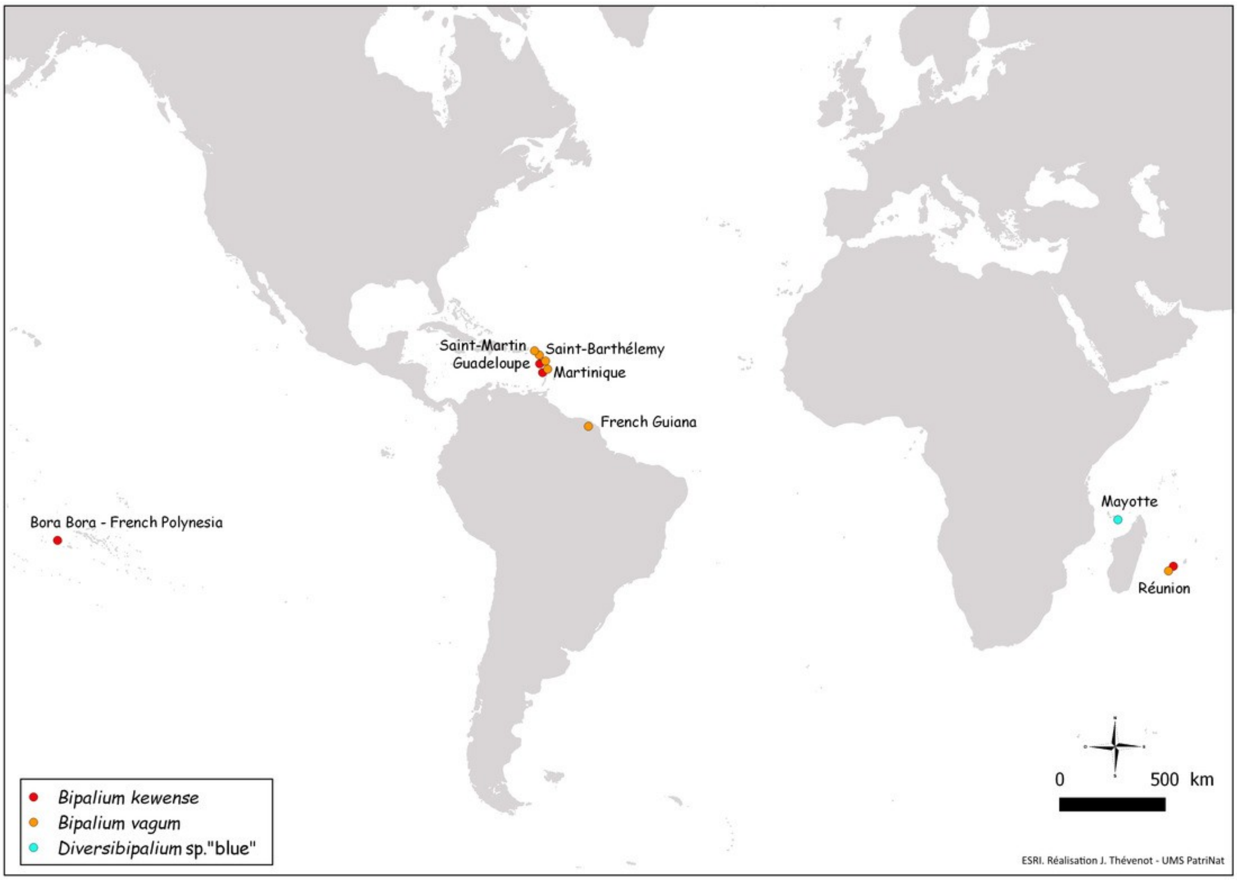

[fin de l'article]
